# Pivotal role of bZIPs in amylose biosynthesis by genome survey and transcriptome analysis in wheat (*Triticum aestivum* L.) mutants

**DOI:** 10.1038/s41598-018-35366-8

**Published:** 2018-11-22

**Authors:** Pankaj Kumar, Ankita Mishra, Himanshu Sharma, Dixit Sharma, Mohammed Saba Rahim, Monica Sharma, Afsana Parveen, Prateek Jain, Shailender Kumar Verma, Vikas Rishi, Joy Roy

**Affiliations:** 1 National Agri-Food Biotechnology Institute (NABI), Sector-81, SAS Nagar, Mohali, 140306 Punjab India; 2 Department of Biotechnology, Panjab University, Chandigarh, 160014 India; 3 Centre for Computational Biology and Bioinformatics, School of Life Sciences, Central University of Himachal Pradesh, Kangra, 176206 Himachal Pradesh India; 4 Department of Plant Sciences, School of Basic and Applied Sciences, Central University of Punjab, Bathinda, 151001 India

## Abstract

Starch makes up 70% of the wheat grain, and is an important source of calories for humans, however, the overconsumption of wheat starch may contribute to nutrition-associated health problems. The challenge is to develop resistant starch including high amylose wheat varieties with health benefits. Adapting advance genomic approaches in EMS-induced mutant lines differing in amylose content, basic leucine zipper (bZIP) regulatory factors that may play role in controlling amylose biosynthesis were identified in wheat. bZIP transcription factors are key regulators of starch biosynthesis genes in rice and maize, but their role in regulating these genes in wheat is poorly understood. A genome-wide survey identified 370 wheat bZIPs, clustered in 11 groups, showing variations in amino acids composition and predicted physicochemical properties. Three approaches namely, whole transcriptome sequencing, qRT-PCR, and correlation analysis in contrasting high and low amylose mutants and their parent line identified 24 candidate bZIP (positive and negative regulators), suggesting bZIPs role in high amylose biosynthesis. bZIPs positive role in high amylose biosynthesis is not known. *In silico* interactome studies of candidate wheat bZIP homologs in *Arabidopsis* and rice identified their putative functional role. The identified bZIPs are involved in stress-related pathways, flower and seed development, and starch biosynthesis. An in-depth analysis of molecular mechanism of novel candidate bZIPs may help in raising and improving high amylose wheat varieties.

## Introduction

Cereal grains largely contain starch^1,2^, and are important sources of calories for humans. Starch is highly digestible, about 99% is digested in human gut and converted into glucose, raising the glycemic index. Its overconsumption causes nutrition-associated health problems^3–5^. There is a surge to develop cereal crops with resistant starch and food grains rich in dietary fibre^6,7^. Resistant starch is categorized as ‘good dietary fibre’^3^. Due to its slow digestion in gut, high amylose starch is categorized as resistant or healthy starch that results in low glycemic index. Amylose is further converted into short chain fatty acids (SCF) by bacteria in large intestine^4,5^. SCFs are known prebiotics with proven health benefits^4,5^. Functional genomics approaches are used to identify regulatory factors controlling amylose biosynthesis that may be manipulated to increase its content in grains^8–10^. In this study, genome-wide analysis of contrasting mutant lines for amylose content led to the identification of candidate bZIPs that correlate with the expression of two key genes of amylose biosynthesis pathway i.e., Granule-bound starch synthase I (GBSSI) and starch branching enzyme II (SBEII).

Briefly, starch is a semi-crystalline structure composed of two fractions, amylose and amylopectin^11,12^. Amylose is largely linear chain of glucose moiety whereas amylopection is highly branched. Amylose is a non-digestible or slow-digestible fraction of starch and thus considered as resistant starch. In cereal, amylose makes ~25% of total starch. In this study, two contrasting mutant lines differing in amylose content were used for genome-wide analysis. High amylose mutant line ‘TAC 75’ with 65% amylose content and low amylose mutant line ‘TAC 6’ with 7% amylose content along with the parent line ‘C 306’ with ~26% amylose content were investigated. The key enzymes involved in starch biosynthesis are ADP-glucose pyrophosphorylase, starch synthases (soluble and granule-bound), and starch branching and debranching enzymes^11–13^. GBSSI is largely responsible for amylose biosynthesis^14,15^, whereas SBEII is responsible for amylopectin biosynthesis^16,17^. The differential expression of GBSSI and SBEII have been correlated with high amylose biosynthesis^18,19^. Unlike in other plants, their regulation by bZIPs is largely unknown in wheat. bZIP transcription factor family members are well-known for their roles in growth and development^20,21^. bZIPs are also involved in the regulation of starch biosynthesis in the endosperm that determine starch quality and quantity^22–25^. OsbZIP58 in rice^23^, ZmbZIP91 in maize^26^ and bZIP58 in wheat^18^ are reported to regulate starch biosynthesis. However, their role in amylose biosynthesis in wheat is not well-understood.

Genome-wide analysis identified many bZIP family members (genes) in various plant species like maize^27^, cucumber^28^, rice^29^ and *Arabidopsis*^30^. For example, there are 94 bZIPs identified in *Oryza sativa subsp*. *indica*, 140 in *O*. *sativa subsp*. *japonica*, 216 in *Zea mays*, 127 in *Arabidopsis thaliana*, and 187 in wheat^31^. Due to its large genome size and lack of complete genome information, the structural and functional characterization of bZIPs is lacking in wheat. Publically available genome sequence of wheat can be used for the genome-wide analysis of bZIPs and their roles in high amylose biosynthesis. The sequence based structured domain information of bZIPs can be used to identify their putative functional role by *in silico* analysis using the published validated functional roles, phylogenetic group information, and interactome analysis in the other plant species such as *Arabidopsis*.

In the present study, genome-wide analysis and phylogenetic analysis of bZIPs were undertaken in wheat. The 284 Gb transcriptome sequence data was generated from the two contrasting mutant lines, ‘TAC 75’ and ‘TAC 6’ and their parent variety, ‘C 306’. Transcriptome analysis, qRT-PCR data, and correlation analysis identified candidate wheat bZIPs (TabZIPs) regulating high amylose biosynthesis. The putative functional role of the candidate TabZIPs in amylose biosynthesis were predicted by using phylogenetic group information and protein interacting networks of *Arabidopsis* databases.

## Results

### Identification and characterization of wheat bZIPs

#### Genome survey of bZIP transcription factors in wheat

Using whole genome sequence databases, wheat bZIPs TFs were identified through sequence similarity match with maize, rice, barley and *Arabidopsis*. A total of 370 wheat bZIPs (TabZIPs) were identified by a Hidden Markov Model (HMM) profile ‘PF00170’ search against the whole wheat proteome ensembl database by HMMER3.0 and BLASTp search using plant bZIP sequences. Subsequently, after validating the integrity of the bZIP domain using NCBI-CDD and InterproScan, a total of 370 wheat bZIP proteins encoded by 238 bZIP genes were identified. Each wheat bZIP protein was assigned a unique identifier from TabZIP1 to TabZIP370. The gene isoforms and their proteins were assigned the same gene/protein number with decimal point. The information regarding TabZIP transcription factors is listed in Supplementary Table S1.

#### Phylogenetic analysis of TabZIPs

**Figure 1:**
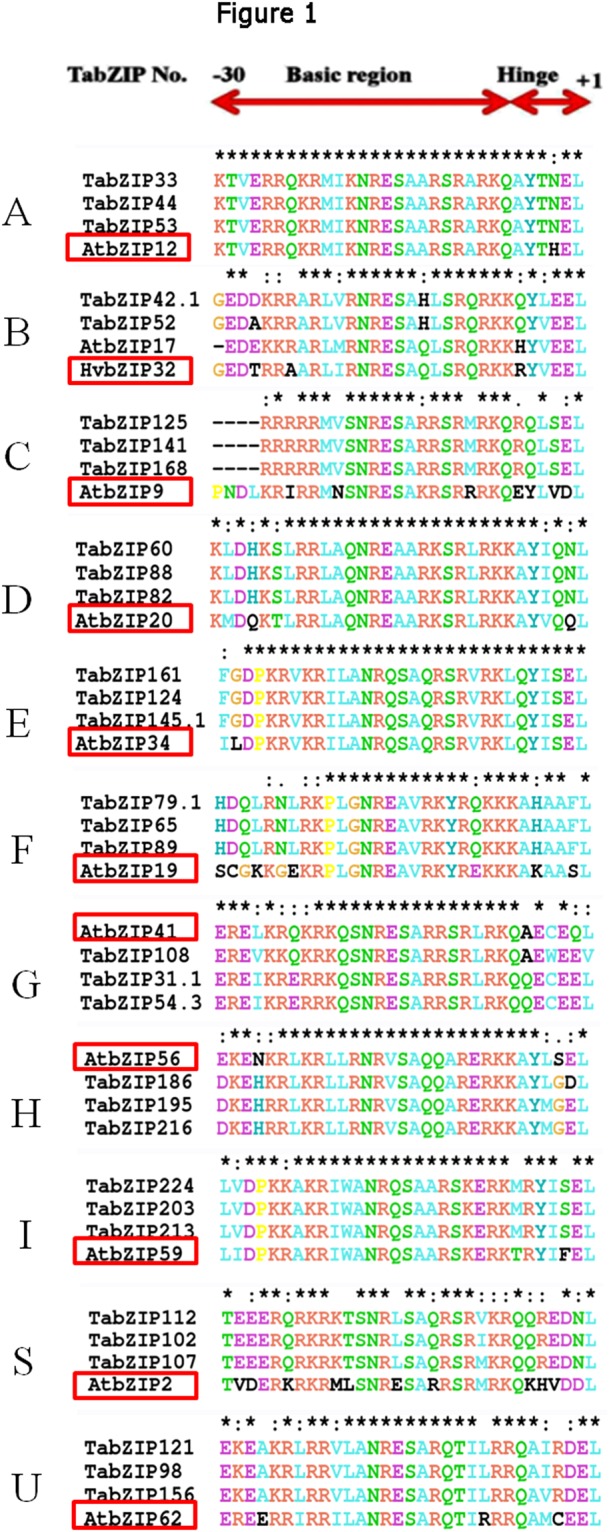
Multiple sequence alignment (MSA) of basic and hinge regions of representative wheat bZIP (TabZIP) proteins. The alignment of the amino acid code was generated using multiple sequence alignment with one homolog of *Arabidopsis* (AtbZIP) or barley (HvbZIP) along with TabZIP. The reference homologs are displayed in red boxes, the asterisk (*) and colon (:) above the alignment represent the conserved and variable region, respectively. The detail of MSA is provided in Supplementary Fig. S1.

**Figure 2:**
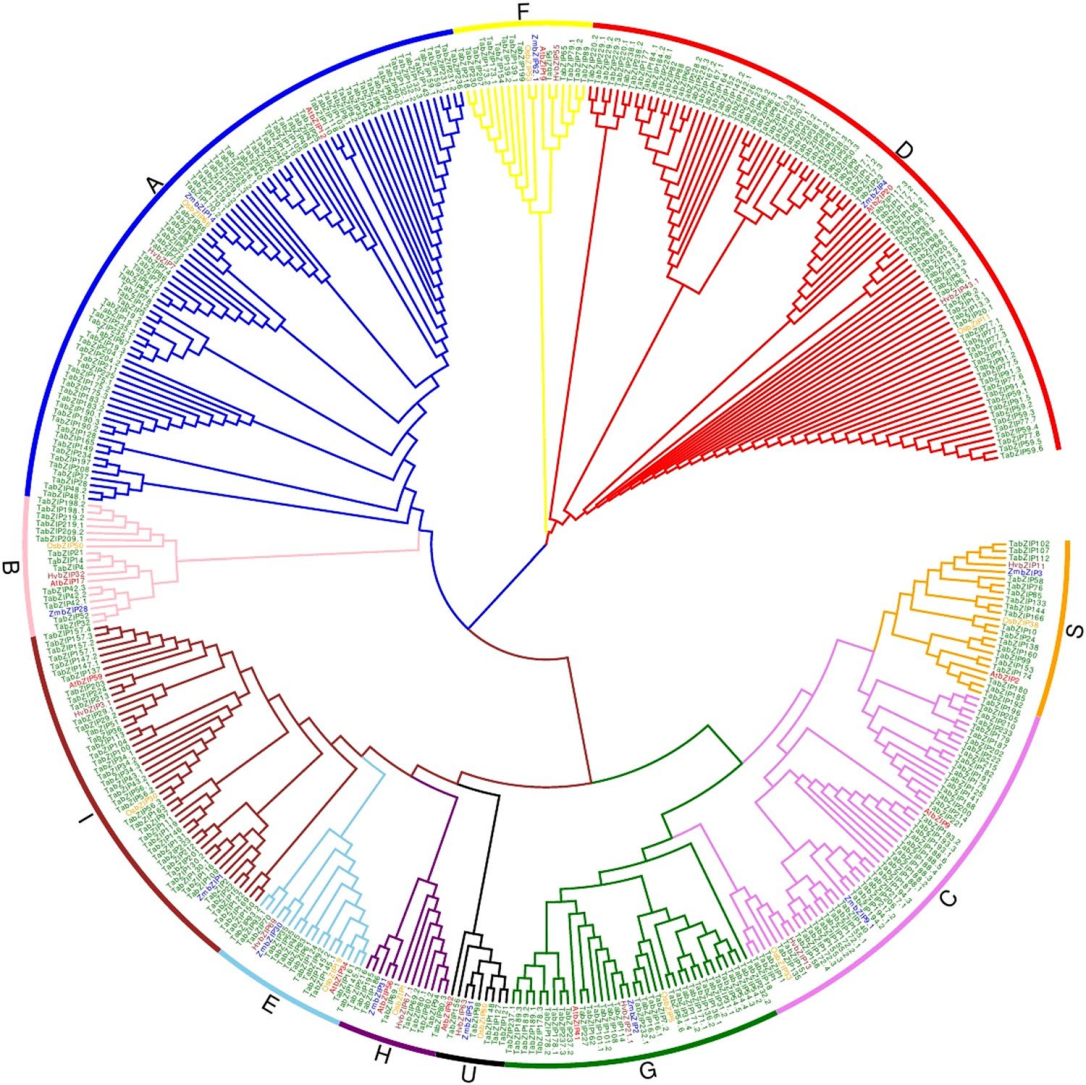
Phylogenetic tree of 370 wheat bZIP (TabZIP) proteins and representative bZIPs of *Arabidopsis* (AtbZIPs), maize (ZmbZIP), barley (HvbZIP), and rice (OsbZIP). The phylogeny was determined by the neighbour joining method using amino acid sequences in MEGA 6.0, at 1000 bootstrap. The tree was divided into 11 clades (**A**–**I** and **S**,**U**), represented by different colors. Details of acronyms used in the tree are given in Supplementary Table S1.

The sequence homology relationship of 370 TabZIP proteins with that of *Arabidopsis*, maize, rice, and barley was analysed by multiple sequence alignment. The analysis revealed a low level of variation (Fig. 1, Supplementary Fig. S1). The phylogenetic analysis grouped the 370 TabZIP proteins in 11 clades (Fig. 2), and named as groups A to I and S to U, in accordance with those reported in *Arabidopsis*^30^. Two groups, A and D, were the largest, each with 83 TabZIPs followed by group C (48 TabZIPs), group I (44 TabZIPs), group G (32 TabZIPs), group S (19 TabZIPs), group B, E and F (each with 14 TabZIPs), group H (9 TabZIPs), while group U is the smallest with only 5 TabZIPs.

#### TabZIP physicochemical properties and conserved motifs analysis

**Figure 3:**
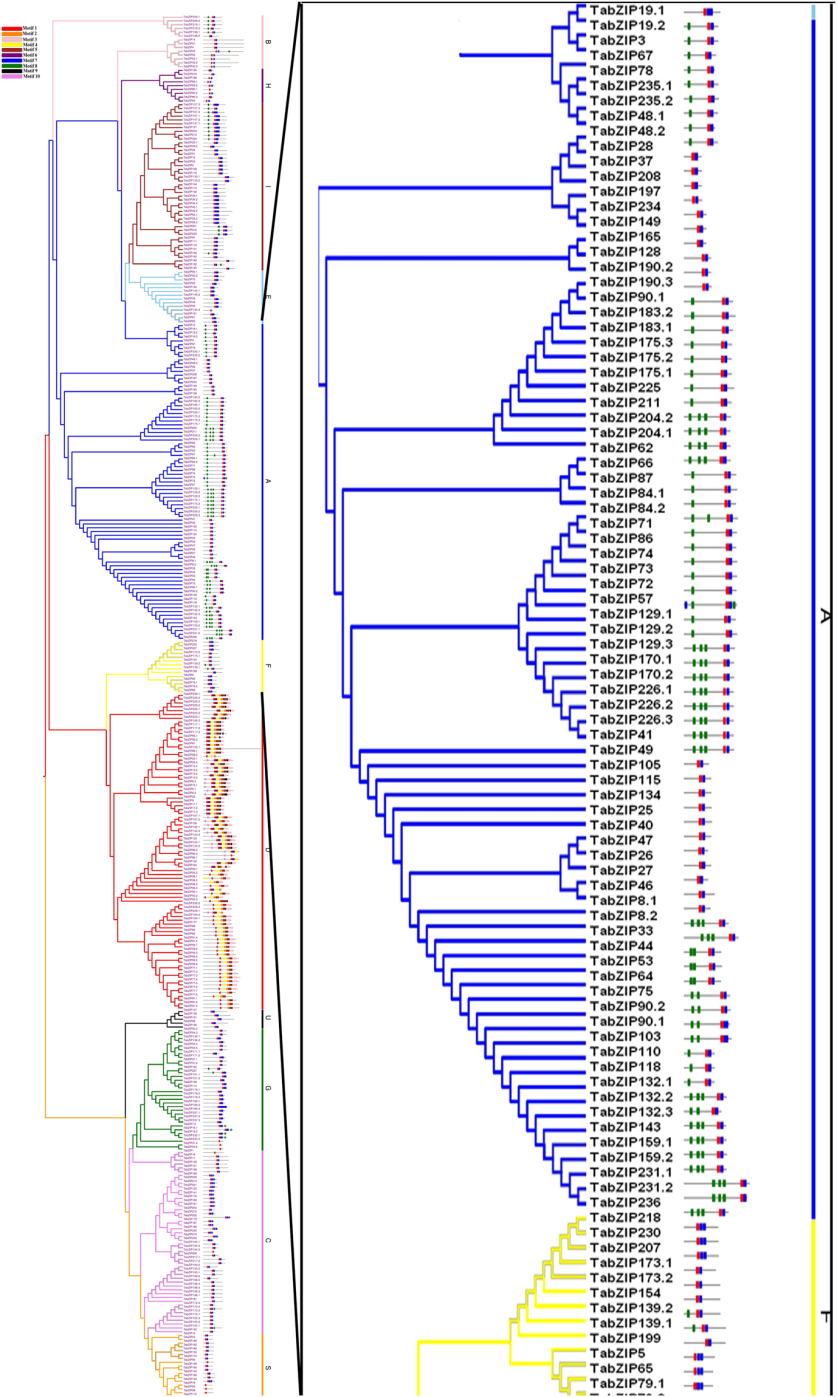
Identification and clustering of the conserved motifs of 370 wheat bZIP (TabZIP) proteins. Motif and phylogenetic analyses of TabZIPs were performed using the MEME database. Based on the phylogenetic relationship, TabZIPs were classified into 11 groups (**A**–**I** and **S**,**U**). The details of the motifs are given in Supplementary Fig. S2.

The 370 predicted TabZIPs varied in their amino acid composition ranging from 129 (TabZIP48.1 and TabZIP94) to 920 (TabZIP106.1) residues with a molecular mass from 14 kDa (TabZIP94) to 103 kDa (TabZIP106.1). Their theoretical isoelectric point (pI) varied between 4.65 (TabZIP198.1 and TabZIP219.1) to 11.38 (TabZIP133). The grand average of hydropathicity (GRAVY) of each amino acid residue in the 370 TabZIP proteins was very low, which indicates better interaction between TabZIP proteins and water molecules. The information regarding the physiochemical properties of TabZIPs is provided in Supplementary Table S2. In TabZIPs, ten motifs were identified by the MEME database that gave insight into their function and divergence (Table 1, Fig. 3, Supplementary Fig. S2, and Supplementary Table S3). Motif 1 was present in all the TabZIP proteins, while motif 7 was present in all the groups except group D. Group D comprised all motifs except motif 7 and 8. Motif 8 was shared by groups A, B, C, F, G and I. The results showed that the TabZIPs share similar sequences and clustered in the same group.

**Table 1:** Details of conserved motifs identified in wheat bZIPs (TabZIP) proteins using MEME database.

Motif number	Multiple consensus sequences	Number of TabZIP proteins with motif
1	[KR]RQ[RK]R[ML][ALI][SQK]NRE[SA]A[RA][RK]SR[LE]RK[QK]AY	365
2	DVFH[LV][LM][ST]G[MA]WA[TS]PAER[CF]F[LF]W[LM]GGFRPSE[LV]LK[IVL]L[IA][GP]	80
3	F[YVL][RQ]QADNLR[QL]QTL[HQ]QM[RH]RILTTRQAAR[CA][FL][LV][SVA][IL][GS][DE]Y[FY][RS]RLRALSSLW[AL][AS]RP	72
4	[MA]FD[MV]EYARW[LV][DE][ED][DH][NG][KR][RH][MIL][AN]ELR[GA][AG][LV][QN]AH[LA][AG]DS[DEN]L[GR]AIV[ED]EC[ML]	82
5	QL[ED]PLTEQQ[LM][MV]GI[CY][NG]LQ[QH]SS[EQ]QAE[ED]AL[SA]QG[LM][QE][QA]L[HQ]QSL[AS][DE]T[VL]A[AS]GTL[NA][DS]G	76
6	[VI]Q[QN]LE[TS]SR[IVL][RK]L[QA]Q[LMI]EQELQRAR[QS]QGI[FL][LI][GS]G[GS]G[AD][GQ]GD[MSL]S[SP][GA]A	82
7	ELER[EKQ]VSXLRAENXXLKX[RQE]LX	268
8	GX[PT][LF][GS]SM[NT][MLV][DE]E[FL][LW]RNIWX[AV]EE	93
9	[NPD][VG][AP]NY[MT][GA][QI]MA[IL]A[LM][GE]KL[AG][STN]LE[SN]	63
10	[AQ][GE][RQ]P[PQ]TL[EN]IFPSWPM[PH]HPQQ[LP]H[SP]	43

A set of 10 motifs were identified in TabZIPs by their consensus sequence analysis. The motif number was assigned following Bailey *et al*. (2009). The detail is provided in Supplementary Table S3.

#### Analysis of cis-regulating elements of starch pathway genes and prediction of DNA binding domain (DBD) of TabZIPs

To understand the mechanism of transcriptional regulation of wheat starch amylose and amylopectin biosynthesis pathway genes, their promoter regions were analysed for the cis-regulating element using the available online wheat genome sequences datasets. It has been previously reported that in plants bZIPs prefer to bind ACGT core sequence like G-box (CACGTG), C-box (GACGTC), and A-box (TACGTA) motifs. Promoter analysis showed the presence of A and G boxes which are the putative sites for bZIP DNA binding domains. To analyse the cis-regulating region, up to 1.0 kb sequence upstream to open reading frames of starch pathways genes (GBSSI, GBSSII, SSI, SSII, SSIII, SSIV, SBEI, SBEIIa and SBEIIb) were identified. Five (GBSSI), seven (GBSSII), four (SSI), four (SSII), six (SSIII), one (SSIV), thirteen (SBEI), six (SBEIIa), and one (SBEIIb) ACGT core sequence were found in the up-stream sequences of these genes. Three A boxes were identified in SSII and one A box was identified in GBSSII and SSIV (Supplementary Fig. S3).

### Identification of candidate TabZIPs regulating amylose biosynthesis in wheat

Three approaches, namely genome-wide transcriptome sequencing, candidate genes based qRT-PCR, and a statistical correlation analysis of GBSSI and SBEII in the contrasting mutant lines for amylose content, were applied for the identification of candidate TabZIPs for high amylose biosynthesis.

#### Sequence characterization and functional analysis of GBSSI, SBEIIa, and SBEIIb in mutants

The sequences of GBSSI, SBEIIa, and SBEIIb, which were retrieved from transcriptome sequence data, identified both synonymous and non-synonymous mutations in their coding regions (Supplementary Fig. S4). The mutations were also detected in their homoeologous loci. Three, one, and two non-synonymous mutation frequency were identified in the catalytic domain of GBSSI-2BL, GBSSI-7AS, and GBSSI-7DS, respectively. In SBEII isoforms (SBEIIa and SBEIIb), mutation frequency was high and the sequencing errors cannot be ruled out. Both SBEII isoforms were located at three homoeologous chromosomes i.e. 2AL, 2BL, and 2DL. The frequency of mutations in SBEIIa and SBEIIb were relatively higher than GBSSI (Supplementary Fig. S4).

The comparative gene expression analysis of GBSSI, SBEIIa and SBEIIb starch metabolic genes at three development stages (21, 28 and 35 days after anthesis, DAA) revealed variation in their expression levels. GBSSI showed high expression during seed development in the high amylose mutant line compared to the low amylose mutant line (Supplementary Fig. S5). During seed development, SBEII isoforms (SBEIIa and SBEIIb) showed very low expression levels in the high amylose mutant line in comparison to that of low amylose mutant line. The higher expression level of GBSSI and lower expression of both isoforms of SBEIIa and SBEIIb in the high amylose mutant line were correlated with increased amylose content during seed development (Supplementary Fig. S5).

#### Transcriptome sequencing of mutant lines

**Figure 4:**
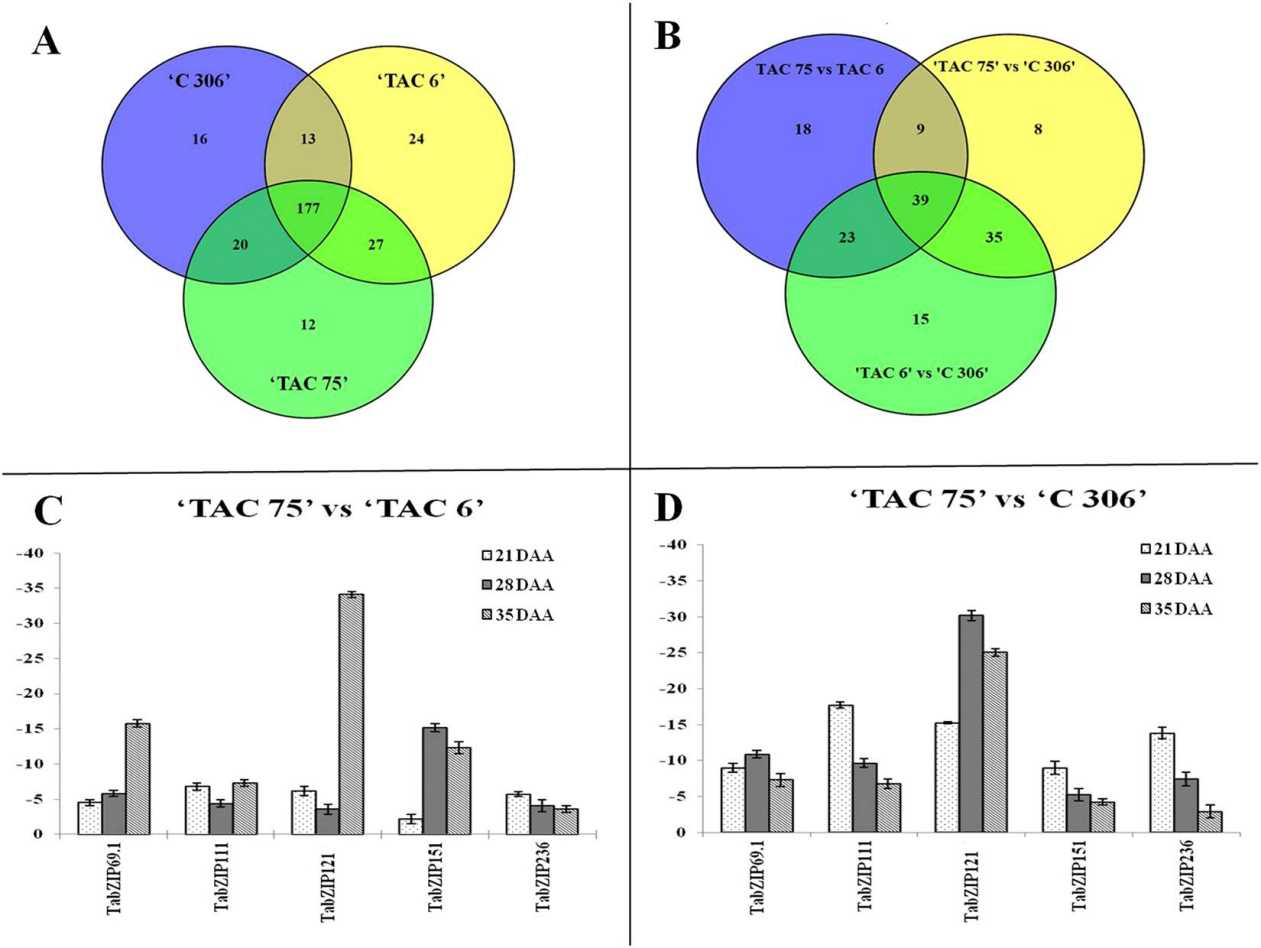
Venn diagrams indicating the number of wheat bZIPs (TabZIPs) showing expression in and among three individual lines (**A**) and bar charts showing differential expression of candidate TabZIPs (**B**). (**A**) Venn diagrams showing the number of bZIPs expressed (FPKM, fragment per kilo per million, ≥0.02) in two mutant lines, ‘TAC 75’ (amylose content = 65%) and ‘TAC 6’ (amylose content = 7%) and the parent variety, ‘C 306’ (amylose content = 26%). (**B**) Comparative analysis of the differentially expressed bZIPs (≥2-fold FPKM) in the three pairs, ‘TAC 75’ vs ‘TAC 6’, ‘TAC 75’ vs ‘C 306’, and ‘TAC 6’ vs ‘C 306’. The overlapped regions show the common TabZIPs. The FPKM values were determined from the NGS sequencing data (mean of two biological replicates). (**C**,**D**) Differential expression level (qRT-PCR) of 5 candidate TabZIPs is represented in ‘TAC 75’ vs ‘TAC 6’ (**C**) and ‘TAC 75 vs ‘C 306’ (**D**). The expression was analysed at three stages of seed development (21, 28 and 35 DAA). ‘TAC 75’, ‘TAC 6’ and ‘C 306’ represent a high amylose mutant line, a low amylose mutant line, and their parent line, respectively. All the data is represented as mean ± SD from three technical replicates.

FPKMs (Fragments Per Kilobase of transcript per Million mapped reads) value is a normalization method for gene expression study. FPKMs of the 370 TabZIP genes were retrieved from the transcriptomic sequence data. The sequence data was generated on two biological replicates of the developing seeds (28 days after anthesis) belonging to the two mutant lines, ‘TAC 75’ with 65% amylose (high amylose mutant line) and ‘TAC 6’ with 7% amylose (low amylose mutant line), and the parent wheat variety, ‘C 306’ with 26% amylose. The genes having FPKM values of at least 0.02 were considered to be expressed and were used for differential gene expression analysis (Supplementary Table S4A, S4B). Considering an FPKM value of 0.02 as the cutoff point, 81 out of 370 (~22%) TabZIPs did not show expression in any three genotypes i.e. 289 (78%) TabZIPs showed expression in at least one genotype at a given time. Individual genotype data showed that 236 (63.8%) TabZIPs showed expression in the high amylose mutant, 241 (63.6%) in the low amylose mutant, and 226 (61.1%) in their parent variety. In pairwise comparison, 177 TabZIPs showed expression in all the three genotypes as shown in the Venn diagram (Fig. 4A). The graph also revealed that 12 TabZIPs were only expressed in the high amylose mutant and therefore unique to the mutant line. The twelve TabZIPs were TabZIP15, TabZIP50.1, TabZIP54.5, TabZIP56.3, TabZIP91.1, TabZIP91.2, TabZIP120.2, TabZIP128, TabZIP167.2, TabZIP173.2, TabZIP184.2, and TabZIP220.2. The FPKM values of these 12 TabZIPs were below 0.5, indicating low expression.

The pairwise differential gene expression analysis of TabZIPs was done among three genotypes (two mutant lines and one parent variety), using log 2-fold of mean FPKM data (two biological replicates). It involved three pairs, ‘TAC 75’ vs ‘TAC 6’, ‘TAC 75’ vs ‘C 306’, and ‘TAC 6’ vs ‘C 306’ (Fig. 4B). Out of the three, only two pairs (‘TAC 75’ vs ‘TAC 6’ and ‘TAC 75’ vs ‘C 306’) were used to identify candidate TabZIPs that may be involved in high amylose biosynthesis by comparing bZIPs expression in the above said genotypes. In comparison, the TabZIP that have FPKM value of zero in other genotype was not taken into consideration as reasons for absence cannot be determined. In this study a relatively stringent criterion of 5-fold differential expression was considered to identify putative candidate TabZIP for high amylose biosynthesis. A total of 147 TabZIPs showed at least 2-fold differential expression among the three pairs, ‘TAC 75’ vs ‘TAC 6’, ‘TAC 75’ vs ‘C 306’, and ‘TAC 6’ vs ‘C 306’ (Fig. 4B).

In the pair, ‘TAC 75’ vs ‘TAC 6’, a total of 89 (39 + 18 + 9 + 23) TabZIPs showed differential expression (at least 2-fold) in the high amylose mutant line (‘TAC 75’) in comparison to the low amylose mutant line (‘TAC 6’) (Fig. 4B, Supplementary Table S5). In this pair, 18 (out of 89) TabZIPs were unique to the pair ‘TAC 75’ vs ‘TAC 6’ (Fig. 4B). Out of 18 unique TabZIPs, two TabZIPs (TabZIP101.1 and TabZIP238.2) showed at least a 5-fold higher and three TabZIPs (TabZIP229.1, TabZIP229.3, and TabZIP238.1) showed at least a 5-fold lower in the high amylose mutant line in comparison to the low amylose mutant line. Out of 18 candidate TabZIPs, five TabZIPs (TabZIP101.1, TabZIP238.2, TabZIP229.1, TabZIP229.3, and TabZIP238.1) may be candidate genes, regulating high amylose biosynthesis.

In the pair, ‘TAC 75’ vs ‘C 306’, a total of 91 (9 + 8 + 39 + 35) TabZIPs showed at least 2-fold differential expression in the high amylose mutant line in comparison to the parent variety. In this pair, 8 TabZIPs were unique to the pair ‘TAC 75’ vs ‘C 306’ (Fig. 4B). Out of 8 unique TabZIP, only one TabZIP i.e. TabZIP145.3 showed at least 5-fold lower expression in the high amylose mutant line in comparison to the parent variety. Therefore, TabZIP145.3 may regulate high amylose biosynthesis.

Between the two pairs involving high amylose mutant line (TAC 75’ vs ‘TAC 6’ and ‘TAC 75’ vs ‘C 306’), 9 TabZIPs were common in both the pairs (Fig. 4B). Out of 9, 7 TabZIPs (TabZIP237.1, TabZIP110, TabZIP157.1, TabZIP188.5, TabZIP194.3, TabZIP117.1, and TabZIP137) showed >5-fold lower expression in the high amylose mutant line in both the pairs. These 7 TabZIPs may also regulate high amylose biosynthesis.

Among the three pairs (‘TAC 75’ vs ‘TAC 6’, ‘TAC 75’ vs ‘C 306’, and ‘TAC 6’ vs ‘C 306’), 39 TabZIPs were common (Fig. 4B). Out of 39, three TabZIPs (TabZIP117.2, TabZIP167.2, and TabZIP184.2) showed at least a 5-fold higher expression in the high amylose line than the low amylose mutant line (‘TAC 6’) and the parent variety (‘C 306’) in the two pairs (‘TAC 75’ vs ‘TAC 6’, and ‘TAC 75’ vs ‘C 306’). These three TabZIPs showed at least 5-fold lower expression in the low amylose mutant line (‘TAC 6’) compared to the parent variety ‘C 306’ (Supplementary Table S5). Similarly, three TabZIPs (TabZIP54.1, TabZIP59.2, and TabZIP77.1) showed at least a 5-fold lower expression in the high amylose line than the low amylose mutant line (‘TAC 6’) and the parent variety (‘C 306’) in the two paired genotypes (‘TAC 75’ vs ‘TAC 6’, and ‘TAC 75’ vs ‘C 306’). These three TabZIPs showed at least 5-fold higher expression in the low amylose mutant line (‘TAC 6’) than the parent variety (Supplementary Table S5). Therefore, these six TabZIPs (TabZIP117.2, TabZIP167.2, TabZIP184.2, TabZIP54.1, TabZIP59.2, and TabZIP77.1) are putative candidate genes for high amylose biosynthesis.

After three pair-wise comparisons, a total of 19 (5 + 1 + 7 + 6) candidate TabZIPs (5 positive regulators: TabZIP101.1, TabZIP117.2, TabZIP167.2, TabZIP184.2, and TabZIP238.2 and 14 negative regulators: TabZIP54.1, TabZIP59.2, TabZIP77.1, TabZIP110, TabZIP117.1, TabZIP137, TabZIP145.3, TabZIP157.1, TabZIP188.5, TabZIP194.3, TabZIP229.1, TabZIP229.3, TabZIP237.1, and TabZIP238.1) were identified for their suggested role in high amylose biosynthesis.

In summary, differential expression (at least 5-fold) analysis of the transcriptome data of the two contrasting mutants and parent varieties identified 19 candidate TabZIPs for high amylose biosynthesis. These TabZIPs may play a pivotal role in the high amylose biosynthesis regulation. The stringent criterion used in this study led to the identification of only few candidate TabZIPs.

#### qRT-PCR-based candidate gene expression analysis in mutant lines

The detailed information on primers designed for qRT-PCR is provided in Supplementary Table S6. Pairwise differential expression analysis of quantitative expression data of the randomly selected 52 (out of 370) TabZIPs was done at three seed developmental stages (21, 28, and 35 DAA) and is given in Supplementary Table S7. The pairwise differential gene expression analysis was done among three genotypes (two mutant lines, ‘TAC 75’ & ‘TAC 6’ and one parent variety, ‘C 306’), using log 2-fold of mean expression data. It involved three pairs, ‘TAC 75’ vs ‘TAC 6’, ‘TAC 75’ vs ‘C 306’, and ‘TAC 6’ vs ‘C 306’ (Supplementary Table S7). Out of the three pairs, only two pairs (‘TAC 75’ vs ‘TAC 6’ and ‘TAC 75’ vs ‘C 306’) are of interest and were analysed further to identify candidate TabZIPs for high amylose biosynthesis by comparing expression of TabZIPs in the high amylose mutant line (‘TAC 75’) in comparison to the low amylose line (‘TAC 6’) and the parent variety (‘C 306’). Differential expression analysis of the 52 TabZIPs revealed largely negative expression in the high amylose mutant line in comparison to the low amylose mutant line (‘TAC 75’ vs ‘TAC 6’) and the parent variety (‘TAC 75’ vs ‘C 306’) (Supplementary Table S7). The majority of the TabZIPs showed a similar pattern during three different seed development stages i.e. 21, 28 and 35 DAA. Differential gene expression analysis revealed five bZIPs (TabZIP69.1, TabZIP111, TabZIP121, TabZIP151, and TabZIP236) that showed low expression in the high amylose line in comparison to the low amylose mutant line (‘TAC 75’ vs ‘TAC 6’) as well as to the parent variety (‘TAC 75’ vs ‘C 306’) (Fig. 4C,D) at the three seed development stages. These five bZIPs may be involved in negative regulation of high amylose biosynthesis. However, two bZIPs, TabZIP50.2 and TabZIP54, showed at least 4-fold positive expression in the high amylose line in comparison to the low amylose mutant line and the parent variety during late stage of seed development i.e. 28 and 35 DAA, the stages at which amylose biosynthesis is very high^18^. Further analysis of differential gene expression data between the high amylose (‘TAC 75’) and low amylose (‘TAC 6’) mutant line showed development stage specific expression, for example, at the 21 DAA seed development stage, 46 TabZIPs showed >2-fold negative expression and 2 TabZIPs showed >2-fold positive expression in the high amylose mutant line. At 28 DAA seed development stage, 46 TabZIPs showed >2-fold negative expression and 5 TabZIPs showed >2-fold positive expression in the high amylose line. At 35 DAA seed development stage, 26 TabZIPs showed >2-fold positive expression and 47 TabZIPs showed >2-fold negative expression. Three biological replications, each with three technical replicates, by and large provided similar results. Therefore, the above analysis identified five bZIPs, TabZIP69.1, TabZIP111, TabZIP121, TabZIP151, and TabZIP236 that showed lower expression in the high amylose line in comparison to the low amylose mutant line and the parent variety (Fig. 4C,D) during the three seed development stages and may be the candidate genes for high amylose biosynthesis. These five TabZIPs are distinct from the 19 TabZIPs identified in the transcriptome sequence data. Therefore, a total 24 candidate TabZIPs regulating high amylose biosynthesis are identified in the two transcriptome studies.

#### Correlation analysis of TabZIPs with key enzymes for amylose biosynthesis

**Figure 5:**
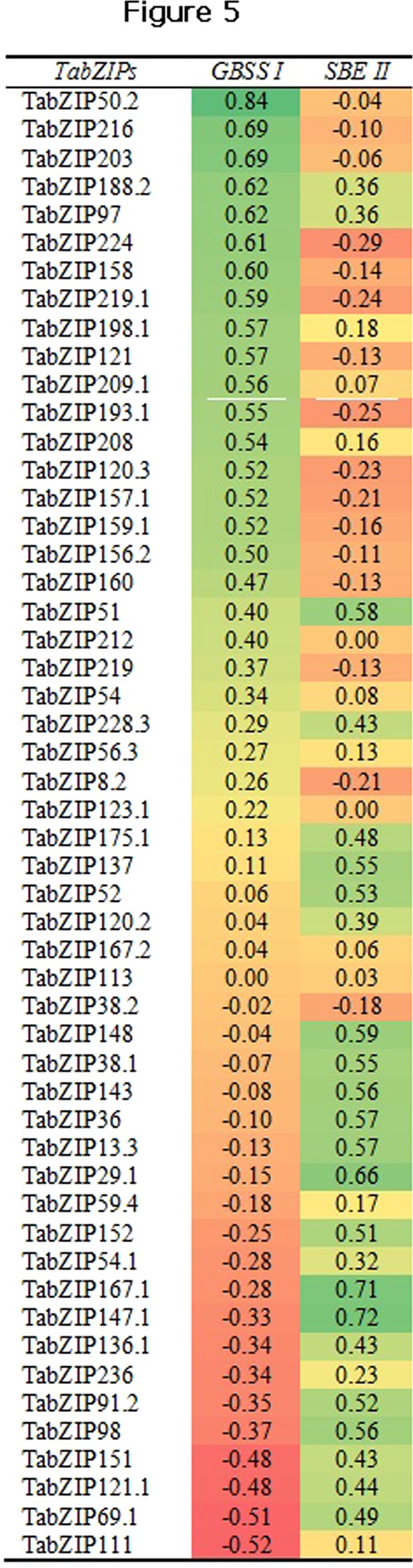
Heatmap of Pearson’s correlation coefficient (*r*) between the expression data of 52 TabZIPs and two starch biosynthesis genes, granule bound starch synthase I (GBSSI), and starch branching enzyme II (SBEIIb). The expression data is the normalized delta C_T_ (ΔCт) values of the genes measured on a quantitative real-time PCR (qRT-PCR). Positive and negative correlations are highlighted in green and red colour, respectively.

Two enzymes, ‘GBSSI’ and ‘SBEIIb’, are key genes mainly responsible for amylose and amylopectin biosynthesis, respectively. The over-expression of GBSSI or down-expression of SBEIIb is functionally validated to high amylose biosynthesis and vice-versa for amylopectin biosynthesis^32^. Therefore, it is important to analyze statistical correlation of expression data of TabZIPs with that of GBSSI and SBEIIb to identify genes responsible for the regulation of the starch biosynthesis pathway. The pairwise statistical correlation analysis of the normalized expression data of the 52 TabZIPs with that of GBSSI and SBEIIb identified 31 TabZIPs showing positive correlation with GBSSI, while 34 of 52 TabZIPs showed positive correlation with SBEIIb (Fig. 5). Among them, 14 TabZIPs showed positive correlation with both enzymes, and therefore, possibly regulate the expression of both GBSSI and SBEIIb. However, no negative correlation was observed for SBEIIb. The correlation analysis identified three TabZIPs (TabZIP 151, TabZIP121, TabZIP69.1) showing moderate negative to moderate positive correlation with GBSSI and SBEIIb, respectively (Fig. 5). The three are candidate genes for high amylose biosynthesis. These three TabZIPs are also present in the identified 24 candidate TabZIPs. In summary, three approaches (transcriptome studies, qRT-PCR analysis, and correlation data) identified 24 candidate TabZIPs for high amylose biosynthesis.

### *In silico* prediction of functional role of candidate TabZIPs regulating high amylose biosynthesis

The functional role of the 24 candidate TabZIPs were predicted by two methods: 1) engaging phylogenetic group information and 2) protein interacting network analysis using *Arabidopsis* databases.

#### Functional predictions using phylogenetic group information

The functional role of the 24 candidate TabZIPs was determined using the functional information of *Arabidopsis* bZIP groups^30^. Their phylogenetic group information (Supplementary Table S1, Supplementary Fig. S2) revealed that the majority of the TabZIPs (10 TabZIPs) belonged to Group D (Table 2). Other TabZIPs belonged to Group C (3 TabZIPs), Group I (3 TabZIP), Groups A (2 TabZIPs), Group G (2 TabZIPs), and one TabZIP each in Groups E, H, and U. However, one TabZIP is not assigned to any group. The 10 TabZIPs of Group D were TabZIP59.2, TabZIP77.1, TabZIP117.1, TabZIP117.2, TabZIP167.2, TabZIP184.2, TabZIP229.1, TabZIP229.3, and TabZIP238.2. The majority of Group D members are involved in plant development and defense^30^. Group I TabZIPs were TabZIP111, TabZIP137, and TabZIP157.1. Group I members are involved in vascular development^33,34^. The Group C TabZIPs were TabZIP151, TabZIP188.5, and TabZIP194.3, and this group shows homology to Opaque 2, which regulates starch and carbohydrate biosynthesis in maize^23^. Group A TabZIPs were TabZIP110 and TabZIP236 and its member are regulators of ABA-mediated signaling pathways and abiotic stress responsive genes. Group G TabZIPs were TabZIP101.1 and TabZIP237.1. Members of this group are reported to be involved in regulation of light-mediated cell elongation. Member of Group E (TabZIP145.3) is reported to be involved in pollen development^35^. Member of Group H (TabZIP69.1) is reported to be involved in systemic acquired resistance^36^. Member of Group U (TabZIP121) are involved in cellular transport and lipid metabolism^37^.

**Table 2:** Detail of 24 candidate TabZIPs including their protein-protein interaction networks and putative predicted functions using *Arabidopsis* databases.

Wheat bZIPs (TabZIPs)	TabZIP group	*Arabidopsis* bZIPs (TabZIP homologs)	Protein-protein interaction (PPI)		Predicted function/role using *in silico* database	PPI network (Refer to Fig. 6)	References of predicted Functional role
			Master regulator	Interacting partners			
TabZIP110	A	bZIP12	AREB3	SNRK2.1, SNRK2.10, DPBF2, NRK2.3	Regulates the expression of stress (abscisic acid) related genes.	N2	Garcia *et al*. 2008
TabZIP236		BZIP66					
TabZIP151	C	bZIP63	BZ02H3	BZIP1, BZIP53, RR18	Regulates seed storage protein expression	N6	Alonso *et al*. 2009
TabZIP188.5		bZIP9	BZIP9	BZIP53, GBF6, BZIP25, BZIP44, ATB2	Regulation of sugar responsive genes	N5	Kang *et al*. 2010
TabZIP194.3							
TabZIP117.2	D	BZIP12	AREB3	SNRK2.1, SNRK2.10, DPBF2, NRK2.3	Regulates the expression of stress (abscisic acid) related genes.	N2	Garcia *et al*. 2008
TabZIP117.1		bZIP29					
TabZIP229.1		BZIP20	PAN	ROXY1, BOP2, NPR1, NPR3, NPR4, LFY	Regulates petal development	N4	Maier *et al*. 2009
Tab ZIP229.3							
TabZIP238.1							
TabZIP238.2							
TabZIP167.2		bZIP65	TGA10	NPR1, NPR4, NPR3, ROXY1, ROXY2, BOP2	Promotes anther development	N3	Murmu *et al*. 2010
TabZIP184.2							
TabZIP59.2		bZIP21	TGA9	NPR3, NPRI, ROXY1, ROXY2		N1	
TabZIP77.1							
TabZIP145.3	E	BZIP61	bZIP34	BZIP43, VIP1, GBF4, ROXY2		N11	
TabZIP101.1	G	bZIP16	BZIP16	GBF1, BZIP68, GBF2, GBF3	Represses the hormone responsive expression of genes in seed development	N13	Schindler *et al*. 1992 and Shen *et al*. 2008
TabZIP237.1							
TabZIP69.1	H	BZIP45	TGA6	NPR1, NPR4, NPR3, ROXY1	Regulates gene expression of mature fruit abscission	N9	Liu *et al*. 2005
TabZIP137	I	bZIP69	AT1G06070	BZIP75, GBF4, BZIP4, FD, BZIP27	Floral pathway development	N7	Abe, Mitsutomo, *et al*. 2005
TabZIP157.1							
TabZIP111	I	BZIP51	VIP1	MYBR1, AGB, BZIP52	Regulates the osmosensory signals of stress (abscisic acid) related genes	N8	Tsugama *et al*. 2012
TabZIP121	U	ND	AT1G19490	BZIP61, BZIP34, BZIP75, BZIP4, BZIP27	Induction of mesoderm and endoderm at earlier embryogenesis	N10	Shen *et al*. 2007
TabZIP54.1	ND	bZIP16	GBF1	HYH, HY5, MYC2, CKA2	Represses the hormone responsive expression genes in seed development	N12	Schindler *et al*. 1992

ND = not determined.

#### Putative functional prediction using protein-protein interaction network analysis

**Figure 6:**
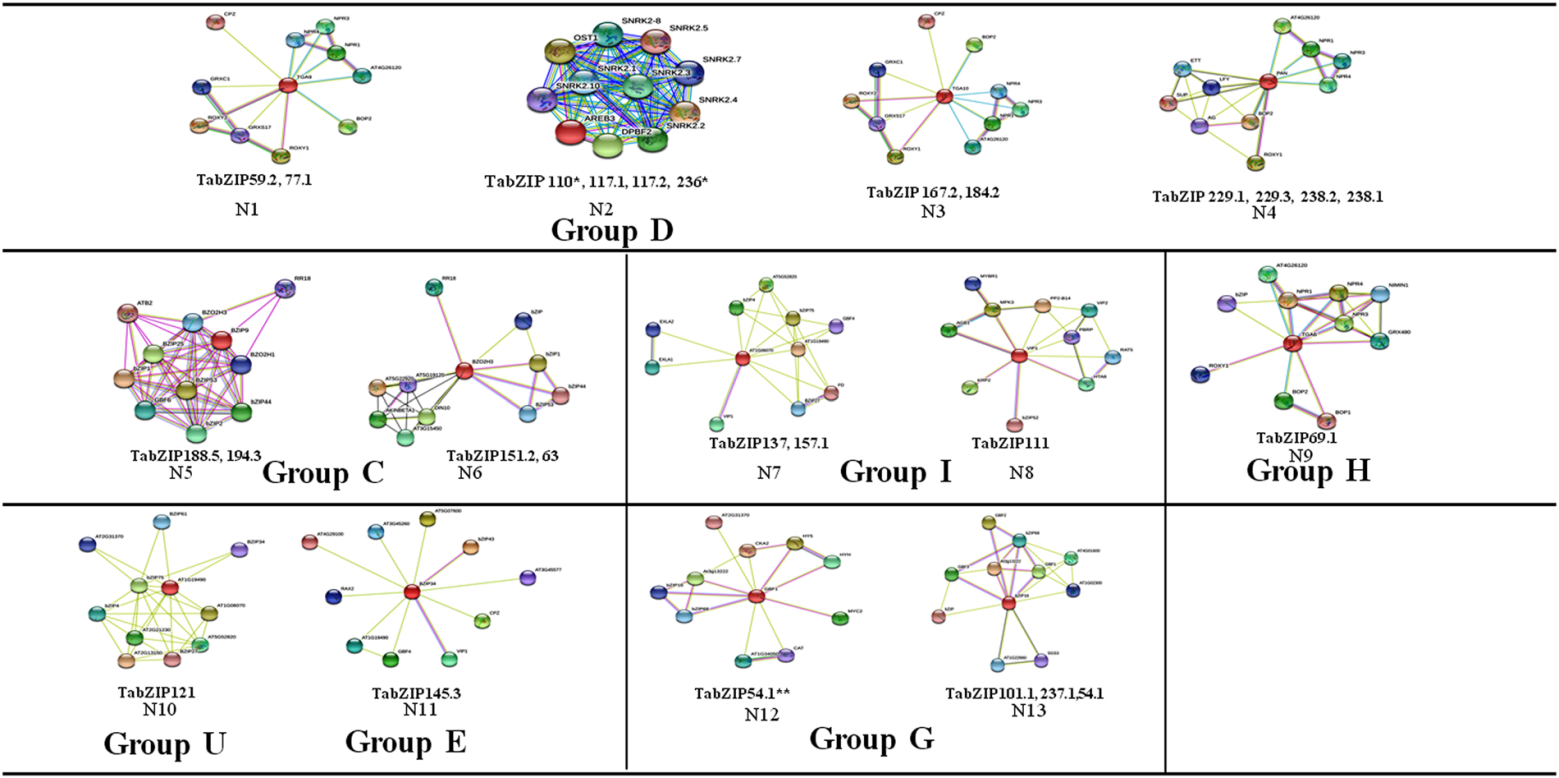
Protein-protein interaction networks (N1 to N13) identified for 24 candidate wheat bZIPs (TabZIPs) controlling high amylose biosynthesis in wheat using *Arabidopsis* databases. The 24 candidate TabZIPs were categorised into 8 wheat bZIP groups (**A**,**C**,**D**,**H**,**I**,**U**,**E**,**G**) and their identified 13 protein-protein interaction (PPI) networks (numbered as N1 to N13). Their interactions were analysed online using STRING database (https://string-db.org/). ‘*’ indicates two TabZIPs (TabZIP110 and TabZIP236) belonging to Group A identified PPI network N2 that is also identified by Group D members. ‘**’ indicates TabZIP54.1 whose wheat bZIP group was not determined.

Due to availability of limited genomic information and protein databases in wheat, the functional role of possible 24 candidate bZIPs regulating high amylose biosynthesis were predicted *in silico*, using their *Arabidopsis* homologs. The study of gene family interaction (protein-protein interaction) is important for prediction of function in biological processes. In this the study, 13 potential protein interaction networks (N1 to N13) were identified for 24 TabZIPs (Table 2, Fig. 6). The master regulators of the 13 networks were GBF1, AREB3, BZ02H3, BZIP9, PAN, TGA10, BZIP34, BZIP16, TGA6, AT1G06070, VIP1, AT1G19490, and TGA9. The four master regulators, TGA6, TGA9, TGA10, and PAN belonged to TGACG (TGA) motif-binding bZIP sub-family. These master regulators are regulating 9 (out of 24) candidate TabZIPs for high amylose biosynthesis. The previous studies reported their high degree of functional redundancy, mainly in plant disease resistance and stress responses. bZIPs like PAN, TGA9 and TGA10 are also involved in flower development. The master regulator, AREB3 regulates 4 TabZIPs (TabZIP110, TabZIP117.1, TabZIP117.2, and TabZIP236) and belonged to ABA-response elements (ABREs) binding proteins (AREB). AREBs are involved in ABA-responsive abiotic stress, mainly drought and high salinity stresses, and are involved in ABA-dependent signal transduction pathway. The functional roles of other master regulators are given in Table 2.

## Discussion

The genome-wide analysis of wheat genome sequence data identified many TabZIPs, which are more than that reported in other major plant species, for example 75 in *Arabidopsis*^30^, 89 in rice^29^, 92 in sorghum^38^, 170 in maize^27^, 121 in banana^39^, 77 in cassava^40^, and 96 in *Brachypodium*^41^. The previous report identified 182 bZIP proteins in wheat^31^. In this study, out of 370, 184 new TabZIPs were identified (Supplementary Tables S1 and S2) and were confirmed by motif and domain analysis. The presence of a large number of TabZIPs in wheat is expected due to its large genome size. bZIP numbers may further increase when the complete reference genome will be available.

Multiple sequence alignment and phylogenetic analysis clustered the wheat bZIP into 11 groups (Figs 1–3 and Supplementary Fig. S1), which is largely in agreement with the previous phylogenetic classification of plant bZIPs. Earlier, major plant bZIPs were grouped in 10–11 groups, for example, 10 groups are reported in *Arabidopsis*^30^ and cassava^40^, and 11 groups in maize^27^, rice^29^, and banana^39^. Earlier bZIPs of wheat and its two wild relatives and other plant species were grouped into 14 groups^31^. In this study, among the eleven phylogenetic groups identified, Group A and D are large cluster groups, each containing 83 TabZIPs. It is reported that bZIPs in Group A play an important role in abscisic acid (ABA) signaling and abiotic stresses. The abiotic stresses and ABA help to induce transcriptional and post-translational regulation^42,43^. Several bZIPs were identified and shown to be ABA-responsive and improve multiple abiotic stress tolerance^42–44^. For example, OsbZIP23, a bZIP from rice, plays a major role in ABA dependent drought and salinity tolerance^43^. Group D members are involved in plant development and defence^30^. For example, TGA (TGACG sequence binding protein) family in *Arabidopsis* interacts with the non-expresser of PR1 (NPR1), which is a key component in the salicylic acid defense signaling pathway^45–49^. The tobacco TGA1 and *Arabidopsis* TGA2 proteins are responsive to salicylic acid and bind to xenobiotic responsive promoters^47–49^.

The functional information on Group B 14 TabZIPs is very limited. The members of this group have trans-membrane domain and specific domain at C-terminus which are important for ER stress response^39^. Group C contains 48 TabZIPs and the members of this group include *Opaque2*, which plays an important role in modulating seed-specific gene expression^30^. *Opaque2* regulates seed storage protein expression by interacting with the PBF protein. Groups E and F each contain 14 TabZIPs, but their functional information is not available. Group G comprises 32 TabZIPs and it is named so due to its members being G-box binding factors^30^. The G-box binding factor genes from *Arabidopsis* are connected to the regulation of light-responsive promoters, and are involved in biotic and abiotic stresses^39,50^. Group H contains nine TabZIPs. The name of this group refers to the HY5 gene which is a bZIP transcription factor in *Arabidopsis* that binds to a G-box, and regulates the stimulus-induced development^30^. The control of HY5 activity by light is also well documented in dark-grown *Arabidopsis*^30,40^. Group I contains 44 TabZIPs. Various studies on Group I genes have been reported from different plant species (Rice RF2a and tomato VSF-1), which indicate their role in vascular development^51,52^.

Group S in wheat is the third smallest group in wheat with 19 TabZIPs, whereas in *Arabidopsis* it is the largest^30^. In *Arabidopsis* the A*TBZIP11/ATB2* gene is regulated by light, and play role in carbohydrate-consuming^53^ (i.e. sink) and in the vascular system^30^. The *ATB2* is involved in post-transcriptional repression by sucrose and carbohydrate balancing^53^. While S Group, in monocot and dicot species get activated during stress^54^. Group U in wheat is the smallest with five TabZIPs, which are very similar to the members of other plant species, such as maize and rice^30^. Members of this group have hydrophobic isoleucine instead of conserved arginine 10 (Supplementary Fig. S1). This substitution in amino acid affects the DNA binding specificity of bZIP, which is documented in earlier studies^55^. This classification was further supported by conserved motif analysis. Conserved motif analysis indicated that almost all the TabZIPs in wheat contained typical bZIP domains. Additionally, each subfamily had some common motifs and some subfamilies also contained the special motifs. These features in conserved bZIP motifs were also observed in maize, rice and *Arabidopsis*. Generally, most TabZIP genes in the same subfamilies showed similar gene structure and conserved motifs, which support their close evolutionary relationship and the classification of subfamilies (Table 1, Fig. 3, and Supplementary Table S3).

As wheat is among the most significant crops, the biological processes like seed development and maturation are vital for starch quality. The bZIP family has been reported to have role in the seed development processes of many plant species, however, the role of bZIPs in amylose or amylopectin biosynthesis regulation is undetermined in wheat. TabZIPs act as positive as well as negative regulators of genes, which actively take part in physiological processes mainly via DNA binding. In the current study, cis-regulatory sequences analysis of the starch biosynthesis pathway indicate the presence of high density of G and A boxes in GBSS I, and SBE II genes (Supplementary Fig. S3), indicating a high probability of bZIPs binding at their promoter regions. Therefore, some may have active role in regulation of amylose and amylopectin biosynthesis.

Before comparative gene expression analysis and next generation sequencing (NGS), the mutation lines were characterized by sequencing two key genes (GBSSI and SBEII) responsible for amylose and amylopectin biosynthesis, both synonymous and non-synonymous mutations were detected in their isoforms. The frequency of mutations in SBEIIa and SBE IIb were relatively higher than GBSSI (Supplementary Fig. S4). The comparative gene expression analysis of GBSSI, SBEIIa, and SBEIIb starch metabolic genes at three development stages (21, 28 and 35 DAA) in mutant lines validated high and low amylose mutants as reported earlier in wheat and other crops^10,12,17,18,56^.

Differential gene expression analysis using whole transcriptome NGS data (two biological replicates) and qRT-PCR data using randomly selected 52 TabZIPs of the two contrasting mutants and parent varieties identified a total of 24 TabZIPs, which are potential candidate genes for high amylose biosynthesis. These TabZIPs may play role in high amylose biosynthesis by either positive or negative regulation. bZIP transcription factors are key regulators of starch biosynthesis genes in rice and maize, but their role in regulating these genes in wheat is poorly understood. It is reported earlier in rice where bZIP transcription factor OsbZIP58^23^ was regulator of six starch metabolic pathway genes including GBSSI and SBEII in starch biosynthesis^23^. The identified candidate TabZIPs were categorized into 8 bZIP groups, namely A, C, D, E, G, H, I and U and one TabZIP (TabZIP54,1) was not assigned to any groups (Table 2). The putative functional role of the 24 candidate TabZIPs were predicted by using phylogenetic group information as well as protein interacting network analysis using *Arabidopsis* databases. Their putative functional role is described in the Results section. The putative functional role can be grouped into four categories- stress (abiotic and biotic), flower and seed development, starch biosynthesis, and seed storage protein regulations.

Interactome analysis identified 13 protein interaction networks (N1 to N13, Fig. 6, Table 2). The master regulators and partners of networks showed that many TabZIPs play important role in biological processes. The four master regulators, TGA6, TGA9, TGA10, and PAN identified in this study belonged to TGACG (TGA) motif-binding bZIP sub-family. These master regulators are represented by 9 (out of 24) candidate TabZIPs for high amylose biosynthesis. Group D TabZIPs (TabZIP59.2, 77.1, 167.2, 184.2) show homology with TGA9 and TGA10 (networks N1 and N4). TGA9 and TGA10 interact with floral glutaredoxins ROXY1 and ROXY2 and are required for anther development^57^. They also interact with other TGAs (TGA4) and PAN^58^. The master regulator of other Group D members (TabZIP 229.1, TabZIP229.3, TabZIP238.1 and TabZIP238.2) in network N4 is PAN (PERIANTHIA). PAN is involved in the determination of floral organ number and also in a post-translational modification by GRXC7/ROXY1^59^. It also binds with BOP1 and BOP2 which are involved in growth asymmetry, an important aspect of patterning in leaves and flowers^60^.

Group I members are involved in vascular development^30,51,52^. TabZIP111 shows homology with VIP1 in network N8 and TabZIP137 and TabZIP157.1 to *Arabidopsis* bZIP (AT1G06070) in network N7 in *Arabidopsis*. They play role in vascular development in tobacco and when over-expressed in *Arabidopsis*, caused growth retardation under a mannitol-stressed condition^61^. Group G members (TabZIP101.1 and 237.1) show homology with GBF and bZIP16 in network N13 (Table 2). GBF2 and AtbZIP16, G-box binding proteins, are involved in the regulation of light or hormone induced stresses^62,63^. Group C members (TabZIP151, 188.5 and 194.3) show homology with BZ02H3 in network N6 and AtbZIP9 in network N5. BZ02H3 regulates seed storage protein expression^24^. Group C TabZIPs also shows homology with *Opaque2*, which is more closely related to monocot species and regulate seed storage protein production by interacting with the PBF protein in *Arabidopsis*’s embryo. Rice OsbZIP58 and OsbZIP20 regulate starch and carbohydrate biosynthesis^23^. The master regulator of Group A members (TabZIP110 and TabZIP236 in network N2) is AREB (ABA-response elements binding proteins) which is induced by ABA and osmotic stresses^64^. AREB is activated by SnRKs and is involved in the PP2C-SnRK-AREB pathway and is an important component of the ABA signalling pathway. The Interactome analysis provides broad sight to understand the regulation and interacting partners’ of these candidate TabZIPs and suggest their putative functions. These wheat bZIPs will be used for validation in molecular breeding programme on large germplasm set through e-QTL analysis and using functional genomics tools.

## Conclusion

High amylose starch is considered to be a good dietary fibre, rich healthy starch, as it is not easily or slowly digested in our guts and is finally transformed into small chain fatty acids (SCFAs) (prebiotics) by bacteria in the large intestine. There is a global demand to develop cereal crops with high levels of resistant starch or dietary fibre rich food grains. Starch is composed of two fractions, amylose and amylopectin, which are synthesised by starch metabolic pathway genes. We identified 370 TabZIP genes from wheat and unravelled their basic classification and evolutionary relationships using evolutionary and conserved protein motif analyses. This will provide ample knowledge for functional characterization of bZIP genes. 24 candidate TabZIPs were identified for high amylose biosynthesis using whole transcriptome data of wheat contrasting mutants for amylose content. Their putative functional roles were determined using protein-protein network analysis. The bZIPs are being used in our lab in molecular breeding for the improvement of amylose content in wheat. All this information will lay a platform for future research on the functional characterization of potential TabZIPs and regulatory mechanism of high amylose biosynthesis in cereal crops. This study therefore is advancing our understanding of the molecular basis of genetic enhancements of amylose content in wheat.

## Materials and Methods

### Plant materials and transcriptome sequence data

Two contrasting mutant lines, ‘TAC 75’ (amylose content ~65%) and ‘TAC 6’ (amylose content ~7%) in M6 generation were used for the identification of putative bZIPs for high amylose biosynthesis. The lines were developed after the EMS treatment of the parent bread wheat (*Triticum aestivum* L.) variety ‘C 306’ (amylose content ~26%)^56^. The 284 Gb transcriptome sequence data was generated from the two biological replicates of two contrasting mutant lines, ‘TAC 75’ and ‘TAC 6’ and their parent variety, ‘C 306’ (unpublished). The whole transcriptome data will be made available by requesting the corresponding author and it is available at NABI’s intranet. For transcriptome sequencing, RNAs were extracted from developing seeds at 28 days after anthesis.

### *In silico* identification, phylogenetic analysis, and physiochemical properties of bZIPs in bread wheat

The identification of the genome wide distribution of bZIPs was performed in two steps. In the first step, the hidden Markov model profiles^65^ of the bZIP domain, *viz*. PF00170^66^, were used as queries to search the bZIP proteins in the wheat proteome Ensembl database (http://plants.ensembl.org/index.html) using HMMER3.0^65^. In the second step, a local BLASTp search was performed to identify the predicted wheat bZIPs by HMMER3.0 with already known bZIPs from *Arabidopsis*^30^, maize^27^, rice^29^ and barley^67^. These potential wheat bZIPs were further examined for the existence and integrity of the bZIP domain by using NCBI-CDD^68^ and InterproScan^69^. The bZIP protein sequences of wheat, rice, maize, barley, and *Arabidopsis* were aligned by using ClustalW^70^ with gap opening and gap extension penalties of 10 and 0.1, respectively. The neighbour-Joining (NJ) method was used to infer the evolutionary history of all bZIP protein sequences. The associated taxa clustered together in the bootstrap test of 1000 replicas. The phylogenetic tree was constructed using MEGA software, version 6^71^. The visualization and annotation of the constructed phylogenetic tree was carried out by using EvolView^72^. The physiochemical properties of protein sequences such as molecular weight, isoelectric point, theoretical pI and GRAVY (Grand average of Hydropathicity) values of TabZIPs were calculated using ExPASy Protparm^73^. MEME (version 4.11.2) was used for the prediction of conserved motifs. The limits specified for minimum width, maximum width, and maximum numbers of motifs were 8, 50 and 10, respectively. The motifs were numbered according to their order displayed by MEME Suite.

### *In Silico* cis-regulating elements map analysis of starch biosynthesis pathway genes

Cis-regulating elements of starch metabolic pathway genes were analysed to explore the DNA binding domains of bZIPs. The genomic sequences of starch metabolic pathway genes (GBSSSI, GBSSII, SSI, SSII, SSIII, SSIV, SBEI, SBEIIa and SBEIIb) were retrieved from the International Genome Sequencing Consortium database (https://www.wheatgenome.org/) (IWGSC). They were processed through Regulatory Sequence Analysis Tools (RSAT: http://rsat.ulb.ac.be/rsat/)^74^ to determine binding sites using their up to 1000 bp upstream sequences.

### *In Silico* differential gene expression analysis

The transcriptome sequence data of the mutant lines and parent (unpublished) was used for the identification of wheat bZIPs (TabZIPs). The identified bZIPs were further confirmed based on gene ontology and Pfam domain analysis. FPKMs (Fragments per kilobase of transcript per million mapped reads) values of TabZIPs were used for gene expression study. FPKMs of the 370 TabZIP genes were retrieved from the transcriptomic sequence data on two biological replicates of the developing seeds (28 days after anthesis) belonging to the two mutant lines and the parent wheat variety. In this study the TabZIP genes having FPKM values of at least 0.02 were considered to be expressed and used for differential gene expression analysis.

### Gene expression analysis by qRT-PCR during seed development

The primers of 52 TabZIPs along with two key genes (GBSSI and SBEII) of starch metabolic pathway that are largely responsible for amylose and amylopectin biosynthesis were designed using Primer Express Software Tool version 3.0 (Thermo Fisher Scientific, USA). The tagged spikes were harvested at three seed developmental stages i.e. 21, 28, and 35 DAA for RNA extraction by Trizol method and cDNA preparation. The relative expression levels of the target bZIPs were calculated by ^ΔΔCt^ method (Schmittgen and Livak *et al*., 2001). Wheat ADP-Ribosylation Factor, ARF (AB050957.1) was used as an internal control gene for normalization of gene expression data.

### Statistical correlation analysis

Pearson’s correlation analysis was performed between the normalized expression data (qRT-PCR) of 52 TabZIPs with that of SBEIIb and GBSSI (all values taken in this study are normalized Ct values with housekeeping ARF gene).

### Interactome analysis

The putative function was determined for the candidate TabZIPs identified for high amylose biosynthesis through interactome analysis i.e. protein interacting network analysis. Wheat bZIPs homolog’s were determined in *Arabidopsis* and then their protein-protein interaction (PPI) networks were identified in *Arabidopsis thaliana* databases (https://string-db.org/) using default parameters.

## Electronic supplementary material

Supplementary Information

Table S4
