# Supplementary material for: Pivotal role of bZIPs in amylose biosynthesis by genome survey and transcriptome analysis in wheat (Triticum aestivum L.) mutants
Source: Sci Rep. 2018 Nov 22;8:17240. doi: 10.1038/s41598-018-35366-8 (PMC6250691; doi:10.1038/s41598-018-35366-8)
Supplement: Supplementary file 1 — Supplementary Information [file 41598_2018_35366_MOESM1_ESM.pdf]

Pivotal role of bZIPs in amylose biosynthesis by genome survey and transcriptome analysis in wheat (*Triticum aestivum* L.) mutants

Pankaj Kumar[1,2], Ankita Mishra[1,2], Himanshu Sharma[1], Dixit Sharma[3], Mohammed Saba Rahim[1,4], Monica Sharma[1], Afsana Parveen[1,2], Prateek Jain[1,2], Shailender Kumar Verma[3], Vikas Rishi[1], Joy Roy[1]

Supplementary Table S1: Details of 370 wheat bZIPs (TabZIPs) and their isoforms identified in this study including protein length (number of amino acid), and position of bZIP domain. The grouping was done on the basis of phylogenetic analysis (Figure 1). Out of 370, 5 bZIPs were not assigned to any groups. The transcript ID was retrieved by blast search from wheat genome sequence databases of Ensembl Plants.

Wheat bZIP (TabZIP)	Transcript ID	Group	Protein length (aa)	Position of bZIP domain within gene
TabZIP1	TRIAE_CS42_1AL_TGACv1_000470_AA0012860.1	C	328	169-228
TabZIP2	TRIAE_CS42_1AL_TGACv1_000644_AA0016380.1	I	391	242-306
TabZIP3	TRIAE_CS42_1AL_TGACv1_000812_AA0019550.1	A	253	168-234
TabZIP4	TRIAE_CS42_1AL_TGACv1_001007_AA0023470.1	B	648	171-235
TabZIP5	TRIAE_CS42_1AL_TGACv1_001373_AA0029470.1	F	227	67-135
TabZIP6.1	TRIAE_CS42_1AL_TGACv1_001653_AA0033650.1	D	481	167-233
TabZIP6.2	TRIAE_CS42_1AL_TGACv1_001653_AA0033650.2	D	445	131-197
TabZIP6.3	TRIAE_CS42_1AL_TGACv1_001653_AA0033650.3	D	439	125-191
TabZIP7.1*	TRIAE_CS42_1AL_TGACv1_001758_AA0034810.1	-	283	243-269
TabZIP7.2	TRIAE_CS42_1AL_TGACv1_001758_AA0034810.2	G	382	243-307
TabZIP8.1	TRIAE_CS42_1AL_TGACv1_003398_AA0049530.1	A	328	254-326
TabZIP8.2	TRIAE_CS42_1AL_TGACv1_003398_AA0049530.2	A	400	326-398
TabZIP9	TRIAE_CS42_1AS_TGACv1_019004_AA0057730.1	D	335	45-108
TabZIP10	TRIAE_CS42_1AS_TGACv1_019984_AA0073590.1	S	150	24-88
TabZIP11	TRIAE_CS42_1BL_TGACv1_030508_AA0092770.1	C	409	207-271
TabZIP12	TRIAE_CS42_1BL_TGACv1_031237_AA0110100.1	A	252	167-233
TabZIP13.1	TRIAE_CS42_1BL_TGACv1_031428_AA0113850.1	D	439	125-191
TabZIP13.2	TRIAE_CS42_1BL_TGACv1_031428_AA0113850.2	D	411	97-163
TabZIP13.3	TRIAE_CS42_1BL_TGACv1_031428_AA0113850.3	D	445	131-197
TabZIP13.4	TRIAE_CS42_1BL_TGACv1_031428_AA0113850.4	D	481	167-233
TabZIP13.5	TRIAE_CS42_1BL_TGACv1_031428_AA0113850.5	D	482	168-234
TabZIP14	TRIAE_CS42_1BL_TGACv1_031873_AA0122080.1	B	649	172-236
TabZIP15	TRIAE_CS42_1BL_TGACv1_032241_AA0127300.1	I	388	239-303
TabZIP16.1	TRIAE_CS42_1BL_TGACv1_033059_AA0136530.1	G	479	339-403
TabZIP16.2	TRIAE_CS42_1BL_TGACv1_033059_AA0136530.2	G	383	243-307
TabZIP17.1	TRIAE_CS42_1BS_TGACv1_049763_AA0161060.1	D	340	44-107
TabZIP17.2	TRIAE_CS42_1BS_TGACv1_049763_AA0161060.2	D	334	44-107
TabZIP17.3	TRIAE_CS42_1BS_TGACv1_049763_AA0161060.3	D	333	44-107
TabZIP18	TRIAE_CS42_1DL_TGACv1_061220_AA0189160.1	C	403	202-266
TabZIP19.1	TRIAE_CS42_1DL_TGACv1_061988_AA0206970.1	A	252	167-233
TabZIP19.2	TRIAE_CS42_1DL_TGACv1_061988_AA0206970.2	A	235	150-216
TabZIP19.3	TRIAE_CS42_1DL_TGACv1_061988_AA0206970.3	A	226	167-225
TabZIP20.1	TRIAE_CS42_1DL_TGACv1_062019_AA0207490.1	D	443	155-231
TabZIP20.2	TRIAE_CS42_1DL_TGACv1_062019_AA0207490.2	A	424	110-176
TabZIP21	TRIAE_CS42_1DL_TGACv1_062780_AA0219780.1	B	652	175-239
TabZIP22	TRIAE_CS42_1DL_TGACv1_063151_AA0224440.1	I	386	238-302
TabZIP23	TRIAE_CS42_1DS_TGACv1_080292_AA0245420.1	D	338	44-107
TabZIP24	TRIAE_CS42_1DS_TGACv1_081606_AA0261850.1	S	150	24-88
TabZIP25	TRIAE_CS42_2AL_TGACv1_093386_AA0278960.1	A	200	107-171
TabZIP26	TRIAE_CS42_2AL_TGACv1_093662_AA0284750.1	A	178	90-154
TabZIP27	TRIAE_CS42_2AL_TGACv1_093662_AA0284760.1	A	225	103-166
TabZIP28	TRIAE_CS42_2AL_TGACv1_094886_AA0304600.1	A	131	51-116
TabZIP29.1	TRIAE_CS42_2AL_TGACv1_095790_AA0314590.1	I	373	164-228
TabZIP29.2	TRIAE_CS42_2AL_TGACv1_095790_AA0314590.2	A	316	107-171
TabZIP30.1	TRIAE_CS42_2AL_TGACv1_096383_AA0318980.1	D	419	119-179
TabZIP30.2	TRIAE_CS42_2AL_TGACv1_096383_AA0318980.2	D	391	91-151
TabZIP31.1	TRIAE_CS42_2AL_TGACv1_096691_AA0320620.1	G	378	259-323
TabZIP31.2	TRIAE_CS42_2AL_TGACv1_096691_AA0320620.2	G	377	258-322
TabZIP31.3*	TRIAE_CS42_2AL_TGACv1_096691_AA0320620.4	-	313	259-284
TabZIP31.4	TRIAE_CS42_2AL_TGACv1_096691_AA0320620.5	G	313	259-289
TabZIP32	TRIAE_CS42_2AS_TGACv1_113477_AA0356620.1	B	643	192-256

TabZIP33	TRIAE_CS42_2AS_TGACv1_113693_AA0359510.1	A	274	175-237
TabZIP34.1	TRIAE_CS42_2AS_TGACv1_114933_AA0370000.1	I	368	159-223
TabZIP34.2	TRIAE_CS42_2AS_TGACv1_114933_AA0370000.2	I	331	159-223
TabZIP34.3	TRIAE_CS42_2AS_TGACv1_114933_AA0370000.3	I	363	159-223
TabZIP35	TRIAE_CS42_2AS_TGACv1_115473_AA0372450.1	E	306	173-237
TabZIP36	TRIAE_CS42_2BL_TGACv1_129531_AA0387390.1	I	382	168-232
TabZIP37	TRIAE_CS42_2BL_TGACv1_129714_AA0393610.1	A	134	54-119
TabZIP38.1	TRIAE_CS42_2BL_TGACv1_130015_AA0401630.1	D	435	130-190
TabZIP38.2	TRIAE_CS42_2BL_TGACv1_130015_AA0401630.3	D	321	16-76
TabZIP38.3	TRIAE_CS42_2BL_TGACv1_130015_AA0401630.5	D	407	102-162
TabZIP38.4	TRIAE_CS42_2BL_TGACv1_130015_AA0401630.8	D	298	102-162
TabZIP39*	TRIAE_CS42_2BL_TGACv1_130715_AA0416520.1	-	138	107-134
TabZIP40	TRIAE_CS42_2BL_TGACv1_130715_AA0416530.1	A	177	102-162
TabZIP41	TRIAE_CS42_2BL_TGACv1_133905_AA0443370.1	A	183	91-155
TabZIP42.1	TRIAE_CS42_2BS_TGACv1_146410_AA0464450.1	B	430	120-184
TabZIP42.2	TRIAE_CS42_2BS_TGACv1_146410_AA0464450.2	B	570	120-184
TabZIP42.3	TRIAE_CS42_2BS_TGACv1_146410_AA0464450.3	B	441	120-184
TabZIP43.1	TRIAE_CS42_2BS_TGACv1_146702_AA0471100.1	I	370	161-225
TabZIP43.2	TRIAE_CS42_2BS_TGACv1_146702_AA0471100.2	I	466	161-225
TabZIP44	TRIAE_CS42_2BS_TGACv1_147553_AA0484820.1	A	279	180-242
TabZIP45	TRIAE_CS42_2BS_TGACv1_147765_AA0487460.1	E	307	173-237
TabZIP46	TRIAE_CS42_2DL_TGACv1_159083_AA0532000.1	A	196	103-161
TabZIP47	TRIAE_CS42_2DL_TGACv1_159083_AA0532010.1	A	200	105-169
TabZIP48.1	TRIAE_CS42_2DL_TGACv1_159174_AA0533780.1	A	129	51-116
TabZIP48.2	TRIAE_CS42_2DL_TGACv1_159174_AA0533780.2	A	131	51-116
TabZIP49	TRIAE_CS42_2DL_TGACv1_159820_AA0543160.1	A	200	107-171
TabZIP50.1	TRIAE_CS42_2DL_TGACv1_159977_AA0545060.1	D	425	121-174
TabZIP50.2	TRIAE_CS42_2DL_TGACv1_159977_AA0545060.4	D	320	16-69
TabZIP50.3	TRIAE_CS42_2DL_TGACv1_159977_AA0545060.5	D	397	93-146
TabZIP51	TRIAE_CS42_2DL_TGACv1_161713_AA0560210.1	I	383	168-232
TabZIP52	TRIAE_CS42_2DS_TGACv1_177487_AA0578420.1	B	572	121-185
TabZIP53	TRIAE_CS42_2DS_TGACv1_177541_AA0579660.1	A	272	175-237
TabZIP54.1*	TRIAE_CS42_2DS_TGACv1_178263_AA0593550.1	-	299	188-244
TabZIP54.2	TRIAE_CS42_2DS_TGACv1_178263_AA0593550.2	G	377	258-322
TabZIP54.3	TRIAE_CS42_2DS_TGACv1_178263_AA0593550.3	G	378	259-323
TabZIP54.4	TRIAE_CS42_2DS_TGACv1_178263_AA0593550.4	G	379	260-324
TabZIP54.5	TRIAE_CS42_2DS_TGACv1_178263_AA0593550.5	G	378	259-323
TabZIP54.6	TRIAE_CS42_2DS_TGACv1_178263_AA0593550.6	G	323	259-289
TabZIP55	TRIAE_CS42_2DS_TGACv1_178412_AA0595200.1	E	306	173-237
TabZIP56.1	TRIAE_CS42_2DS_TGACv1_178575_AA0597660.1	I	411	159-223
TabZIP56.2	TRIAE_CS42_2DS_TGACv1_178575_AA0597660.2	I	368	159-223
TabZIP56.3	TRIAE_CS42_2DS_TGACv1_178575_AA0597660.3	I	363	159-223
TabZIP57	TRIAE_CS42_3AL_TGACv1_193561_AA0613050.1	A	391	302-367
TabZIP58	TRIAE_CS42_3AL_TGACv1_193880_AA0621650.1	S	154	29-93
TabZIP59.1	TRIAE_CS42_3AL_TGACv1_194119_AA0626840.1	D	524	208-284
TabZIP59.2	TRIAE_CS42_3AL_TGACv1_194119_AA0626840.2	D	447	131-207
TabZIP59.3	TRIAE_CS42_3AL_TGACv1_194119_AA0626840.3	D	527	211-287
TabZIP59.4	TRIAE_CS42_3AL_TGACv1_194119_AA0626840.4	D	573	257-333
TabZIP59.5	TRIAE_CS42_3AL_TGACv1_194119_AA0626840.5	D	570	254-330
TabZIP59.6	TRIAE_CS42_3AL_TGACv1_194119_AA0626840.7	D	520	254-330
TabZIP60	TRIAE_CS42_3AL_TGACv1_194206_AA0628450.1	D	475	186-249
TabZIP61	TRIAE_CS42_3AL_TGACv1_194214_AA0628790.1	E	263	135-199
TabZIP62	TRIAE_CS42_3AL_TGACv1_194390_AA0632360.1	A	383	293-359
TabZIP63	TRIAE_CS42_3AL_TGACv1_195373_AA0648710.1	A	382	293-359
TabZIP64	TRIAE_CS42_3AL_TGACv1_195666_AA0652540.1	A	338	264-336
TabZIP65	TRIAE_CS42_3AL_TGACv1_196209_AA0658260.1	F	214	67-135
TabZIP66	TRIAE_CS42_3AL_TGACv1_197019_AA0664400.1	A	391	293-358
TabZIP67	TRIAE_CS42_3AL_TGACv1_197036_AA0664480.1	A	257	173-238
TabZIP68.1	TRIAE_CS42_3AS_TGACv1_211031_AA0683480.1	D	332	42-105
TabZIP68.2	TRIAE_CS42_3AS_TGACv1_211031_AA0683480.2	D	284	42-105
TabZIP69.1	TRIAE_CS42_3AS_TGACv1_211403_AA0689810.1	H	152	61-125
TabZIP69.2	TRIAE_CS42_3AS_TGACv1_211403_AA0689810.2	H	178	87-151

TabZIP70	TRIAE_CS42_3AS_TGACv1_211574_AA0691710.1	E	306	171-235
TabZIP71	TRIAE_CS42_3B_TGACv1_220594_AA0710270.1	A	391	302-369
TabZIP72	TRIAE_CS42_3B_TGACv1_220594_AA0710310.1	A	380	291-356
TabZIP73	TRIAE_CS42_3B_TGACv1_220594_AA0710320.1	A	390	301-366
TabZIP74	TRIAE_CS42_3B_TGACv1_220944_AA0724530.1	A	387	302-367
TabZIP75	TRIAE_CS42_3B_TGACv1_220946_AA0724560.1	A	343	269-341
TabZIP76	TRIAE_CS42_3B_TGACv1_221145_AA0731790.1	S	149	24-88
TabZIP77.1	TRIAE_CS42_3B_TGACv1_221472_AA0741680.1	D	523	257-333
TabZIP77.2	TRIAE_CS42_3B_TGACv1_221472_AA0741680.2	D	570	254-330
TabZIP77.3	TRIAE_CS42_3B_TGACv1_221472_AA0741680.3	D	596	257-333
TabZIP77.4	TRIAE_CS42_3B_TGACv1_221472_AA0741680.4	D	573	257-333
TabZIP77.5	TRIAE_CS42_3B_TGACv1_221472_AA0741680.5	D	541	257-333
TabZIP77.6	TRIAE_CS42_3B_TGACv1_221472_AA0741680.6	D	557	241-317
TabZIP77.7	TRIAE_CS42_3B_TGACv1_221472_AA0741680.7	D	520	254-330
TabZIP77.8	TRIAE_CS42_3B_TGACv1_221472_AA0741680.8	D	466	150-226
TabZIP78	TRIAE_CS42_3B_TGACv1_221698_AA0747790.1	A	250	166-231
TabZIP79.1	TRIAE_CS42_3B_TGACv1_221777_AA0749730.1	F	213	66-134
TabZIP79.2	TRIAE_CS42_3B_TGACv1_221777_AA0749730.2	F	253	106-174
TabZIP80.1	TRIAE_CS42_3B_TGACv1_222340_AA0762500.1	H	158	87-144
TabZIP80.2	TRIAE_CS42_3B_TGACv1_222340_AA0762500.2	H	179	87-151
TabZIP80.3	TRIAE_CS42_3B_TGACv1_222340_AA0762500.3	H	165	87-151
TabZIP81	TRIAE_CS42_3B_TGACv1_222491_AA0765820.2	D	325	42-105
TabZIP82	TRIAE_CS42_3B_TGACv1_223228_AA0778460.1	D	477	188-251
TabZIP83	TRIAE_CS42_3B_TGACv1_225111_AA0805030.2	E	268	140-204
TabZIP84.1	TRIAE_CS42_3B_TGACv1_227947_AA0825230.1	A	389	302-368
TabZIP84.2	TRIAE_CS42_3B_TGACv1_227947_AA0825230.2	A	383	302-362
TabZIP85	TRIAE_CS42_3DL_TGACv1_249032_AA0835230.1	S	154	29-93
TabZIP86	TRIAE_CS42_3DL_TGACv1_249583_AA0851920.1	A	390	301-366
TabZIP87	TRIAE_CS42_3DL_TGACv1_249704_AA0854830.1	A	396	307-373
TabZIP88	TRIAE_CS42_3DL_TGACv1_250203_AA0864170.1	D	477	188-251
TabZIP89	TRIAE_CS42_3DL_TGACv1_251113_AA0877600.1	F	214	68-136
TabZIP90.1	TRIAE_CS42_3DL_TGACv1_251145_AA0878040.1	A	337	263-335
TabZIP90.2	TRIAE_CS42_3DL_TGACv1_251145_AA0878040.2	A	349	263-331
TabZIP91.1	TRIAE_CS42_3DL_TGACv1_251522_AA0882230.1	D	531	261-337
TabZIP91.2	TRIAE_CS42_3DL_TGACv1_251522_AA0882230.2	D	577	261-337
TabZIP91.3	TRIAE_CS42_3DL_TGACv1_251522_AA0882230.3	D	580	264-340
TabZIP91.4	TRIAE_CS42_3DL_TGACv1_251522_AA0882230.5	D	530	214-290
TabZIP91.5	TRIAE_CS42_3DL_TGACv1_251522_AA0882230.6	D	533	217-293
TabZIP92	TRIAE_CS42_3DL_TGACv1_253126_AA0893500.1	E	268	140-204
TabZIP93.1	TRIAE_CS42_3DS_TGACv1_273046_AA0927740.1	E	305	171-235
TabZIP93.2	TRIAE_CS42_3DS_TGACv1_273046_AA0927740.2	E	339	171-235
TabZIP94	TRIAE_CS42_3DS_TGACv1_273334_AA0930450.1	H	129	38-102
TabZIP95.1	TRIAE_CS42_3DS_TGACv1_274210_AA0935070.1	D	327	42-105
TabZIP95.2	TRIAE_CS42_3DS_TGACv1_274210_AA0935070.2	D	332	42-105
TabZIP96.1	TRIAE_CS42_4AL_TGACv1_288889_AA0960680.1	D	580	436-499
TabZIP96.2	TRIAE_CS42_4AL_TGACv1_288889_AA0960680.2	D	581	437-500
TabZIP96.3	TRIAE_CS42_4AL_TGACv1_288889_AA0960680.3	D	503	437-500
TabZIP97	TRIAE_CS42_4AL_TGACv1_289134_AA0965530.1	I	331	164-228
TabZIP98	TRIAE_CS42_4AL_TGACv1_290310_AA0983850.1	U	398	127-191
TabZIP99	TRIAE_CS42_4AL_TGACv1_292709_AA0999550.1	S	164	52-116
TabZIP100	TRIAE_CS42_4AS_TGACv1_306303_AA1006020.1	I	356	156-220
TabZIP101.1	TRIAE_CS42_4AS_TGACv1_307687_AA1022710.1	G	381	300-364
TabZIP101.2	TRIAE_CS42_4AS_TGACv1_307687_AA1022710.2	G	348	267-331
TabZIP102	TRIAE_CS42_4AS_TGACv1_308096_AA1025750.1	S	167	21-85
TabZIP103	TRIAE_CS42_4AS_TGACv1_308354_AA1027470.1	A	225	151-223
TabZIP104	TRIAE_CS42_4BL_TGACv1_320329_AA1035550.2	I	356	156-220
TabZIP105	TRIAE_CS42_4BL_TGACv1_320366_AA1036870.1	A	201	104-168
TabZIP106.1	TRIAE_CS42_4BL_TGACv1_320443_AA1039420.1	D	920	46-109
TabZIP106.2	TRIAE_CS42_4BL_TGACv1_320443_AA1039420.2	D	334	46-109
TabZIP107	TRIAE_CS42_4BL_TGACv1_320589_AA1043990.1	S	167	21-85
TabZIP108	TRIAE_CS42_4BL_TGACv1_320592_AA1044030.1	G	381	300-364
TabZIP109	TRIAE_CS42_4BL_TGACv1_320759_AA1048010.1	I	400	241-305

TabZIP110	TRIAE_CS42_4BL_TGACv1_321014_AA1053680.1	A	225	151-223
TabZIP111	TRIAE_CS42_4BS_TGACv1_328709_AA1092270.1	I	329	164-228
TabZIP112	TRIAE_CS42_4DL_TGACv1_342413_AA1113000.1	S	167	21-85
TabZIP113	TRIAE_CS42_4DL_TGACv1_342950_AA1125920.1	I	356	156-220
TabZIP114	TRIAE_CS42_4DL_TGACv1_343105_AA1129830.1	G	381	300-364
TabZIP115	TRIAE_CS42_4DL_TGACv1_343148_AA1130650.1	A	203	104-168
TabZIP116	TRIAE_CS42_4DL_TGACv1_343219_AA1131620.1	I	393	240-304
TabZIP117.1	TRIAE_CS42_4DL_TGACv1_343394_AA1133900.1	D	334	46-109
TabZIP117.2	TRIAE_CS42_4DL_TGACv1_343394_AA1133900.2	D	335	46-110
TabZIP117.3	TRIAE_CS42_4DL_TGACv1_343394_AA1133900.6	D	377	46-109
TabZIP118	TRIAE_CS42_4DL_TGACv1_344938_AA1150590.1	A	223	149-221
TabZIP119	TRIAE_CS42_4DS_TGACv1_361639_AA1171000.1	I	330	163-227
TabZIP120.1	TRIAE_CS42_4DS_TGACv1_361940_AA1174570.1	D	540	228-291
TabZIP120.2	TRIAE_CS42_4DS_TGACv1_361940_AA1174570.2	D	541	228-292
TabZIP120.3	TRIAE_CS42_4DS_TGACv1_361940_AA1174570.3	D	539	227-290
TabZIP121	TRIAE_CS42_4DS_TGACv1_362363_AA1179150.1	U	496	222-286
TabZIP122	TRIAE_CS42_5AL_TGACv1_374182_AA1192680.1	I	345	168-232
TabZIP123	TRIAE_CS42_5AL_TGACv1_374195_AA1193190.1	D	518	206-295
TabZIP124	TRIAE_CS42_5AL_TGACv1_374263_AA1195450.1	E	239	108-172
TabZIP125	TRIAE_CS42_5AL_TGACv1_374308_AA1196510.1	C	196	89-153
TabZIP126	TRIAE_CS42_5AL_TGACv1_374428_AA1199910.1	D	467	176-231
TabZIP127	TRIAE_CS42_5AL_TGACv1_374464_AA1200910.1	U	380	114-178
TabZIP128	TRIAE_CS42_5AL_TGACv1_374468_AA1200980.1	A	204	131-198
TabZIP129.1	TRIAE_CS42_5AL_TGACv1_374503_AA1201830.1	A	374	285-350
TabZIP129.2	TRIAE_CS42_5AL_TGACv1_374503_AA1201830.2	A	367	285-353
TabZIP129.3	TRIAE_CS42_5AL_TGACv1_374503_AA1201830.3	A	368	285-350
TabZIP130.1	TRIAE_CS42_5AL_TGACv1_375037_AA1214490.1	I	500	342-406
TabZIP130.2	TRIAE_CS42_5AL_TGACv1_375037_AA1214490.2	I	464	342-406
TabZIP131	TRIAE_CS42_5AL_TGACv1_375049_AA1214770.1	I	332	159-223
TabZIP132.1	TRIAE_CS42_5AL_TGACv1_375281_AA1219050.1	A	313	239-295
TabZIP132.2	TRIAE_CS42_5AL_TGACv1_375281_AA1219050.3	A	275	201-257
TabZIP132.3	TRIAE_CS42_5AL_TGACv1_375281_AA1219050.4	A	310	239-297
TabZIP133	TRIAE_CS42_5AL_TGACv1_375448_AA1221780.1	S	183	60-126
TabZIP134	TRIAE_CS42_5AL_TGACv1_375720_AA1226160.1	A	206	104-168
TabZIP135	TRIAE_CS42_5AL_TGACv1_375799_AA1227320.1	C	392	200-264
TabZIP136.1	TRIAE_CS42_5AL_TGACv1_376455_AA1237180.1	G	317	211-275
TabZIP136.2	TRIAE_CS42_5AL_TGACv1_376455_AA1237180.2	G	356	250-314
TabZIP137	TRIAE_CS42_5AL_TGACv1_376758_AA1240660.1	I	377	173-237
TabZIP138	TRIAE_CS42_5AL_TGACv1_376957_AA1242740.1	S	154	28-92
TabZIP139.1	TRIAE_CS42_5AS_TGACv1_393100_AA1268400.1	F	308	126-193
TabZIP139.2	TRIAE_CS42_5AS_TGACv1_393100_AA1268400.2	F	268	86-153
TabZIP140	TRIAE_CS42_5AS_TGACv1_394198_AA1278690.1	C	183	104-168
TabZIP141	TRIAE_CS42_5BL_TGACv1_404292_AA1294130.1	C	193	89-153
TabZIP142.1	TRIAE_CS42_5BL_TGACv1_404323_AA1295530.1	D	402	111-169
TabZIP142.2	TRIAE_CS42_5BL_TGACv1_404323_AA1295530.2	D	456	165-223
TabZIP143	TRIAE_CS42_5BL_TGACv1_404455_AA1300380.1	A	313	239-295
TabZIP144	TRIAE_CS42_5BL_TGACv1_404662_AA1307660.1	S	186	60-126
TabZIP145.1	TRIAE_CS42_5BL_TGACv1_405507_AA1329020.1	E	196	108-172
TabZIP145.2	TRIAE_CS42_5BL_TGACv1_405507_AA1329020.2	E	239	108-172
TabZIP145.3	TRIAE_CS42_5BL_TGACv1_405507_AA1329020.3	E	153	112-145
TabZIP146	TRIAE_CS42_5BL_TGACv1_405777_AA1335110.1	E	271	150-214
TabZIP147.1	TRIAE_CS42_5BL_TGACv1_405990_AA1338700.1	I	356	152-216
TabZIP147.2	TRIAE_CS42_5BL_TGACv1_405990_AA1338700.2	I	377	173-237
TabZIP148	TRIAE_CS42_5BL_TGACv1_406586_AA1347450.1	U	441	175-239
TabZIP149	TRIAE_CS42_5BL_TGACv1_406690_AA1348690.1	A	199	126-193
TabZIP150	TRIAE_CS42_5BL_TGACv1_407412_AA1356630.1	I	509	367-431
TabZIP151	TRIAE_CS42_5BL_TGACv1_408138_AA1361800.1	C	400	208-272
TabZIP152	TRIAE_CS42_5BL_TGACv1_408841_AA1364640.1	D	525	213-302
TabZIP153	TRIAE_CS42_5BS_TGACv1_423537_AA1378880.1	S	157	26-90
TabZIP154	TRIAE_CS42_5BS_TGACv1_423566_AA1379610.1	F	265	83-150
TabZIP155.1	TRIAE_CS42_5BS_TGACv1_424687_AA1391330.1	C	298	104-168
TabZIP155.2	TRIAE_CS42_5BS_TGACv1_424687_AA1391330.2	C	187	104-168

TabZIP155.3	TRIAE_CS42_5BS_TGACv1_424687_AA1391330.4	C	183	104-168
TabZIP156	TRIAE_CS42_5DL_TGACv1_432931_AA1394910.1	U	252	174-238
TabZIP157.1	TRIAE_CS42_5DL_TGACv1_433182_AA1404910.1	I	376	173-237
TabZIP157.2	TRIAE_CS42_5DL_TGACv1_433182_AA1404910.2	I	340	137-201
TabZIP157.3	TRIAE_CS42_5DL_TGACv1_433182_AA1404910.3	I	359	156-220
TabZIP157.4	TRIAE_CS42_5DL_TGACv1_433182_AA1404910.4	I	238	173-237
TabZIP158	TRIAE_CS42_5DL_TGACv1_433372_AA1411290.1	C	406	215-279
TabZIP159.1	TRIAE_CS42_5DL_TGACv1_433373_AA1411370.1	A	316	239-302
TabZIP159.2	TRIAE_CS42_5DL_TGACv1_433373_AA1411370.2	A	313	239-295
TabZIP160	TRIAE_CS42_5DL_TGACv1_433913_AA1425130.1	S	154	28-92
TabZIP161	TRIAE_CS42_5DL_TGACv1_434170_AA1430970.1	E	239	108-172
TabZIP162	TRIAE_CS42_5DL_TGACv1_434504_AA1437070.1	G	316	219-283
TabZIP163	TRIAE_CS42_5DL_TGACv1_434744_AA1440910.1	I	332	159-223
TabZIP164	TRIAE_CS42_5DL_TGACv1_434960_AA1444170.1	D	437	125-214
TabZIP165	TRIAE_CS42_5DL_TGACv1_435011_AA1444870.1	A	199	133-148
TabZIP166	TRIAE_CS42_5DL_TGACv1_435031_AA1445050.1	S	187	61-127
TabZIP167.1	TRIAE_CS42_5DL_TGACv1_435093_AA1445920.1	D	466	175-230
TabZIP167.2	TRIAE_CS42_5DL_TGACv1_435093_AA1445920.3	D	423	132-187
TabZIP168	TRIAE_CS42_5DL_TGACv1_435926_AA1456400.1	C	193	88-152
TabZIP169	TRIAE_CS42_5DL_TGACv1_436292_AA1459750.1	I	509	367-431
TabZIP170.1	TRIAE_CS42_5DL_TGACv1_436729_AA1462940.1	A	366	283-341
TabZIP170.2	TRIAE_CS42_5DL_TGACv1_436729_AA1462940.2	A	365	283-351
TabZIP171.1	TRIAE_CS42_5DL_TGACv1_437065_AA1464880.1	G	255	149-213
TabZIP171.2	TRIAE_CS42_5DL_TGACv1_437065_AA1464880.2	G	362	256-320
TabZIP172.1	TRIAE_CS42_5DS_TGACv1_456478_AA1471590.1	C	183	104-168
TabZIP172.2	TRIAE_CS42_5DS_TGACv1_456478_AA1471590.2	C	186	104-168
TabZIP172.3	TRIAE_CS42_5DS_TGACv1_456478_AA1471590.3	C	187	104-168
TabZIP172.4	TRIAE_CS42_5DS_TGACv1_456478_AA1471590.4	C	298	104-168
TabZIP173.1	TRIAE_CS42_5DS_TGACv1_456500_AA1472280.1	F	266	84-151
TabZIP173.2	TRIAE_CS42_5DS_TGACv1_456500_AA1472280.2	F	233	84-151
TabZIP174	TRIAE_CS42_5DS_TGACv1_457365_AA1485570.1	S	157	26-90
TabZIP175.1	TRIAE_CS42_6AL_TGACv1_471610_AA1511740.1	A	350	267-332
TabZIP175.2	TRIAE_CS42_6AL_TGACv1_471610_AA1511740.2	A	369	267-332
TabZIP175.3	TRIAE_CS42_6AL_TGACv1_471610_AA1511740.3	A	351	267-332
TabZIP176	TRIAE_CS42_6AL_TGACv1_474358_AA1535080.1	C	206	99-163
TabZIP177	TRIAE_CS42_6AS_TGACv1_485564_AA1547640.1	D	458	149-205
TabZIP178.1	TRIAE_CS42_6AS_TGACv1_486123_AA1557200.1	G	323	227-291
TabZIP178.2	TRIAE_CS42_6AS_TGACv1_486123_AA1557200.2	G	344	248-312
TabZIP178.3	TRIAE_CS42_6AS_TGACv1_486123_AA1557200.3	G	347	251-315
TabZIP179	TRIAE_CS42_6AS_TGACv1_486251_AA1558790.1	C	177	78-142
TabZIP180	TRIAE_CS42_6AS_TGACv1_487131_AA1568440.1	S	174	34-98
TabZIP181	TRIAE_CS42_6AS_TGACv1_488428_AA1575500.1	C	313	134-198
TabZIP182	TRIAE_CS42_6BL_TGACv1_500317_AA1603110.1	C	207	102-166
TabZIP183.1	TRIAE_CS42_6BL_TGACv1_500899_AA1611470.1	A	351	268-333
TabZIP183.2	TRIAE_CS42_6BL_TGACv1_500899_AA1611470.2	A	352	268-333
TabZIP184.1	TRIAE_CS42_6BS_TGACv1_513324_AA1638290.1	D	457	148-224
TabZIP184.2	TRIAE_CS42_6BS_TGACv1_513324_AA1638290.2	D	458	149-225
TabZIP185	TRIAE_CS42_6BS_TGACv1_513373_AA1639310.1	S	175	35-99
TabZIP186	TRIAE_CS42_6BS_TGACv1_513423_AA1641170.1	H	156	80-144
TabZIP187	TRIAE_CS42_6BS_TGACv1_514351_AA1658680.1	C	177	78-142
TabZIP188.1	TRIAE_CS42_6BS_TGACv1_515508_AA1670570.1	C	304	125-189
TabZIP188.2	TRIAE_CS42_6BS_TGACv1_515508_AA1670570.2	C	311	132-196
TabZIP188.3	TRIAE_CS42_6BS_TGACv1_515508_AA1670570.3	C	305	126-190
TabZIP188.4	TRIAE_CS42_6BS_TGACv1_515508_AA1670570.4	C	312	133-197
TabZIP188.5	TRIAE_CS42_6BS_TGACv1_515508_AA1670570.5	C	302	123-187
TabZIP188.6	TRIAE_CS42_6BS_TGACv1_515508_AA1670570.6	C	240	61-125
TabZIP189.1	TRIAE_CS42_6BS_TGACv1_515656_AA1671610.1	G	346	247-311
TabZIP189.2	TRIAE_CS42_6BS_TGACv1_515656_AA1671610.2	G	325	226-290
TabZIP189.3	TRIAE_CS42_6BS_TGACv1_515656_AA1671610.3	G	349	250-314
TabZIP190.1	TRIAE_CS42_6DL_TGACv1_526318_AA1679380.1	A	360	277-342
TabZIP190.2	TRIAE_CS42_6DL_TGACv1_526318_AA1679380.2	A	361	277-342
TabZIP190.3	TRIAE_CS42_6DL_TGACv1_526318_AA1679380.3	A	379	277-342

TabZIP191	TRIAE_CS42_6DL_TGACv1_527360_AA1702780.1	C	199	101-165
TabZIP192	TRIAE_CS42_6DS_TGACv1_542930_AA1732700.1	S	174	36-100
TabZIP193.1	TRIAE_CS42_6DS_TGACv1_545295_AA1750190.1	C	313	134-198
TabZIP193.2	TRIAE_CS42_6DS_TGACv1_545295_AA1750190.2	C	233	54-118
TabZIP193.3	TRIAE_CS42_6DS_TGACv1_545295_AA1750190.3	C	312	133-197
TabZIP193.4*	TRIAE_CS42_6DS_TGACv1_545295_AA1750190.4	-	171	3-56
TabZIP194.1	TRIAE_CS42_7AL_TGACv1_556285_AA1759970.1	C	250	143-207
TabZIP194.2	TRIAE_CS42_7AL_TGACv1_556285_AA1759970.2	C	251	144-208
TabZIP194.3	TRIAE_CS42_7AL_TGACv1_556285_AA1759970.3	C	193	147-182
TabZIP195	TRIAE_CS42_7AL_TGACv1_556796_AA1770930.1	H	164	82-146
TabZIP196	TRIAE_CS42_7AL_TGACv1_557050_AA1775820.1	C	242	145-209
TabZIP197	TRIAE_CS42_7AL_TGACv1_557464_AA1781650.1	A	164	90-154
TabZIP198.1	TRIAE_CS42_7AL_TGACv1_558306_AA1792200.1	B	302	139-203
TabZIP198.2	TRIAE_CS42_7AL_TGACv1_558306_AA1792200.2	B	253	90-154
TabZIP199	TRIAE_CS42_7AL_TGACv1_558616_AA1794710.1	F	306	151-218
TabZIP200	TRIAE_CS42_7AS_TGACv1_569208_AA1810370.1	C	217	105-176
TabZIP201	TRIAE_CS42_7AS_TGACv1_569238_AA1811090.1	I	467	314-378
TabZIP202	TRIAE_CS42_7AS_TGACv1_569350_AA1814160.1	C	185	84-149
TabZIP203	TRIAE_CS42_7AS_TGACv1_569625_AA1820550.1	I	365	167-231
TabZIP204.1	TRIAE_CS42_7AS_TGACv1_571414_AA1847500.1	A	343	259-324
TabZIP204.2	TRIAE_CS42_7AS_TGACv1_571414_AA1847500.2	A	343	259-324
TabZIP205	TRIAE_CS42_7BL_TGACv1_576759_AA1854020.1	C	167	70-134
TabZIP206	TRIAE_CS42_7BL_TGACv1_577418_AA1875080.1	C	247	140-204
TabZIP207	TRIAE_CS42_7BL_TGACv1_577539_AA1878150.1	F	255	91-158
TabZIP208	TRIAE_CS42_7BL_TGACv1_577569_AA1878740.1	A	165	91-155
TabZIP209.1	TRIAE_CS42_7BL_TGACv1_579567_AA1908510.1	B	302	138-203
TabZIP209.2	TRIAE_CS42_7BL_TGACv1_579567_AA1908510.2	B	293	129-194
TabZIP210	TRIAE_CS42_7BL_TGACv1_580489_AA1914260.1	C	247	150-221
TabZIP211	TRIAE_CS42_7BS_TGACv1_591894_AA1924840.1	A	340	256-321
TabZIP212	TRIAE_CS42_7BS_TGACv1_592661_AA1942670.1	I	468	314-378
TabZIP213	TRIAE_CS42_7BS_TGACv1_592867_AA1945500.1	I	370	171-235
TabZIP214	TRIAE_CS42_7BS_TGACv1_593422_AA1951360.1	C	223	104-168
TabZIP215	TRIAE_CS42_7BS_TGACv1_593476_AA1951870.1	C	184	83-148
TabZIP216	TRIAE_CS42_7DL_TGACv1_603253_AA1979190.1	H	207	127-191
TabZIP217.1	TRIAE_CS42_7DL_TGACv1_603981_AA1991930.1	C	349	242-306
TabZIP217.2	TRIAE_CS42_7DL_TGACv1_603981_AA1991930.2	C	348	241-305
TabZIP218	TRIAE_CS42_7DL_TGACv1_604895_AA2002830.1	F	250	86-153
TabZIP219.1	TRIAE_CS42_7DL_TGACv1_606407_AA2009990.1	B	302	138-203
TabZIP219.2	TRIAE_CS42_7DL_TGACv1_606407_AA2009990.2	B	278	114-179
TabZIP220.1	TRIAE_CS42_7DS_TGACv1_621925_AA2029490.1	D	418	130-224
TabZIP220.2	TRIAE_CS42_7DS_TGACv1_621925_AA2029490.3	D	448	160-254
TabZIP221	TRIAE_CS42_7DS_TGACv1_622069_AA2032220.1	C	370	171-235
TabZIP222	TRIAE_CS42_7DS_TGACv1_623432_AA2053120.1	C	446	345-410
TabZIP223	TRIAE_CS42_7DS_TGACv1_624207_AA2059750.1	I	473	314-378
TabZIP224	TRIAE_CS42_7DS_TGACv1_625219_AA2064590.1	I	368	170-234
TabZIP225	TRIAE_CS42_7DS_TGACv1_625303_AA2064840.1	A	345	261-326
TabZIP226.1	TRIAE_CS42_U_TGACv1_640702_AA2069780.1	A	365	283-351
TabZIP226.2	TRIAE_CS42_U_TGACv1_640702_AA2069780.2	A	366	283-341
TabZIP226.3	TRIAE_CS42_U_TGACv1_640702_AA2069780.3	A	368	283-348
TabZIP227	TRIAE_CS42_U_TGACv1_640756_AA2072630.1	G	384	291-355
TabZIP228.1	TRIAE_CS42_U_TGACv1_641199_AA2087990.1	D	324	148-224
TabZIP228.2	TRIAE_CS42_U_TGACv1_641199_AA2087990.2	D	457	148-224
TabZIP228.3	TRIAE_CS42_U_TGACv1_641199_AA2087990.3	D	458	149-225
TabZIP229.1	TRIAE_CS42_U_TGACv1_641253_AA2089870.1	D	496	208-302
TabZIP229.2	TRIAE_CS42_U_TGACv1_641253_AA2089870.2	D	418	130-224
TabZIP229.3	TRIAE_CS42_U_TGACv1_641253_AA2089870.3	D	448	160-254
TabZIP230	TRIAE_CS42_U_TGACv1_641258_AA2089960.1	F	253	89-156
TabZIP231.1	TRIAE_CS42_U_TGACv1_641308_AA2091490.2	A	483	409-481
TabZIP231.2	TRIAE_CS42_U_TGACv1_641308_AA2091490.3	A	464	409-464
TabZIP232.1	TRIAE_CS42_U_TGACv1_641545_AA2097630.1	G	382	243-307
TabZIP232.2	TRIAE_CS42_U_TGACv1_641545_AA2097630.2	G	328	243-290
TabZIP233	TRIAE_CS42_U_TGACv1_642196_AA2113120.1	C	167	67-131

TabZIP234	TRIAE_CS42_U_TGACv1_642368_AA2116410.1	A	164	90-154
TabZIP235.1	TRIAE_CS42_U_TGACv1_643015_AA2126130.1	A	228	166-222
TabZIP235.2	TRIAE_CS42_U_TGACv1_643015_AA2126130.2	A	250	166-231
TabZIP236	TRIAE_CS42_U_TGACv1_643396_AA2131620.1	A	327	253-325
TabZIP237.1	TRIAE_CS42_U_TGACv1_645006_AA2142650.1	G	321	224-288
TabZIP237.2	TRIAE_CS42_U_TGACv1_645006_AA2142650.2	G	345	248-312
TabZIP237.3	TRIAE_CS42_U_TGACv1_645006_AA2142650.4	G	317	220-284
TabZIP238.1	TRIAE_CS42_U_TGACv1_645734_AA2145290.1	D	413	125-201
TabZIP238.2	TRIAE_CS42_U_TGACv1_645734_AA2145290.2	D	443	155-231

Supplementary Table S2: Details of the wheat bZIP (TabZIP) proteins including protein length, molecular weight, theoretical isoelectric point, and GRAVY value.

TabZIP	Transcript ID	Protein length (amino acids)	Molecular Weight (Dalton)	Theoretical isoelectric point	GRAVY
TabZIP1	TRIAE_CS42_1AL_TGACv1_000470_AA0012860.1	328	35496.68	5.76	-0.558
TabZIP2	TRIAE_CS42_1AL_TGACv1_000644_AA0016380.1	391	41888.51	6.1	-0.728
TabZIP3	TRIAE_CS42_1AL_TGACv1_000812_AA0019550.1	253	26633.69	6.08	-0.64
TabZIP4	TRIAE_CS42_1AL_TGACv1_001007_AA0023470.1	648	68539.83	8.08	-0.498
TabZIP5	TRIAE_CS42_1AL_TGACv1_001373_AA0029470.1	227	24455.07	5.32	-0.533
TabZIP6.1	TRIAE_CS42_1AL_TGACv1_001653_AA0033650.1	481	53549.34	6.29	-0.512
TabZIP6.2	TRIAE_CS42_1AL_TGACv1_001653_AA0033650.2	445	49684.84	6.68	-0.578
TabZIP6.3	TRIAE_CS42_1AL_TGACv1_001653_AA0033650.3	439	49021.16	6.87	-0.558
TabZIP7.1	TRIAE_CS42_1AL_TGACv1_001758_AA0034810.1	283	30129.97	9.58	-0.695
TabZIP7.2	TRIAE_CS42_1AL_TGACv1_001758_AA0034810.2	382	40303.41	9.29	-0.584
TabZIP8.1	TRIAE_CS42_1AL_TGACv1_003398_AA0049530.1	328	35492.14	7.88	-0.673
TabZIP8.2	TRIAE_CS42_1AL_TGACv1_003398_AA0049530.2	400	43189.18	9.22	-0.505
TabZIP9	TRIAE_CS42_1AS_TGACv1_019004_AA0057730.1	335	36675.16	5.71	-0.438
TabZIP10	TRIAE_CS42_1AS_TGACv1_019984_AA0073590.1	150	16284.28	9.67	-0.672
TabZIP11	TRIAE_CS42_1BL_TGACv1_030508_AA0092770.1	409	44434.9	5.32	-0.458
TabZIP12	TRIAE_CS42_1BL_TGACv1_031237_AA0110100.1	252	26566.6	6.08	-0.646
TabZIP13.1	TRIAE_CS42_1BL_TGACv1_031428_AA0113850.1	439	48970.04	6.08	-0.546
TabZIP13.2	TRIAE_CS42_1BL_TGACv1_031428_AA0113850.2	411	46110.7	6.08	-0.63
TabZIP13.3	TRIAE_CS42_1BL_TGACv1_031428_AA0113850.3	445	49633.72	6.03	-0.567
TabZIP13.4	TRIAE_CS42_1BL_TGACv1_031428_AA0113850.4	481	53484.19	5.81	-0.502
TabZIP13.5	TRIAE_CS42_1BL_TGACv1_031428_AA0113850.5	482	53555.27	5.81	-0.497
TabZIP14	TRIAE_CS42_1BL_TGACv1_031873_AA0122080.1	649	68698.99	7.7	-0.488
TabZIP15	TRIAE_CS42_1BL_TGACv1_032241_AA0127300.1	388	41725.36	6.23	-0.757
TabZIP16.1	TRIAE_CS42_1BL_TGACv1_033059_AA0136530.1	479	50298.91	9.49	-0.482
TabZIP16.2	TRIAE_CS42_1BL_TGACv1_033059_AA0136530.2	383	40373.32	9.22	-0.573
TabZIP17.1	TRIAE_CS42_1BS_TGACv1_049763_AA0161060.1	340	37258.1	6.14	-0.357
TabZIP17.2	TRIAE_CS42_1BS_TGACv1_049763_AA0161060.2	334	36635.29	6.14	-0.409
TabZIP17.3	TRIAE_CS42_1BS_TGACv1_049763_AA0161060.3	333	36564.21	6.14	-0.416
TabZIP18	TRIAE_CS42_1DL_TGACv1_061220_AA0189160.1	403	43702.07	5.76	-0.482
TabZIP19.1	TRIAE_CS42_1DL_TGACv1_061988_AA0206970.1	252	26548.59	6.08	-0.658
TabZIP19.2	TRIAE_CS42_1DL_TGACv1_061988_AA0206970.2	235	25094.03	7.99	-0.674
TabZIP19.3	TRIAE_CS42_1DL_TGACv1_061988_AA0206970.3	226	23350.1	5.43	-0.406
TabZIP20.1	TRIAE_CS42_1DL_TGACv1_062019_AA0207490.1	482	53617.37	5.95	-0.498
TabZIP20.2	TRIAE_CS42_1DL_TGACv1_062019_AA0207490.2	424	47433.49	6.24	-0.512
TabZIP21	TRIAE_CS42_1DL_TGACv1_062780_AA0219780.1	652	68897.15	7.3	-0.487
TabZIP22	TRIAE_CS42_1DL_TGACv1_063151_AA0224440.1	386	41402.99	6.23	-0.738
TabZIP23	TRIAE_CS42_1DS_TGACv1_080292_AA0245420.1	338	37042.89	6.56	-0.371

TabZIP24	TRIAE_CS42_1DS_TGACv1_081606_AA0261850.1	150	16197.15	9.38	-0.617
TabZIP25	TRIAE_CS42_2AL_TGACv1_093386_AA0278960.1	200	22201.88	10.12	-0.887
TabZIP26	TRIAE_CS42_2AL_TGACv1_093662_AA0284750.1	178	19555.84	9.29	-0.814
TabZIP27	TRIAE_CS42_2AL_TGACv1_093662_AA0284760.1	225	24441.77	9.76	-0.562
TabZIP28	TRIAE_CS42_2AL_TGACv1_094886_AA0304600.1	131	14323.04	10.08	-0.792
TabZIP29.1	TRIAE_CS42_2AL_TGACv1_095790_AA0314590.1	373	39905.12	5.76	-0.755
TabZIP29.2	TRIAE_CS42_2AL_TGACv1_095790_AA0314590.2	316	34126.2	9.71	-0.7
TabZIP30.1	TRIAE_CS42_2AL_TGACv1_096383_AA0318980.1	419	46270.21	6.24	-0.536
TabZIP30.2	TRIAE_CS42_2AL_TGACv1_096383_AA0318980.2	391	43164.78	6.61	-0.528
TabZIP31.1	TRIAE_CS42_2AL_TGACv1_096691_AA0320620.1	378	39318.44	6.21	-0.642
TabZIP31.2	TRIAE_CS42_2AL_TGACv1_096691_AA0320620.2	377	39190.31	6.21	-0.635
TabZIP31.3	TRIAE_CS42_2AL_TGACv1_096691_AA0320620.4	313	32191.66	7.64	-0.528
TabZIP31.4	TRIAE_CS42_2AL_TGACv1_096691_AA0320620.5	313	32379.09	7.64	-0.392
TabZIP32	TRIAE_CS42_2AS_TGACv1_113477_AA0356620.1	643	68355.5	6.84	-0.319
TabZIP33	TRIAE_CS42_2AS_TGACv1_113693_AA0359510.1	274	29836.68	9.48	-0.695
TabZIP34.1	TRIAE_CS42_2AS_TGACv1_114933_AA0370000.1	368	39688.26	6.94	-0.782
TabZIP34.2	TRIAE_CS42_2AS_TGACv1_114933_AA0370000.2	331	35443.99	9.34	-0.678
TabZIP34.3	TRIAE_CS42_2AS_TGACv1_114933_AA0370000.3	363	39123.71	7.27	-0.777
TabZIP35	TRIAE_CS42_2AS_TGACv1_115473_AA0372450.1	306	33241.7	6.11	-0.755
TabZIP36	TRIAE_CS42_2BL_TGACv1_129531_AA0387390.1	382	41030.36	5.68	-0.805
TabZIP37	TRIAE_CS42_2BL_TGACv1_129714_AA0393610.1	134	14717.43	9.91	-0.839
TabZIP38.1	TRIAE_CS42_2BL_TGACv1_130015_AA0401630.1	435	48378.54	6.23	-0.565
TabZIP38.2	TRIAE_CS42_2BL_TGACv1_130015_AA0401630.3	321	35697.67	7.86	-0.406
TabZIP38.3	TRIAE_CS42_2BL_TGACv1_130015_AA0401630.5	407	45273.11	6.47	-0.56
TabZIP38.4	TRIAE_CS42_2BL_TGACv1_130015_AA0401630.8	298	33398.53	7.28	-0.723
TabZIP39	TRIAE_CS42_2BL_TGACv1_130715_AA0416520.1	138	14563.35	8.82	-0.58
TabZIP40	TRIAE_CS42_2BL_TGACv1_130715_AA0416530.1	177	19312.43	8.57	-0.77
TabZIP41	TRIAE_CS42_2BL_TGACv1_133905_AA0443370.1	183	20415.16	10.14	-0.801
TabZIP42.1	TRIAE_CS42_2BS_TGACv1_146410_AA0464450.1	430	45322.12	5.23	-0.39
TabZIP42.2	TRIAE_CS42_2BS_TGACv1_146410_AA0464450.2	570	60486.4	6.2	-0.345
TabZIP42.3	TRIAE_CS42_2BS_TGACv1_146410_AA0464450.3	441	46604.58	5.32	-0.396
TabZIP43.1	TRIAE_CS42_2BS_TGACv1_146702_AA0471100.1	370	39987.71	6.94	-0.777
TabZIP43.2	TRIAE_CS42_2BS_TGACv1_146702_AA0471100.2	466	51173.61	9.07	-0.801
TabZIP44	TRIAE_CS42_2BS_TGACv1_147553_AA0484820.1	279	30082.95	9.48	-0.653
TabZIP45	TRIAE_CS42_2BS_TGACv1_147765_AA0487460.1	307	33353	6.93	-0.74
TabZIP46	TRIAE_CS42_2DL_TGACv1_159083_AA0532000.1	196	21207.76	9.5	-0.773
TabZIP47	TRIAE_CS42_2DL_TGACv1_159083_AA0532010.1	200	21702.2	9.54	-0.71
TabZIP48.1	TRIAE_CS42_2DL_TGACv1_159174_AA0533780.1	129	14098.74	10.08	-0.826
TabZIP48.2	TRIAE_CS42_2DL_TGACv1_159174_AA0533780.2	131	14340.03	10.08	-0.811
TabZIP49	TRIAE_CS42_2DL_TGACv1_159820_AA0543160.1	200	22227.88	10.2	-0.919
TabZIP50.1	TRIAE_CS42_2DL_TGACv1_159977_AA0545060.1	425	46843.83	5.86	-0.507
TabZIP50.2	TRIAE_CS42_2DL_TGACv1_159977_AA0545060.4	320	35426.23	7.85	-0.418
TabZIP50.3	TRIAE_CS42_2DL_TGACv1_159977_AA0545060.5	397	43738.41	6.11	-0.497

TabZIP51	TRIAE_CS42_2DL_TGACv1_161713_AA0560210.1	383	41259.49	5.81	-0.836
TabZIP52	TRIAE_CS42_2DS_TGACv1_177487_AA0578420.1	572	60589.43	6.07	-0.342
TabZIP53	TRIAE_CS42_2DS_TGACv1_177541_AA0579660.1	272	29506.38	9.45	-0.621
TabZIP54.1	TRIAE_CS42_2DS_TGACv1_178263_AA0593550.1	299	30962.02	5.65	-0.683
TabZIP54.2	TRIAE_CS42_2DS_TGACv1_178263_AA0593550.2	377	39246.39	6.28	-0.649
TabZIP54.3	TRIAE_CS42_2DS_TGACv1_178263_AA0593550.3	378	39374.52	6.28	-0.657
TabZIP54.4	TRIAE_CS42_2DS_TGACv1_178263_AA0593550.4	379	39502.65	6.28	-0.664
TabZIP54.5	TRIAE_CS42_2DS_TGACv1_178263_AA0593550.5	378	39374.52	6.28	-0.657
TabZIP54.6	TRIAE_CS42_2DS_TGACv1_178263_AA0593550.6	323	33644.55	8.5	-0.433
TabZIP55	TRIAE_CS42_2DS_TGACv1_178412_AA0595200.1	306	33323.94	6.35	-0.745
TabZIP56.1	TRIAE_CS42_2DS_TGACv1_178575_AA0597660.1	411	44577.18	7.91	-0.574
TabZIP56.2	TRIAE_CS42_2DS_TGACv1_178575_AA0597660.2	368	39657.25	6.94	-0.772
TabZIP56.3	TRIAE_CS42_2DS_TGACv1_178575_AA0597660.3	363	39092.7	7.27	-0.766
TabZIP57	TRIAE_CS42_3AL_TGACv1_193561_AA0613050.1	391	41802.42	5.83	-0.488
TabZIP58	TRIAE_CS42_3AL_TGACv1_193880_AA0621650.1	154	16302.37	7.66	-0.268
TabZIP59.1	TRIAE_CS42_3AL_TGACv1_194119_AA0626840.1	524	56876.86	6.86	-0.535
TabZIP59.2	TRIAE_CS42_3AL_TGACv1_194119_AA0626840.2	447	49191.43	6.95	-0.543
TabZIP59.3	TRIAE_CS42_3AL_TGACv1_194119_AA0626840.3	527	57231.37	7.06	-0.529
TabZIP59.4	TRIAE_CS42_3AL_TGACv1_194119_AA0626840.4	573	62142.74	6.64	-0.511
TabZIP59.5	TRIAE_CS42_3AL_TGACv1_194119_AA0626840.5	570	61788.23	6.55	-0.516
TabZIP59.6	TRIAE_CS42_3AL_TGACv1_194119_AA0626840.7	520	55898.45	6.22	-0.48
TabZIP60	TRIAE_CS42_3AL_TGACv1_194206_AA0628450.1	475	51766.93	6.53	-0.514
TabZIP61	TRIAE_CS42_3AL_TGACv1_194214_AA0628790.1	263	28680.75	6.06	-0.8
TabZIP62	TRIAE_CS42_3AL_TGACv1_194390_AA0632360.1	383	41494.95	5.67	-0.556
TabZIP63	TRIAE_CS42_3AL_TGACv1_195373_AA0648710.1	382	41859.52	5.54	-0.526
TabZIP64	TRIAE_CS42_3AL_TGACv1_195666_AA0652540.1	338	36726.31	9.26	-0.66
TabZIP65	TRIAE_CS42_3AL_TGACv1_196209_AA0658260.1	214	23115.99	5.7	-0.272
TabZIP66	TRIAE_CS42_3AL_TGACv1_197019_AA0664400.1	391	42286.95	5.79	-0.501
TabZIP67	TRIAE_CS42_3AL_TGACv1_197036_AA0664480.1	257	27314.57	7.99	-0.751
TabZIP68.1	TRIAE_CS42_3AS_TGACv1_211031_AA0683480.1	332	36924.46	7.79	-0.605
TabZIP68.2	TRIAE_CS42_3AS_TGACv1_211031_AA0683480.2	284	32749.97	9.33	-0.731
TabZIP69.1	TRIAE_CS42_3AS_TGACv1_211403_AA0689810.1	152	16205.24	9.88	-0.975
TabZIP69.2	TRIAE_CS42_3AS_TGACv1_211403_AA0689810.2	178	18827.87	10.11	-1.185
TabZIP70	TRIAE_CS42_3AS_TGACv1_211574_AA0691710.1	306	33082.4	5.86	-0.762
TabZIP71	TRIAE_CS42_3B_TGACv1_220594_AA0710270.1	391	41795.44	5.39	-0.459
TabZIP72	TRIAE_CS42_3B_TGACv1_220594_AA0710310.1	380	41593.35	5.62	-0.546
TabZIP73	TRIAE_CS42_3B_TGACv1_220594_AA0710320.1	390	41868.53	6.23	-0.487
TabZIP74	TRIAE_CS42_3B_TGACv1_220944_AA0724530.1	387	41658.52	7.03	-0.486
TabZIP75	TRIAE_CS42_3B_TGACv1_220946_AA0724560.1	343	37353.08	9.42	-0.671
TabZIP76	TRIAE_CS42_3B_TGACv1_221145_AA0731790.1	149	15947.14	8.41	-0.248
TabZIP77.1	TRIAE_CS42_3B_TGACv1_221472_AA0741680.1	523	56175.79	6.13	-0.459
TabZIP77.2	TRIAE_CS42_3B_TGACv1_221472_AA0741680.2	570	61710.07	6.39	-0.502
TabZIP77.3	TRIAE_CS42_3B_TGACv1_221472_AA0741680.3	596	64289.96	6.57	-0.524

TabZIP77.4	TRIAE_CS42_3B_TGACv1_221472_AA0741680.4	573	62064.58	6.45	-0.497
TabZIP77.5	TRIAE_CS42_3B_TGACv1_221472_AA0741680.5	541	58507.75	6.56	-0.468
TabZIP77.6	TRIAE_CS42_3B_TGACv1_221472_AA0741680.6	557	60270.53	6.19	-0.458
TabZIP77.7	TRIAE_CS42_3B_TGACv1_221472_AA0741680.7	520	55821.28	6.08	-0.464
TabZIP77.8	TRIAE_CS42_3B_TGACv1_221472_AA0741680.8	466	50898.14	6.82	-0.573
TabZIP78	TRIAE_CS42_3B_TGACv1_221698_AA0747790.1	250	26792.04	8.01	-0.76
TabZIP79.1	TRIAE_CS42_3B_TGACv1_221777_AA0749730.1	213	22947.83	5.6	-0.191
TabZIP79.2	TRIAE_CS42_3B_TGACv1_221777_AA0749730.2	253	27398.06	5.69	-0.049
TabZIP80.1	TRIAE_CS42_3B_TGACv1_222340_AA0762500.1	158	16601.22	10.14	-1.26
TabZIP80.2	TRIAE_CS42_3B_TGACv1_222340_AA0762500.2	179	18995.98	9.99	-1.196
TabZIP80.3	TRIAE_CS42_3B_TGACv1_222340_AA0762500.3	165	17386.13	10.18	-1.233
TabZIP81	TRIAE_CS42_3B_TGACv1_222491_AA0765820.2	325	36098.58	7.79	-0.578
TabZIP82	TRIAE_CS42_3B_TGACv1_223228_AA0778460.1	477	51918.14	6.78	-0.494
TabZIP83	TRIAE_CS42_3B_TGACv1_225111_AA0805030.2	268	29087.14	5.88	-0.777
TabZIP84.1	TRIAE_CS42_3B_TGACv1_227947_AA0825230.1	389	41612.13	5.28	-0.453
TabZIP84.2	TRIAE_CS42_3B_TGACv1_227947_AA0825230.2	383	41016.46	5.16	-0.368
TabZIP85	TRIAE_CS42_3DL_TGACv1_249032_AA0835230.1	154	16293.39	8.52	-0.273
TabZIP86	TRIAE_CS42_3DL_TGACv1_249583_AA0851920.1	390	41799.47	5.9	-0.488
TabZIP87	TRIAE_CS42_3DL_TGACv1_249704_AA0854830.1	396	42724.46	6.12	-0.471
TabZIP88	TRIAE_CS42_3DL_TGACv1_250203_AA0864170.1	477	51979.22	6.53	-0.495
TabZIP89	TRIAE_CS42_3DL_TGACv1_251113_AA0877600.1	214	22983.92	5.49	-0.157
TabZIP90.1	TRIAE_CS42_3DL_TGACv1_251145_AA0878040.1	337	36528.07	9.37	-0.651
TabZIP90.2	TRIAE_CS42_3DL_TGACv1_251145_AA0878040.2	349	38190.89	9.51	-0.676
TabZIP91.1	TRIAE_CS42_3DL_TGACv1_251522_AA0882230.1	531	57212.22	6.15	-0.452
TabZIP91.2	TRIAE_CS42_3DL_TGACv1_251522_AA0882230.2	577	62563.95	6.47	-0.527
TabZIP91.3	TRIAE_CS42_3DL_TGACv1_251522_AA0882230.3	580	62918.46	6.54	-0.522
TabZIP91.4	TRIAE_CS42_3DL_TGACv1_251522_AA0882230.5	530	57555.46	6.66	-0.545
TabZIP91.5	TRIAE_CS42_3DL_TGACv1_251522_AA0882230.6	533	57909.97	6.79	-0.539
TabZIP92	TRIAE_CS42_3DL_TGACv1_253126_AA0893500.1	268	29045.06	5.88	-0.786
TabZIP93.1	TRIAE_CS42_3DS_TGACv1_273046_AA0927740.1	305	32929.42	6.34	-0.728
TabZIP93.2	TRIAE_CS42_3DS_TGACv1_273046_AA0927740.2	339	36986.19	8.27	-0.793
TabZIP94	TRIAE_CS42_3DS_TGACv1_273334_AA0930450.1	129	13641.34	10.5	-1.119
TabZIP95.1	TRIAE_CS42_3DS_TGACv1_274210_AA0935070.1	327	36386.85	8.49	-0.626
TabZIP95.2	TRIAE_CS42_3DS_TGACv1_274210_AA0935070.2	332	36929.48	8.49	-0.609
TabZIP96.1	TRIAE_CS42_4AL_TGACv1_288889_AA0960680.1	580	64497.31	9.5	-0.816
TabZIP96.2	TRIAE_CS42_4AL_TGACv1_288889_AA0960680.2	581	64584.39	9.5	-0.816
TabZIP96.3	TRIAE_CS42_4AL_TGACv1_288889_AA0960680.3	503	55773.35	9.83	-0.886
TabZIP97	TRIAE_CS42_4AL_TGACv1_289134_AA0965530.1	331	35408.21	6.6	-0.776
TabZIP98	TRIAE_CS42_4AL_TGACv1_290310_AA0983850.1	398	41762.94	8.87	-0.573
TabZIP99	TRIAE_CS42_4AL_TGACv1_292709_AA0999550.1	164	17494.63	10.53	-0.303
TabZIP100	TRIAE_CS42_4AS_TGACv1_306303_AA1006020.1	356	38478.75	6.43	-0.669
TabZIP101.1	TRIAE_CS42_4AS_TGACv1_307687_AA1022710.1	381	40728.05	9.25	-0.981
TabZIP101.2	TRIAE_CS42_4AS_TGACv1_307687_AA1022710.2	348	37504.01	9.63	-0.888

TabZIP102	TRIAE_CS42_4AS_TGACv1_308096_AA1025750.1	167	18815.58	6.12	-0.525
TabZIP103	TRIAE_CS42_4AS_TGACv1_308354_AA1027470.1	225	24600.48	9.68	-0.869
TabZIP104	TRIAE_CS42_4BL_TGACv1_320329_AA1035550.2	356	38399.61	6.32	-0.685
TabZIP105	TRIAE_CS42_4BL_TGACv1_320366_AA1036870.1	201	21838.31	9.66	-0.741
TabZIP106.1	TRIAE_CS42_4BL_TGACv1_320443_AA1039420.1	920	103217.27	5.6	-0.359
TabZIP106.2	TRIAE_CS42_4BL_TGACv1_320443_AA1039420.2	334	37268.18	7.87	-0.513
TabZIP107	TRIAE_CS42_4BL_TGACv1_320589_AA1043990.1	167	18755.25	5.89	-0.669
TabZIP108	TRIAE_CS42_4BL_TGACv1_320592_AA1044030.1	381	40759.12	9.17	-0.985
TabZIP109	TRIAE_CS42_4BL_TGACv1_320759_AA1048010.1	400	42666.48	6.03	-0.579
TabZIP110	TRIAE_CS42_4BL_TGACv1_321014_AA1053680.1	225	24641.45	9.68	-0.92
TabZIP111	TRIAE_CS42_4BS_TGACv1_328709_AA1092270.1	329	35232.94	6.46	-0.812
TabZIP112	TRIAE_CS42_4DL_TGACv1_342413_AA1113000.1	167	18699.4	6.59	-0.484
TabZIP113	TRIAE_CS42_4DL_TGACv1_342950_AA1125920.1	356	38404.63	6.43	-0.695
TabZIP114	TRIAE_CS42_4DL_TGACv1_343105_AA1129830.1	381	40683	9.17	-0.962
TabZIP115	TRIAE_CS42_4DL_TGACv1_343148_AA1130650.1	203	22283.79	10.01	-0.778
TabZIP116	TRIAE_CS42_4DL_TGACv1_343219_AA1131620.1	393	41835.63	6.17	-0.536
TabZIP117.1	TRIAE_CS42_4DL_TGACv1_343394_AA1133900.1	334	37250.15	7.87	-0.505
TabZIP117.2	TRIAE_CS42_4DL_TGACv1_343394_AA1133900.2	335	37349.28	7.87	-0.491
TabZIP117.3	TRIAE_CS42_4DL_TGACv1_343394_AA1133900.6	377	41910.73	8.97	-0.459
TabZIP118	TRIAE_CS42_4DL_TGACv1_344938_AA1150590.1	223	24518.38	9.93	-0.919
TabZIP119	TRIAE_CS42_4DS_TGACv1_361639_AA1171000.1	330	35309.08	6.41	-0.792
TabZIP120.1	TRIAE_CS42_4DS_TGACv1_361940_AA1174570.1	540	59059.01	6.41	-0.559
TabZIP120.2	TRIAE_CS42_4DS_TGACv1_361940_AA1174570.2	541	59187.14	6.41	-0.565
TabZIP120.3	TRIAE_CS42_4DS_TGACv1_361940_AA1174570.3	539	58971.93	6.41	-0.559
TabZIP121	TRIAE_CS42_4DS_TGACv1_362363_AA1179150.1	496	52160.12	9.41	-0.512
TabZIP122	TRIAE_CS42_5AL_TGACv1_374182_AA1192680.1	345	undefined	undefined	-0.539
TabZIP123	TRIAE_CS42_5AL_TGACv1_374195_AA1193190.1	518	57856.21	6.51	-0.55
TabZIP124	TRIAE_CS42_5AL_TGACv1_374263_AA1195450.1	239	26051.39	6	-0.447
TabZIP125	TRIAE_CS42_5AL_TGACv1_374308_AA1196510.1	196	21828.26	6.87	-0.883
TabZIP126	TRIAE_CS42_5AL_TGACv1_374428_AA1199910.1	467	51251.99	7.02	-0.473
TabZIP127	TRIAE_CS42_5AL_TGACv1_374464_AA1200910.1	380	39784.33	6.42	-0.611
TabZIP128	TRIAE_CS42_5AL_TGACv1_374468_AA1200980.1	204	21238.9	9.85	-0.364
TabZIP129.1	TRIAE_CS42_5AL_TGACv1_374503_AA1201830.1	374	39869.42	6.87	-0.31
TabZIP129.2	TRIAE_CS42_5AL_TGACv1_374503_AA1201830.2	367	39271.48	8.59	-0.532
TabZIP129.3	TRIAE_CS42_5AL_TGACv1_374503_AA1201830.3	368	39399.62	8.59	-0.54
TabZIP130.1	TRIAE_CS42_5AL_TGACv1_375037_AA1214490.1	500	53323.82	9.56	-0.552
TabZIP130.2	TRIAE_CS42_5AL_TGACv1_375037_AA1214490.2	464	49117.55	10.04	-0.427
TabZIP131	TRIAE_CS42_5AL_TGACv1_375049_AA1214770.1	332	36076.02	6.84	-0.801
TabZIP132.1	TRIAE_CS42_5AL_TGACv1_375281_AA1219050.1	313	34883.24	6.4	-0.689
TabZIP132.2	TRIAE_CS42_5AL_TGACv1_375281_AA1219050.3	275	30682.54	9.16	-0.773
TabZIP132.3	TRIAE_CS42_5AL_TGACv1_375281_AA1219050.4	310	34776.17	5.94	-0.537
TabZIP133	TRIAE_CS42_5AL_TGACv1_375448_AA1221780.1	183	19986.82	11.38	-0.562
TabZIP134	TRIAE_CS42_5AL_TGACv1_375720_AA1226160.1	206	22376.92	10.13	-0.739

TabZIP135	TRIAE_CS42_5AL_TGACv1_375799_AA1227320.1	392	41249.82	5.06	-0.509
TabZIP136.1	TRIAE_CS42_5AL_TGACv1_376455_AA1237180.1	300	31062.25	9.11	-0.777
TabZIP136.2	TRIAE_CS42_5AL_TGACv1_376455_AA1237180.2	356	36698.53	8.89	-0.683
TabZIP137	TRIAE_CS42_5AL_TGACv1_376758_AA1240660.1	377	41096.86	6.79	-0.842
TabZIP138	TRIAE_CS42_5AL_TGACv1_376957_AA1242740.1	154	17166.37	9.16	-0.632
TabZIP139.1	TRIAE_CS42_5AS_TGACv1_393100_AA1268400.1	308	33191.28	5.79	-0.428
TabZIP139.2	TRIAE_CS42_5AS_TGACv1_393100_AA1268400.2	268	28432.83	5.55	-0.443
TabZIP140	TRIAE_CS42_5AS_TGACv1_394198_AA1278690.1	183	20202.58	6.61	-0.804
TabZIP141	TRIAE_CS42_5BL_TGACv1_404292_AA1294130.1	193	21611.08	6.87	-0.884
TabZIP142.1	TRIAE_CS42_5BL_TGACv1_404323_AA1295530.1	402	43791.87	9.35	-0.371
TabZIP142.2	TRIAE_CS42_5BL_TGACv1_404323_AA1295530.2	456	49832.69	8.53	-0.339
TabZIP143	TRIAE_CS42_5BL_TGACv1_404455_AA1300380.1	313	34887.28	6.4	-0.693
TabZIP144	TRIAE_CS42_5BL_TGACv1_404662_AA1307660.1	186	20497.36	11.25	-0.593
TabZIP145.1	TRIAE_CS42_5BL_TGACv1_405507_AA1329020.1	196	21126.67	5.41	-0.421
TabZIP145.2	TRIAE_CS42_5BL_TGACv1_405507_AA1329020.2	239	26047.4	6	-0.453
TabZIP145.3	TRIAE_CS42_5BL_TGACv1_405507_AA1329020.3	153	16414.33	4.91	-0.382
TabZIP146	TRIAE_CS42_5BL_TGACv1_405777_AA1335110.1	411	46110.7	6.08	-0.63
TabZIP147.1	TRIAE_CS42_5BL_TGACv1_405990_AA1338700.1	356	38659.13	6.68	-0.816
TabZIP147.2	TRIAE_CS42_5BL_TGACv1_405990_AA1338700.2	377	41097.9	6.74	-0.837
TabZIP148	TRIAE_CS42_5BL_TGACv1_406586_AA1347450.1	441	46130.64	6.81	-0.527
TabZIP149	TRIAE_CS42_5BL_TGACv1_406690_AA1348690.1	199	20850.47	9.85	-0.376
TabZIP150	TRIAE_CS42_5BL_TGACv1_407412_AA1356630.1	509	54270.45	10.17	-0.55
TabZIP151	TRIAE_CS42_5BL_TGACv1_408138_AA1361800.1	400	41946.62	5.12	-0.472
TabZIP152	TRIAE_CS42_5BL_TGACv1_408841_AA1364640.1	525	58620.02	6.58	-0.56
TabZIP153	TRIAE_CS42_5BS_TGACv1_423537_AA1378880.1	157	17410.53	9.02	-0.635
TabZIP154	TRIAE_CS42_5BS_TGACv1_423566_AA1379610.1	265	28098.53	5.83	-0.448
TabZIP155.1	TRIAE_CS42_5BS_TGACv1_424687_AA1391330.1	298	32551.2	5.41	-0.667
TabZIP155.2	TRIAE_CS42_5BS_TGACv1_424687_AA1391330.2	187	20607.88	5.96	-0.876
TabZIP155.3	TRIAE_CS42_5BS_TGACv1_424687_AA1391330.4	183	20229.59	6.61	-0.815
TabZIP156	TRIAE_CS42_5DL_TGACv1_432931_AA1394910.1	252	26884.45	9.5	-0.537
TabZIP157.1	TRIAE_CS42_5DL_TGACv1_433182_AA1404910.1	376	40903.71	6.68	-0.838
TabZIP157.2	TRIAE_CS42_5DL_TGACv1_433182_AA1404910.2	340	36990.27	7.86	-0.862
TabZIP157.3	TRIAE_CS42_5DL_TGACv1_433182_AA1404910.3	359	39228.85	8.48	-0.881
TabZIP157.4	TRIAE_CS42_5DL_TGACv1_433182_AA1404910.4	238	25545.77	6	-0.525
TabZIP158	TRIAE_CS42_5DL_TGACv1_433372_AA1411290.1	406	42383.03	5.14	-0.488
TabZIP159.1	TRIAE_CS42_5DL_TGACv1_433373_AA1411370.1	316	35177.63	5.8	-0.532
TabZIP159.2	TRIAE_CS42_5DL_TGACv1_433373_AA1411370.2	313	34882.21	6.63	-0.704
TabZIP160	TRIAE_CS42_5DL_TGACv1_433913_AA1425130.1	154	17093.24	9.16	-0.649
TabZIP161	TRIAE_CS42_5DL_TGACv1_434170_AA1430970.1	239	26001.37	6.18	-0.454
TabZIP162	TRIAE_CS42_5DL_TGACv1_434504_AA1437070.1	316	33650.81	6.67	-1.055
TabZIP163	TRIAE_CS42_5DL_TGACv1_434744_AA1440910.1	332	36031	6.87	-0.794
TabZIP164	TRIAE_CS42_5DL_TGACv1_434960_AA1444170.1	437	48988.51	7.44	-0.514
TabZIP165	TRIAE_CS42_5DL_TGACv1_435011_AA1444870.1	199	20947.63	9.85	-0.389

TabZIP166	TRIAE_CS42_5DL_TGACv1_435031_AA1445050.1	187	20615.53	11.34	-0.556
TabZIP167.1	TRIAE_CS42_5DL_TGACv1_435093_AA1445920.1	466	51046.77	7.83	-0.466
TabZIP167.2	TRIAE_CS42_5DL_TGACv1_435093_AA1445920.3	423	46332.57	8.83	-0.417
TabZIP168	TRIAE_CS42_5DL_TGACv1_435926_AA1456400.1	193	21582.02	6.45	-0.858
TabZIP169	TRIAE_CS42_5DL_TGACv1_436292_AA1459750.1	509	54203.3	9.98	-0.567
TabZIP170.1	TRIAE_CS42_5DL_TGACv1_436729_AA1462940.1	366	39291.56	7.79	-0.542
TabZIP170.2	TRIAE_CS42_5DL_TGACv1_436729_AA1462940.2	365	39163.43	7.79	-0.534
TabZIP171.1	TRIAE_CS42_5DL_TGACv1_437065_AA1464880.1	255	26626.33	9.18	-0.942
TabZIP171.2	TRIAE_CS42_5DL_TGACv1_437065_AA1464880.2	362	37053.76	8.7	-0.709
TabZIP172.1	TRIAE_CS42_5DS_TGACv1_456478_AA1471590.1	183	20188.55	6.61	-0.816
TabZIP172.2	TRIAE_CS42_5DS_TGACv1_456478_AA1471590.2	186	20547.97	6.61	-0.798
TabZIP172.3	TRIAE_CS42_5DS_TGACv1_456478_AA1471590.3	187	20626.94	5.97	-0.858
TabZIP172.4	TRIAE_CS42_5DS_TGACv1_456478_AA1471590.4	298	32535.22	5.32	-0.641
TabZIP173.1	TRIAE_CS42_5DS_TGACv1_456500_AA1472280.1	266	28266.59	5.43	-0.521
TabZIP173.2	TRIAE_CS42_5DS_TGACv1_456500_AA1472280.2	233	24619.46	5.68	-0.507
TabZIP174	TRIAE_CS42_5DS_TGACv1_457365_AA1485570.1	157	17413.53	9.02	-0.634
TabZIP175.1	TRIAE_CS42_6AL_TGACv1_471610_AA1511740.1	350	37817.94	9.7	-0.641
TabZIP175.2	TRIAE_CS42_6AL_TGACv1_471610_AA1511740.2	369	39923.43	9.8	-0.631
TabZIP175.3	TRIAE_CS42_6AL_TGACv1_471610_AA1511740.3	351	38004.15	9.7	-0.642
TabZIP176	TRIAE_CS42_6AL_TGACv1_474358_AA1535080.1	206	22578.39	6.76	-0.474
TabZIP177	TRIAE_CS42_6AS_TGACv1_485564_AA1547640.1	458	49997.3	6.96	-0.457
TabZIP178.1	TRIAE_CS42_6AS_TGACv1_486123_AA1557200.1	323	34205.43	5.76	-1.026
TabZIP178.2	TRIAE_CS42_6AS_TGACv1_486123_AA1557200.2	344	36367.84	6.02	-0.994
TabZIP178.3	TRIAE_CS42_6AS_TGACv1_486123_AA1557200.3	347	36624.1	6.02	-0.992
TabZIP179	TRIAE_CS42_6AS_TGACv1_486251_AA1558790.1	177	19139.12	9.01	-0.799
TabZIP180	TRIAE_CS42_6AS_TGACv1_487131_AA1568440.1	174	18258.59	8.02	-0.115
TabZIP181	TRIAE_CS42_6AS_TGACv1_488428_AA1575500.1	313	32930.58	5.42	-0.386
TabZIP182	TRIAE_CS42_6BL_TGACv1_500317_AA1603110.1	207	22988.73	6.5	-0.533
TabZIP183.1	TRIAE_CS42_6BL_TGACv1_500899_AA1611470.1	351	37854.17	9.6	-0.575
TabZIP183.2	TRIAE_CS42_6BL_TGACv1_500899_AA1611470.2	352	38040.39	9.6	-0.576
TabZIP184.1	TRIAE_CS42_6BS_TGACv1_513324_AA1638290.1	457	49907.18	6.98	-0.455
TabZIP184.2	TRIAE_CS42_6BS_TGACv1_513324_AA1638290.2	458	50063.37	7.3	-0.464
TabZIP185	TRIAE_CS42_6BS_TGACv1_513373_AA1639310.1	175	18340.7	8.04	-0.109
TabZIP186	TRIAE_CS42_6BS_TGACv1_513423_AA1641170.1	156	17292.98	9.83	-1.413
TabZIP187	TRIAE_CS42_6BS_TGACv1_514351_AA1658680.1	177	19280.19	6.92	-0.881
TabZIP188.1	TRIAE_CS42_6BS_TGACv1_515508_AA1670570.1	304	32031.52	5.42	-0.425
TabZIP188.2	TRIAE_CS42_6BS_TGACv1_515508_AA1670570.2	311	32759.34	5.42	-0.417
TabZIP188.3	TRIAE_CS42_6BS_TGACv1_515508_AA1670570.3	305	32102.6	5.42	-0.418
TabZIP188.4	TRIAE_CS42_6BS_TGACv1_515508_AA1670570.4	312	32830.42	5.42	-0.41
TabZIP188.5	TRIAE_CS42_6BS_TGACv1_515508_AA1670570.5	302	32693.68	6.71	-0.375
TabZIP188.6	TRIAE_CS42_6BS_TGACv1_515508_AA1670570.6	240	25405.21	5.11	-0.39
TabZIP189.1	TRIAE_CS42_6BS_TGACv1_515656_AA1671610.1	346	36486.96	5.96	-0.979
TabZIP189.2	TRIAE_CS42_6BS_TGACv1_515656_AA1671610.2	325	34324.55	5.69	-1.01

TabZIP189.3	TRIAE_CS42_6BS_TGACv1_515656_AA1671610.3	349	36743.22	5.96	-0.977
TabZIP190.1	TRIAE_CS42_6DL_TGACv1_526318_AA1679380.1	360	38670.06	9.7	-0.588
TabZIP190.2	TRIAE_CS42_6DL_TGACv1_526318_AA1679380.2	361	38856.27	9.7	-0.588
TabZIP190.3	TRIAE_CS42_6DL_TGACv1_526318_AA1679380.3	379	40981.77	7.77	-0.406
TabZIP191	TRIAE_CS42_6DL_TGACv1_527360_AA1702780.1	199	22348.97	6.5	-0.605
TabZIP192	TRIAE_CS42_6DS_TGACv1_542930_AA1732700.1	174	18200.51	8.04	-0.103
TabZIP193.1	TRIAE_CS42_6DS_TGACv1_545295_AA1750190.1	313	32960.57	5.42	-0.395
TabZIP193.2	TRIAE_CS42_6DS_TGACv1_545295_AA1750190.2	233	24749.52	5.11	-0.406
TabZIP193.3	TRIAE_CS42_6DS_TGACv1_545295_AA1750190.3	312	32832.43	5.42	-0.385
TabZIP193.4	TRIAE_CS42_6DS_TGACv1_545295_AA1750190.4	171	18283.53	5.78	-0.358
TabZIP194.1	TRIAE_CS42_7AL_TGACv1_556285_AA1759970.1	250	27051.14	7.02	-0.676
TabZIP194.2	TRIAE_CS42_7AL_TGACv1_556285_AA1759970.2	251	27179.27	7.02	-0.688
TabZIP194.3	TRIAE_CS42_7AL_TGACv1_556285_AA1759970.3	193	20835.01	6.39	-0.735
TabZIP195	TRIAE_CS42_7AL_TGACv1_556796_AA1770930.1	164	17551.16	9.97	-1.124
TabZIP196	TRIAE_CS42_7AL_TGACv1_557050_AA1775820.1	242	26055.32	10.13	-0.394
TabZIP197	TRIAE_CS42_7AL_TGACv1_557464_AA1781650.1	164	17975.3	9.26	-0.636
TabZIP198.1	TRIAE_CS42_7AL_TGACv1_558306_AA1792200.1	302	32643.65	4.65	-0.434
TabZIP198.2	TRIAE_CS42_7AL_TGACv1_558306_AA1792200.2	253	27659.58	5.15	-0.34
TabZIP199	TRIAE_CS42_7AL_TGACv1_558616_AA1794710.1	306	34329.25	6.99	-0.938
TabZIP200	TRIAE_CS42_7AS_TGACv1_569208_AA1810370.1	217	23382.98	6.76	-0.628
TabZIP201	TRIAE_CS42_7AS_TGACv1_569238_AA1811090.1	467	50198.56	6.61	-0.842
TabZIP202	TRIAE_CS42_7AS_TGACv1_569350_AA1814160.1	185	20930.42	5.97	-0.696
TabZIP203	TRIAE_CS42_7AS_TGACv1_569625_AA1820550.1	365	39384.33	6.49	-0.633
TabZIP204.1	TRIAE_CS42_7AS_TGACv1_571414_AA1847500.1	343	36979.25	8.5	-0.399
TabZIP204.2	TRIAE_CS42_7AS_TGACv1_571414_AA1847500.2	343	37007.31	8.84	-0.401
TabZIP205	TRIAE_CS42_7BL_TGACv1_576759_AA1854020.1	167	18051.01	7.91	-0.662
TabZIP206	TRIAE_CS42_7BL_TGACv1_577418_AA1875080.1	247	26684.76	7.74	-0.641
TabZIP207	TRIAE_CS42_7BL_TGACv1_577539_AA1878150.1	255	27439.69	5.59	-0.515
TabZIP208	TRIAE_CS42_7BL_TGACv1_577569_AA1878740.1	165	17974.32	9.46	-0.565
TabZIP209.1	TRIAE_CS42_7BL_TGACv1_579567_AA1908510.1	302	32698.59	4.7	-0.497
TabZIP209.2	TRIAE_CS42_7BL_TGACv1_579567_AA1908510.2	293	31742.57	4.77	-0.501
TabZIP210	TRIAE_CS42_7BL_TGACv1_580489_AA1914260.1	247	26252.58	10.77	-0.353
TabZIP211	TRIAE_CS42_7BS_TGACv1_591894_AA1924840.1	340	36778.07	8.84	-0.423
TabZIP212	TRIAE_CS42_7BS_TGACv1_592661_AA1942670.1	468	50374.76	6.88	-0.843
TabZIP213	TRIAE_CS42_7BS_TGACv1_592867_AA1945500.1	370	39705.71	6.43	-0.614
TabZIP214	TRIAE_CS42_7BS_TGACv1_593422_AA1951360.1	223	24067.61	7.9	-0.735
TabZIP215	TRIAE_CS42_7BS_TGACv1_593476_AA1951870.1	184	20798.25	5.97	-0.751
TabZIP216	TRIAE_CS42_7DL_TGACv1_603253_AA1979190.1	207	22510.52	9.97	-1.272
TabZIP217.1	TRIAE_CS42_7DL_TGACv1_603981_AA1991930.1	349	38098.65	9.59	-0.773
TabZIP217.2	TRIAE_CS42_7DL_TGACv1_603981_AA1991930.2	348	37970.52	9.59	-0.765
TabZIP218	TRIAE_CS42_7DL_TGACv1_604895_AA2002830.1	250	27128.39	5.69	-0.525
TabZIP219.1	TRIAE_CS42_7DL_TGACv1_606407_AA2009990.1	302	32728.7	4.65	-0.472
TabZIP219.2	TRIAE_CS42_7DL_TGACv1_606407_AA2009990.2	278	30356.3	4.78	-0.427

TabZIP220.1	TRIAE_CS42_7DS_TGACv1_621925_AA2029490.1	418	45075.92	6.6	-0.251
TabZIP220.2	TRIAE_CS42_7DS_TGACv1_621925_AA2029490.3	448	48437.67	7.36	-0.301
TabZIP221	TRIAE_CS42_7DS_TGACv1_622069_AA2032220.1	221	23820.33	7.83	-0.715
TabZIP222	TRIAE_CS42_7DS_TGACv1_623432_AA2053120.1	446	50256.21	6.08	-0.7
TabZIP223	TRIAE_CS42_7DS_TGACv1_624207_AA2059750.1	473	50965.37	6.62	-0.871
TabZIP224	TRIAE_CS42_7DS_TGACv1_625219_AA2064590.1	368	39581.57	6.81	-0.654
TabZIP225	TRIAE_CS42_7DS_TGACv1_625303_AA2064840.1	345	37304.66	8.84	-0.408
TabZIP226.1	TRIAE_CS42_U_TGACv1_640702_AA2069780.1	365	39070.28	7.77	-0.516
TabZIP226.2	TRIAE_CS42_U_TGACv1_640702_AA2069780.2	366	39198.41	7.77	-0.524
TabZIP226.3	TRIAE_CS42_U_TGACv1_640702_AA2069780.3	368	39018.23	5.13	-0.287
TabZIP227	TRIAE_CS42_U_TGACv1_640756_AA2072630.1	384	40921.05	6.52	-0.935
TabZIP228.1	TRIAE_CS42_U_TGACv1_641199_AA2087990.1	324	34967.18	6.84	-0.503
TabZIP228.2	TRIAE_CS42_U_TGACv1_641199_AA2087990.2	457	49905.01	6.74	-0.492
TabZIP228.3	TRIAE_CS42_U_TGACv1_641199_AA2087990.3	458	50061.2	6.96	-0.501
TabZIP229.1	TRIAE_CS42_U_TGACv1_641253_AA2089870.1	496	53310.67	8.64	-0.161
TabZIP229.2	TRIAE_CS42_U_TGACv1_641253_AA2089870.2	418	45102.97	6.6	-0.234
TabZIP229.3	TRIAE_CS42_U_TGACv1_641253_AA2089870.3	448	48464.72	7.36	-0.285
TabZIP230	TRIAE_CS42_U_TGACv1_641258_AA2089960.1	253	27366.62	5.69	-0.509
TabZIP231.1	TRIAE_CS42_U_TGACv1_641308_AA2091490.2	483	52049.17	9.22	-0.525
TabZIP231.2	TRIAE_CS42_U_TGACv1_641308_AA2091490.3	464	49919.88	9.18	-0.456
TabZIP232.1	TRIAE_CS42_U_TGACv1_641545_AA2097630.1	382	40391.52	9.29	-0.586
TabZIP232.2	TRIAE_CS42_U_TGACv1_641545_AA2097630.2	328	35371.26	9.52	-0.5
TabZIP233	TRIAE_CS42_U_TGACv1_642196_AA2113120.1	167	18154.09	6.53	-0.699
TabZIP234	TRIAE_CS42_U_TGACv1_642368_AA2116410.1	164	18035.44	9.42	-0.591
TabZIP235.1	TRIAE_CS42_U_TGACv1_643015_AA2126130.1	228	24030.02	6.02	-0.509
TabZIP235.2	TRIAE_CS42_U_TGACv1_643015_AA2126130.2	250	26779.04	8.01	-0.727
TabZIP236	TRIAE_CS42_U_TGACv1_643396_AA2131620.1	327	35401.03	6.63	-0.639
TabZIP237.1	TRIAE_CS42_U_TGACv1_645006_AA2142650.1	321	33993.22	5.69	-1.029
TabZIP237.2	TRIAE_CS42_U_TGACv1_645006_AA2142650.2	345	36401.85	5.96	-0.992
TabZIP237.3	TRIAE_CS42_U_TGACv1_645006_AA2142650.4	317	33322.25	5.81	-1.032
TabZIP238.1	TRIAE_CS42_U_TGACv1_645734_AA2145290.1	413	45059.97	6.84	-0.259
TabZIP238.2	TRIAE_CS42_U_TGACv1_645734_AA2145290.2	443	48312.61	7.86	-0.306

Supplementary Table S3: Detail of the conserved motifs identified in wheat bZIP (TabZIP) proteins predicted by MEME database in each phylogenetic group.

Phylogenetic group	Motif no.	Motif length	Blast E value	Multiple consensus sequences	Number of TabZIP proteins containing motif	Name of TabZIP proteins containing motifs
	1	23	4.9e-4822	[KR]RQ[RK]R[ML][ALI][SQK]NRE[SA]A[RA][RK]SR[LE]RK[QK]AY	83	TabZIP3,8.1,8.2,12,19.1,19.2,19.3,25,26,27,28,33,37,40,41,44,46,47,48.1,48.2,49,53,57,62,63,64,66,67,71,72,73,74,75,78,84.1,84.2,86,87,90.1,90.2,103,105,110,115,118,128,129.1,129.2,129.3,132.1,132.2,132.3,134,143,149,159.1,159.2,165,170.1,170.2,175.1,175.2,175.3,183.1,183.2,190.1,190.2,190.3,197,204.1,204.2,208,211,225,226.1,226.2,226.3,231.1,231.2,234,235.1,235.2,236
**A**	7	21	3.2e-1810	ELER[EKQ]VSXLRAENXXLKX[RQE]LX	83	TabZIP3,8.1,8.2,12,19.1,19.2,19.3,25,26,27,28,33,37,40,41,44,46,47,48.1,48.2,49,53,57,62,63,64,66,67,71,72,73,74,75,78,84.1,84.2,86,87,90.1,90.2,103,105,110,115,118,128,129.1,129.2,129.3,132.1,132.2,132.3,134,143,149,159.1,159.2,165,170.1,170.2,175.1,175.2,175.3,183.1,183.2,190.1,190.2,190.3,197,204.1,204.2,208,211,225,226.1,226.2,226.3,231.1,231.2,234,235.1,235.2,236
	8	21	2.1e-841	GX[PT][LF][GS]SM[NT][MLV][DE]E[FL][LW]RNIWX[AV]EE	62	TabZIP3,8.1,8.2,12,19.1,19.2,19.3,33,44,53,57,62,63,64,66,67,71,72,73,74,75,78,84.1,84.2,86,87,90.1,90.2,103,110,118,129.1,129.2,129.3,132.1,132.2,132.3,143,159.1,159.2,170.1,170.2,175.1,175.2,175.3,183.1,183.2,190.1,190.2,190.3,204.1,204.2,211,225,226.1,226.2,226.3,231.1,231.2,235.1,235.2,236
	1	23	4.9e-4822	[KR]RQ[RK]R[ML][ALI][SQK]NRE[SA]A[RA][RK]SR[LE]RK[QK]AY	14	TabZIP4,14,21,32,42.1,42.2,42.3,52,198.1,198.2,209.1,209.2,219.1,219.2
**B**	7	21	3.2e-1810	ELER[EKQ]VSXLRAENXXLKX[RQE]LX	14	TabZIP4,14,21,32,42.1,42.2,42.3,52,198.1,198.2,209.1,209.2,219.1,219.2
	8	21	2.1e-841	GX[PT][LF][GS]SM[NT][MLV][DE]E[FL][LW]RNIWX[AV]EE	6	TabZIP32,198.1,209.1,209.2,219.1,219.2
	1	23	4.9e-4822	[KR]RQ[RK]R[ML][ALI][SQK]NRE[SA]A[RA][RK]SR[LE]RK[QK]AY	48	TabZIP1,11,18,125,135,140,141,151,155.1,155.2,155.3,158,168,172.1,172.2,172.3,172.4,176,179,181,182,187,188.1,188.2,188.3,188.4,188.5,188.6,191,193.1,193.2,193.3,194.1,194.2,194.3,196,200,202,205,206,210,214,215,217.1,217.2,221,222,233
**C**	7	21	3.2e-1810	ELER[EKQ]VSXLRAENXXLKX[RQE]LX	46	TabZIP11,18,125,135,140,141,151,155.1,155.2,155.3,158,168,172.1,172.2,172.3,172.4,176,179,181,182,187,188.1,188.2,188.3,188.4,188.5,188.6,191,193.1,193.2,193.3,194.1,194.2,196,200,202,205,206,210,214,215,217.1,217.2,221,222,233
	8	21	2.1e-841	GX[PT][LF][GS]SM[NT][MLV][DE]E[FL][LW]RNIWX[AV]EE	2	TabZIP155.1,172.4

D

1	23	4.9e-4822	[KR]RQ[RK]R[ML][ALI][SQK]NRE[SA]A[RA][RK]SR[LE]RK[QK]AY	83	TabZIP6.1,6.2,6.3,9,13.1,13.2,13.3,13.4,13.5,17.1,17.2,17.3,20.1,20.2,23,30.1,30.2,38.1,38.2,38.3,38.4,50.1,50.2,50.3,59.1,59.2,59.3,59.4,59.5,59.6,60,68.1,68.2,77.1,77.2,77.3,77.4,77.5,77.6,77.7,77.8,81,82,88,91.1,91.2,91.3,91.4,91.5,95.1,95.2,96.1,96.2,96.3,106.1,106.2,117.1,117.2,117.3,120.1,120.2,120.3,123,126,142.1,142.2,152,164,167.1,167.2,177,184.1,184.2,220.1,220.2,228.1,228.2,228.3,229.1,229.2,229.3,238.1,238.2
2	35	3.4e-2316	DVFH[LV][LM][ST]G[MA]WA[TS]PAER[CF]F[LF]W[LM]GGFRPSE[LV]LK[IVL]L[IA][GP]	80	TabZIP6.1,6.2,6.3,9,13.1,13.2,13.3,13.4,13.5,17.1,17.2,17.3,20.1,20.2,23,30.1,30.2,38.1,38.2,38.3,38.4,50.1,50.2,50.3,59.1,59.2,59.3,59.4,59.5,59.6,60,68.1,68.2,77.1,77.2,77.3,77.4,77.5,77.6,77.7,77.8,81,82,88,91.1,91.2,91.3,91.4,91.5,95.1,95.2,106.1,106.2,117.1,117.2,117.3,120.1,120.2,120.3,123,126,142.1,142.2,152,164,167.1,167.2,177,184.1,184.2,220.1,220.2,228.1,228.2,228.3,229.1,229.2,229.3,238.1,238.2
3	50	4.5e-2816	F[YVL][RQ]QADNLR[QL]QTL[HQ]QM[RH]RILTTRQAAR[CA][FL][LV][SVA][IL][GS][DE]Y[FY][RS]RLRALSSLW[AL][AS]RP	72	TabZIP6.1,6.2,6.3,9,13.1,13.2,13.3,13.4,13.5,17.1,17.2,17.3,20.1,20.2,23,30.1,30.2,38.1,38.2,38.3,50.1,50.2,50.3,59.1,59.2,59.3,59.4,59.5,60,68.1,77.2,77.3,77.4,77.5,77.6,77.7,77.8,81,82,88,91.2,91.3,91.4,91.5,95.1,95.2,106.2,117.1,117.2,117.3,120.1,120.2,120.3,123,126,142.1,142.2,152,164,167.1,167.2,177,184.1,184.2,220.1,220.2,228.2,228.3,229.1,229.2,229.3,238.1,238.2
4	41	2.4e-2025	[MA]FD[MV]EYARW[LV][DE][ED][DH][NG][KR][RH][MIL][AN]ELR[GA][AG][LV][QN]AH[LA][AG]DS[DEN]L[GR]AIV[ED]EC[ML]	82	TabZIP6.1,6.2,6.3,9,13.1,13.2,13.3,13.4,13.5,17.1,17.2,17.3,20.1,20.2,23,30.1,30.2,38.1,38.2,38.3,38.4,50.1,50.2,50.3,59.1,59.2,59.3,59.4,59.5,59.6,60,68.1,68.2,77.1,77.2,77.3,77.4,77.5,77.6,77.7,77.8,81,82,88,91.1,91.2,91.3,91.4,91.5,95.1,95.2,96.1,96.2,106.1,106.2,117.1,117.2,117.3,120.1,120.2,120.3,123,126,142.1,142.2,152,164,167.1,167.2,177,184.1,184.2,220.1,220.2,228.1,228.2,228.3,229.1,229.2,229.3,238.1,238.2
5	50	2.1e-2223	QL[ED]PLTEQQ[LM][MV]GI[CY][NG]LQ[QH]SS[EQ]QAE[ED]AL[SA]QG[LM][QE][QA]L[HQ]QSL[AS][DE]T[VL]A[AS]GTL[NA][DS]G	76	TabZIP6.1,6.2,6.3,9,13.1,13.2,13.3,13.4,13.5,17.1,17.2,17.3,20.1,20.2,23,30.1,30.2,38.1,38.2,38.3,50.1,50.2,50.3,59.1,59.2,59.3,59.4,59.5,59.6,60,68.1,77.1,77.2,77.3,77.4,77.5,77.6,77.7,77.8,81,82,88,91.1,91.2,91.3,91.4,91.5,95.2,106.1,106.2,117.1,117.2,117.3,120.1,120.2,120.3,123,126,142.1,142.2,152,164,167.1,167.2,177,184.1,184.2,220.1,220.2,228.2,228.3,229.1,229.2,229.3,238.1,238.2
6	41	1.1e-1901	[VI]Q[QN]LE[TS]SR[IVL][RK]L[QA]Q[LMI]EQELQRAR[QS]QGI[FL][LI][GS]G[GS]G[AD][GQ]GD[MSL]S[SP][GA]A	82	TabZIP6.1,6.2,6.3,9,13.1,13.2,13.3,13.4,13.5,17.1,17.2,17.3,20.1,20.2,23,30.1,30.2,38.1,38.2,38.3,38.4,50.1,50.2,50.3,59.1,59.2,59.3,59.4,59.5,59.6,60,68.1,68.2,77.1,77.2,77.3,77.4,77.5,77.6,77.7,77.8,81,82,88,91.1,91.2,91.3,91.4,91.5,95.1,95.2,96.1,96.2,106.1,106.2,117.1,117.2,117.3,120.1,120.2,120.3,123,126,142.1,142.2,152,164,167.1,167.2,177,184.1,184.2,220.1,220.2,228.1,228.2,228.3,229.1,229.2,229.3,238.1,238.2
9	21	4.6e-734	[NPD][VG][AP]NY[MT][GA][QI]MA[IL]A[LM][GE]KL[AG][STN]LE[SN]	63	TabZIP6.1,6.2,6.3,9,13.1,13.2,13.3,13.4,13.5,17.1,17.2,17.3,20.1,20.2,23,59.1,59.2,59.3,59.4,59.5,59.6,60,68.1,77.1,77.2,77.3,77.4,77.5,77.6,77.7,77.8,82,88,91.1,91.2,91.3,91.4,91.5,95.1,95.2,106.1,106.2,117.1,117.2,117.3,120.1,120.2,120.3,123,152,164,177,184.1,184.2,220.1,220.2,228.2,228.3,229.1,229.2,229.3,238.1,238.2
10	23	2.1e-606	[AQ][GE][RQ]P[PQ]TL[EN]IFPSWPM[PH]HPQQ[LP]H[SP]	43	TabZIP6.1,6.2,6.3,13.1,13.3,13.4,13.5,20.1,59.1,59.3,59.4,59.5,59.6,77.1,77.2,77.3,77.4,77.5,77.6,77.7,77.8,91.1,91.2,91.3,91.4,91.5,96.1,96.2,96.3,120.1,120.2,120.3,126,142.1,142.2,167.1,167.2,177,184.1,184.2,228.1,228.2,228.3

**E**	1	23	4.9e-4822	[KR]RQ[RK]R[ML][ALI][SQK]NRE[SA]A[RA][RK]SR[LE]RK[QK]AY	14	TabZIP35,45,55,61,70,83,92,93.1,93.2,124,145.1,145.2,145.3,161
	7	21	3.2e-1810	ELER[EKQ]VSXLRAENXXLKX[RQE]LX	13	TabZIP35,45,55,61,70,83,92,93.1,93.2,124,145.1,145.2,161
**F**	1	23	4.9e-4822	[KR]RQ[RK]R[ML][ALI][SQK]NRE[SA]A[RA][RK]SR[LE]RK[QK]AY	14	TabZIP5,65,79.1,79.2,89,139.1,139.2,154,173.1,173.2,199,207,218,230
	7	21	3.2e-1810	ELER[EKQ]VSXLRAENXXLKX[RQE]LX	14	TabZIP5,65,79.1,79.2,89,139.1,139.2,154,173.1,173.2,199,207,218,230
	8	21	2.1e-841	GX[PT][LF][GS]SM[NT][MLV][DE]E[FL][LW]RNIWX[AV]EE	2	TabZIP139.1,139.2
**G**	1	23	4.9e-4822	[KR]RQ[RK]R[ML][ALI][SQK]NRE[SA]A[RA][RK]SR[LE]RK[QK]AY	32	TabZIP7.2,16.1,16.2,31.1,31.2,31.4,54.2,54.3,54.4,54.5,54.6,101.1,101.2,108,114,136.1,136.2,162,171.1,171.2,178.1,178.2,178.3,189.1,189.2,189.3,227,232.1,232.2,237.1,237.2,237.3
	7	21	3.2e-1810	ELER[EKQ]VSXLRAENXXLKX[RQE]LX	29	TabZIP7.2,16.1,16.2,31.1,31.2,54.2,54.3,54.4,54.5,101.1,101.2,108,114,136.1,136.2,162,171.1,171.2,178.1,178.2,178.3,189.1,189.2,189.3,227,232.1,237.1,237.2,237.3
	8	21	2.1e-841	GX[PT][LF][GS]SM[NT][MLV][DE]E[FL][LW]RNIWX[AV]EE	6	TabZIP7.2,16.1,16.2,162,227,232.1
**H**	1	23	4.9e-4822	[KR]RQ[RK]R[ML][ALI][SQK]NRE[SA]A[RA][RK]SR[LE]RK[QK]AY	9	TabZIP69.1,69.2,80.1,80.2,80.3,94,186,195,216
	7	21	3.2e-1810	ELER[EKQ]VSXLRAENXXLKX[RQE]LX	8	TabZIP69.1,69.2,80.2,80.3,94,186,195,216
**I**	1	23	4.9e-4822	[KR]RQ[RK]R[ML][ALI][SQK]NRE[SA]A[RA][RK]SR[LE]RK[QK]AY	44	TabZIP2,15,22,29.1,29.2,34.1,34.2,34.3,36,43.1,43.2,51,56.1,56.2,56.3,97,100,104,109,111,113,116,119,122,130.1,130.2,131,137,146,147.1,147.2,150,157.1,157.2,157.3,157.4,163,169,201,203,212,213,223,224
	7	21	3.2e-1810	ELER[EKQ]VSXLRAENXXLKX[RQE]LX	44	TabZIP2,15,22,29.1,29.2,34.1,34.2,34.3,36,43.1,43.2,51,56.1,56.2,56.3,97,100,104,109,111,113,116,119,122,130.1,130.2,131,137,146,147.1,147.2,150,157.1,157.2,157.3,157.4,163,169,201,203,212,213,223,224
	8	21	2.1e-841	GX[PT][LF][GS]SM[NT][MLV][DE]E[FL][LW]RNIWX[AV]EE	15	TabZIP122,137,146,147.1,147.2,157.1,157.2,157.3,157.4,201,203,212,213,223,224
**S**	1	23	4.9e-4822	[KR]RQ[RK]R[ML][ALI][SQK]NRE[SA]A[RA][RK]SR[LE]RK[QK]AY	19	TabZIP10,24,58,76,85,99,102,107,112,133,138,144,153,160,166,174,180,185,192
	7	21	3.2e-1810	ELER[EKQ]VSXLRAENXXLKX[RQE]LX	12	TabZIP10,24,133,138,144,153,160,166,174,180,185,192
**U**	1	23	4.9e-4822	[KR]RQ[RK]R[ML][ALI][SQK]NRE[SA]A[RA][RK]SR[LE]RK[QK]AY	5	TabZIP98,121,127,148,156
	7	21	3.2e-1810	ELER[EKQ]VSXLRAENXXLKX[RQE]LX	5	TabZIP98,121,127,148,156

Supplementary Table S5: 89 TabZIPs (18+9+39+23) refer Figure 4B, differential ratio of log2 fold. Comparative differential log2 fold expression >2 fold (‘TAC 75’ vs ‘TAC 6’) were calculated using FPKM mean data values of biological replicates R1 and R2 in 'TAC 75' vs 'TAC 6', 'TAC 75' vs 'C 306' and 'TAC 6' vs 'C 306' .

TabZIPs	'TAC 75' vs 'TAC 6'	'TAC 75' vs 'C 306'	'TAC 6' vs 'C 306'
**TabZIP96.1**	**201.2**	0.7	-200.4
**TabZIP13.4**	**106.5**	-0.4	-106.9
**TabZIP184.2**	**87.4**	29.7	-57.8
**TabZIP117.2**	**78.6**	13.6	-64.9
**TabZIP34.3**	**63.7**	0.0	-63.7
**TabZIP120.2**	**51.7**	49.5	-2.1
**TabZIP167.2**	**45.6**	11.3	-34.4
**TabZIP54.4**	**37.0**	-0.5	-37.5
**TabZIP101.1**	**20.3**	-	-
**TabZIP173.2**	**14.7**	10.3	-4.4
**TabZIP235.1**	**14.3**	-1.5	-15.9
**TabZIP238.2**	**14.0**	-	-
**TabZIP189.3**	**13.9**	2.0	-11.9
**TabZIP95.1**	**13.7**	-0.2	-14.0
**TabZIP183.1**	**12.5**	0.0	-12.6
**TabZIP155.2**	**12.4**	1.0	-11.4
**TabZIP54.5**	**12.4**	14.8	2.4
**TabZIP77.6**	**11.7**	0.4	-11.2
**TabZIP188.1**	**10.7**	0.3	-10.4
**TabZIP54.3**	**9.6**	-1.8	-11.4
**TabZIP77.3**	**8.0**	1.0	-7.0
**TabZIP167.1**	**7.9**	1.0	-6.9
**TabZIP50.2**	**7.4**	-3.4	-10.9
**TabZIP56.3**	**7.1**	15.7	8.6
**TabZIP77.2**	**5.2**	45.0	39.9
**TabZIP77.4**	**4.0**	-1.7	-5.7
**TabZIP91.2**	**3.2**	2.2	-1.0
**TabZIP34.2**	**3.0**	8.1	5.1
**TabZIP77.5**	**2.6**	1.6	-1.0
**TabZIP59.4**	**2.3**	0.6	-1.7
**TabZIP31.4**	**2.2**	0.4	-1.9
**TabZIP227**	**2.2**	0.6	-1.7
**TabZIP156**	**2.2**	-0.2	-2.3
**TabZIP129.2**	**2.1**	-0.1	-2.3
**TabZIP136.1**	**2.1**	0.2	-2.0
**TabZIP175.1**	**-2.0**	0.0	2.0
**TabZIP147.2**	**-2.1**	-4.1	-2.1
**TabZIP143**	**-2.1**	-1.2	0.9
**TabZIP178.3**	**-2.2**	-1.9	0.3
**TabZIP179**	**-2.2**	-0.9	1.3
**TabZIP233**	**-2.3**	-1.2	1.1
**TabZIP13.2**	**-2.4**	5.0	7.4
**TabZIP120.3**	**-2.5**	-1.8	0.7
**TabZIP43.2**	**-2.5**	5.7	8.2
**TabZIP13.3**	**-2.5**	5.6	8.1
**TabZIP226.2**	**-2.7**	27.0	29.7
**TabZIP59.1**	**-2.8**	-3.2	-0.5
**TabZIP8.2**	**-2.9**	-1.1	1.8
**TabZIP149**	**-3.0**	-1.9	1.1
**TabZIP96.2**	**-3.5**	-1.7	1.8
**TabZIP132.2**	**-4.6**	-12.0	-7.4
**TabZIP13.1**	**-5.0**	9.0	14.0
**TabZIP188.2**	**-5.6**	0.8	6.3
**TabZIP129.3**	**-7.1**	-10.0	-2.9

**TabZIP188.5**	**-7.4**	-5.7	1.7
**TabZIP110**	**-7.6**	-7.3	0.3
**TabZIP189.1**	**-7.9**	5.6	13.4
**TabZIP59.5**	**-8.5**	-15.9	-7.5
**TabZIP172.2**	**-9.0**	-3.4	5.7
**TabZIP91.4**	**-9.5**	13.7	23.2
**TabZIP91.5**	**-9.6**	10.3	19.9
**TabZIP139.1**	**-10.3**	-1.9	8.4
**TabZIP238.1**	**-10.3**	-	-
**TabZIP136.2**	**-11.1**	0.2	11.3
**TabZIP155.1**	**-11.4**	-3.6	7.8
**TabZIP229.3**	**-12.0**	-	-
**TabZIP137**	**-12.1**	-13.2	-1.1
**TabZIP194.3**	**-12.5**	-11.3	1.2
**TabZIP117.1**	**-13.2**	-14.9	-1.7
**TabZIP219.1**	**-14.4**	-0.1	14.3
**TabZIP69.1**	**-15.3**	-1.3	14.0
**TabZIP38.4**	**-16.9**	28.6	45.5
**TabZIP190.1**	**-17.4**	-3.1	14.3
**TabZIP96.3**	**-17.7**	-26.6	-8.9
**TabZIP175.2**	**-18.0**	-2.8	15.2
**TabZIP54.1**	**-20.0**	-8.5	11.5
**TabZIP157.1**	**-22.2**	-22.7	-0.6
**TabZIP229.1**	**-22.5**	-	-
**TabZIP38.2**	**-26.8**	-33.5	-6.6
**TabZIP228.3**	**-29.7**	-4.2	25.6
**TabZIP228.1**	**-30.4**	0.4	30.8
**TabZIP178.1**	**-34.8**	75.0	109.7
**TabZIP59.2**	**-46.9**	-27.6	19.3
**TabZIP189.2**	**-54.4**	-51.6	2.8
**TabZIP77.7**	**-97.3**	-126.3	-29.0
**TabZIP59.6**	**-109.1**	-288.1	-179.0
**TabZIP77.1**	**-111.7**	-93.8	17.9
**TabZIP237.1**	**-153.1**	-151.3	1.8
**TabZIP237.3**	**-167.8**	-183.6	-15.7

Supplementary Table S5: 91 TabZIPs (39+9+8+35) refer Figure 4B, differential ratio of log2 fold. Comparative differential log2 fold expression >2 fold (‘TAC 75’ vs ‘C 306’) were calculated using FPKM mean data values of biological replicates R1 and R2 in 'TAC 75' vs 'C 306', 'TAC 75' vs 'TAC 6'' and 'TAC 6' vs 'C 306.

TabZIP	'TAC 75' vs 'C 306'	'TAC 75' vs 'TAC 6'	'TAC 6' vs 'C 306'
**TabZIP30.2**	**147.9**	1.2	146.7
**TabZIP50.1**	**77.5**	0.6	76.9
**TabZIP50.3**	**76.7**	-1.4	78.1
**TabZIP178.1**	**75.0**	-34.8	109.7
**TabZIP77.8**	**50.6**	0.0	50.7
**TabZIP120.2**	**49.5**	51.7	-2.1
**TabZIP77.2**	**45.0**	5.2	39.9
**TabZIP155.3**	**43.1**	-1.1	44.2
**TabZIP80.3**	**37.6**	-0.3	37.9
**TabZIP157.2**	**36.5**	0.6	36.0
**TabZIP142.1**	**34.6**	0.6	34.0
**TabZIP157.3**	**30.1**	-2.0	32.1
**TabZIP184.2**	**29.7**	87.4	-57.8
**TabZIP38.4**	**28.6**	-16.9	45.5
**TabZIP226.2**	**27.0**	-2.7	29.7
**TabZIP159.2**	**26.7**	1.0	25.7

**TabZIP38.1**	**20.2**	0.9	19.3
**TabZIP138**	**19.8**	-0.4	20.2
**TabZIP132.3**	**17.9**	-0.7	18.6
**TabZIP56.3**	**15.7**	7.1	8.6
**TabZIP140**	**15.1**	1.1	14.0
**TabZIP95.2**	**15.0**	0.4	14.6
**TabZIP54.5**	**14.8**	12.4	2.4
**TabZIP29.1**	**14.5**	0.3	14.1
**TabZIP91.4**	**13.7**	-9.5	23.2
**TabZIP117.2**	**13.6**	78.6	-64.9
**TabZIP13.5**	**12.7**	0.4	12.3
**TabZIP193.1**	**11.7**	1.0	10.7
**TabZIP167.2**	**11.3**	45.6	-34.4
**TabZIP188.4**	**10.4**	0.0	10.4
**TabZIP91.5**	**10.3**	-9.6	19.9
**TabZIP173.2**	**10.3**	14.7	-4.4
**TabZIP13.1**	**9.0**	-5.0	14.0
**TabZIP34.2**	**8.1**	3.0	5.1
**TabZIP56.1**	**6.8**	-1.5	8.3
**TabZIP43.2**	**5.7**	-2.5	8.2
**TabZIP13.3**	**5.6**	-2.5	8.1
**TabZIP189.1**	**5.6**	-7.9	13.4
**TabZIP13.2**	**5.0**	-2.4	7.4
**TabZIP18**	**4.6**	-0.1	4.6
**TabZIP48.2**	**4.1**	-0.7	4.9
**TabZIP104**	**3.7**	0.6	3.1
**TabZIP172.4**	**3.1**	-0.5	3.6
**TabZIP59.3**	**3.0**	1.6	1.4
**TabZIP237.2**	**2.9**	1.9	1.1
**TabZIP204.2**	**2.6**	-0.2	2.8
**TabZIP126**	**2.5**	0.2	2.3
**TabZIP11**	**2.4**	-0.4	2.8
**TabZIP91.2**	**2.2**	3.2	-1.0
**TabZIP122**	**2.1**	1.8	0.3
**TabZIP189.3**	**2.0**	13.9	-11.9
**TabZIP184.1**	**-2.1**	-0.8	-1.3
**TabZIP159.1**	**-2.4**	-1.5	-0.9
**TabZIP20.2**	**-2.4**	-1.4	-1.0
**TabZIP91.3**	**-2.5**	-0.3	-2.1
**TabZIP80.1**	**-2.8**	-1.7	-1.1
**TabZIP175.2**	**-2.8**	-18.0	15.2
**TabZIP188.3**	**-2.8**	-0.8	-2.1
**TabZIP90.2**	**-2.9**	0.8	-3.7
**TabZIP190.1**	**-3.1**	-17.4	14.3
**TabZIP59.1**	**-3.2**	-2.8	-0.5
**TabZIP172.2**	**-3.4**	-9.0	5.7
**TabZIP50.2**	**-3.4**	7.4	-10.9
**TabZIP155.1**	**-3.6**	-11.4	7.8
**TabZIP147.2**	**-4.1**	-2.1	-2.1
**TabZIP228.3**	**-4.2**	-29.7	25.6
**TabZIP188.5**	**-5.7**	-7.4	1.7
**TabZIP110**	**-7.3**	-7.6	0.3
**TabZIP16.2**	**-7.6**	-0.9	-6.7
**TabZIP54.1**	**-8.5**	-20.0	11.5
**TabZIP129.3**	**-10.0**	-7.1	-2.9
**TabZIP209.2**	**-10.8**	-0.2	-10.6
**TabZIP145.3**	**-10.8**	-	-
**TabZIP194.3**	**-11.3**	-12.5	1.2
**TabZIP131**	**-11.6**	1.1	-12.7
**TabZIP132.2**	**-12.0**	-4.6	-7.4
**TabZIP137**	**-13.2**	-12.1	-1.1
**TabZIP117.1**	**-14.9**	-13.2	-1.7

**TabZIP84.1**	**-15.1**	-1.9	-13.2
**TabZIP106.2**	**-15.7**	0.6	-16.3
**TabZIP59.5**	**-15.9**	-8.5	-7.5
**TabZIP157.1**	**-22.7**	-22.2	-0.6
**TabZIP96.3**	**-26.6**	-17.7	-8.9
**TabZIP59.2**	**-27.6**	-46.9	19.3
**TabZIP38.2**	**-33.5**	-26.8	-6.6
**TabZIP189.2**	**-51.6**	-54.4	2.8
**TabZIP77.1**	**-93.8**	-111.7	17.9
**TabZIP77.7**	**-126.3**	-97.3	-29.0
**TabZIP237.1**	**-151.3**	-153.1	1.8
**TabZIP237.3**	**-183.6**	-167.8	-15.7
**TabZIP59.6**	**-288.1**	-109.1	-179.0

Supplementary Table S5: 112 TabZIPs (23+39+35+15) refer Figure 4B, differential ratio of log2 fold. Comparative differential log2 fold expression >2 fold (‘TAC 6’ vs ‘C 306’) were calculated using FPKM mean data values of biological replicates R1 and R2 in 'TAC 75' vs 'TAC 6', 'TAC 75' vs 'C 306’' and 'TAC 6' vs 'C 306.

TabZIPs	'TAC 6' vs 'C 306'	'TAC 75' vs' TAC 6'	'TAC 75' vs 'C 306'
**TabZIP30.2**	**146.7**	1.2	147.9
**TabZIP178.1**	**109.7**	-34.8	75.0
**TabZIP17.3**	**105.2**	-	-
**TabZIP50.3**	**78.1**	-1.4	76.7
**TabZIP50.1**	**76.9**	0.6	77.5
**TabZIP77.8**	**50.7**	0.0	50.6
**TabZIP38.4**	**45.5**	-16.9	28.6
**TabZIP155.3**	**44.2**	-1.1	43.1
**TabZIP77.2**	**39.9**	5.2	45.0
**TabZIP80.3**	**37.9**	-0.3	37.6
**TabZIP157.2**	**36.0**	0.6	36.5
**TabZIP142.1**	**34.0**	0.6	34.6
**TabZIP157.3**	**32.1**	-2.0	30.1
**TabZIP228.1**	**30.8**	-30.4	0.4
**TabZIP226.2**	**29.7**	-2.7	27.0
**TabZIP159.2**	**25.7**	1.0	26.7
**TabZIP228.3**	**25.6**	-29.7	-4.2
**TabZIP91.4**	**23.2**	-9.5	13.7
**TabZIP138**	**20.2**	-0.4	19.8
**TabZIP91.5**	**19.9**	-9.6	10.3
**TabZIP59.2**	**19.3**	-46.9	-27.6
**TabZIP38.1**	**19.3**	0.9	20.2
**TabZIP132.3**	**18.6**	-0.7	17.9
**TabZIP77.1**	**17.9**	-111.7	-93.8
**TabZIP175.2**	**15.2**	-18.0	-2.8
**TabZIP95.2**	**14.6**	0.4	15.0
**TabZIP190.1**	**14.3**	-17.4	-3.1
**TabZIP219.1**	**14.3**	-14.4	-0.1
**TabZIP29.1**	**14.1**	0.3	14.5
**TabZIP13.1**	**14.0**	-5.0	9.0
**TabZIP140**	**14.0**	1.1	15.1
**TabZIP69.1**	**14.0**	-15.3	-1.3
**TabZIP189.1**	**13.4**	-7.9	5.6
**TabZIP13.5**	**12.3**	0.4	12.7
**TabZIP54.1**	**11.5**	-20.0	-8.5
**TabZIP136.2**	**11.3**	-11.1	0.2

**TabZIP193.1**	**10.7**	1.0	11.7
**TabZIP188.4**	**10.4**	0.0	10.4
**TabZIP56.3**	**8.6**	7.1	15.7
**TabZIP139.1**	**8.4**	-10.3	-1.9
**TabZIP20.1**	**8.4**	-	-
**TabZIP56.1**	**8.3**	-1.5	6.8
**TabZIP43.2**	**8.2**	-2.5	5.7
**TabZIP13.3**	**8.1**	-2.5	5.6
**TabZIP155.1**	**7.8**	-11.4	-3.6
**TabZIP13.2**	**7.4**	-2.4	5.0
**TabZIP188.2**	**6.3**	-5.6	0.8
**TabZIP172.2**	**5.7**	-9.0	-3.4
**TabZIP34.2**	**5.1**	3.0	8.1
**TabZIP48.2**	**4.9**	-0.7	4.1
**TabZIP18**	**4.6**	-0.1	4.6
**TabZIP172.4**	**3.6**	-0.5	3.1
**TabZIP104**	**3.1**	0.6	3.7
**TabZIP38.3**	**2.9**	-1.2	1.7
**TabZIP204.2**	**2.8**	-0.2	2.6
**TabZIP189.2**	**2.8**	-54.4	-51.6
**TabZIP11**	**2.8**	-0.4	2.4
**TabZIP152**	**2.7**	-1.1	1.7
**TabZIP34.1**	**2.5**	-0.7	1.9
**TabZIP54.5**	**2.4**	12.4	14.8
**TabZIP48.1**	**2.3**	-0.4	1.9
**TabZIP126**	**2.3**	0.2	2.5
**TabZIP231.2**	**2.2**	-1.5	0.7
**TabZIP228.2**	**2.2**	-0.9	1.2
**TabZIP226.1**	**2.1**	-2.0	0.2
**TabZIP175.1**	**2.0**	-2.0	0.0
**TabZIP19.1**	**2.0**	-0.2	1.8
**TabZIP188.3**	**-2.1**	-0.8	-2.8
**TabZIP147.2**	**-2.1**	-2.1	-4.1
**TabZIP91.3**	**-2.1**	-0.3	-2.5
**TabZIP127**	**-2.1**	1.7	-0.5
**TabZIP120.2**	**-2.1**	51.7	49.5
**TabZIP172.1**	**-2.3**	1.3	-1.0
**TabZIP129.2**	**-2.3**	2.1	-0.1
**TabZIP156**	**-2.3**	2.2	-0.2
**TabZIP148**	**-2.5**	1.9	-0.6
**TabZIP129.3**	**-2.9**	-7.1	-10.0
**TabZIP90.2**	**-3.7**	0.8	-2.9
**TabZIP173.2**	**-4.4**	14.7	10.3
**TabZIP77.4**	**-5.7**	4.0	-1.7
**TabZIP38.2**	**-6.6**	-26.8	-33.5
**TabZIP16.2**	**-6.7**	-0.9	-7.6
**TabZIP167.1**	**-6.9**	7.9	1.0
**TabZIP77.3**	**-7.0**	8.0	1.0
**TabZIP132.2**	**-7.4**	-4.6	-12.0
**TabZIP59.5**	**-7.5**	-8.5	-15.9
**TabZIP96.3**	**-8.9**	-17.7	-26.6
**TabZIP188.1**	**-10.4**	10.7	0.3
**TabZIP209.2**	**-10.6**	-0.2	-10.8
**TabZIP50.2**	**-10.9**	7.4	-3.4
**TabZIP77.6**	**-11.2**	11.7	0.4
**TabZIP54.3**	**-11.4**	9.6	-1.8
**TabZIP155.2**	**-11.4**	12.4	1.0
**TabZIP189.3**	**-11.9**	13.9	2.0
**TabZIP183.1**	**-12.6**	12.5	0.0
**TabZIP131**	**-12.7**	1.1	-11.6
**TabZIP84.1**	**-13.2**	-1.9	-15.1
**TabZIP95.1**	**-14.0**	13.7	-0.2

**TabZIP17.1**	**-15.2**	-	-
**TabZIP237.3**	**-15.7**	-167.8	-183.6
**TabZIP235.1**	**-15.9**	14.3	-1.5
**TabZIP106.2**	**-16.3**	0.6	-15.7
**TabZIP17.2**	**-19.1**	-	-
**TabZIP77.7**	**-29.0**	-97.3	-126.3
**TabZIP167.2**	**-34.4**	45.6	11.3
**TabZIP54.4**	**-37.5**	37.0	-0.5
**TabZIP184.2**	**-57.8**	87.4	29.7
**TabZIP34.3**	**-63.7**	63.7	0.0
**TabZIP117.2**	**-64.9**	78.6	13.6
**TabZIP13.4**	**-106.9**	106.5	-0.4
**TabZIP59.6**	**-179.0**	-109.1	-288.1
**TabZIP96.1**	**-200.4**	201.2	0.7

Supplementary Table S6: Nucleotide sequences of primer pairs designed for the selected fifty-two wheat bZIPs (TabZIPs) for real-time quantitative gene expression analysis (qRT-PCR).

TabZIP	Forward primer (F) (5'-3')	Reverse primer (R) (5'-3')	Amplicon size (bp)	Tm (°C) of F, R
TabZIP59.4	GGAGGAGGAGACCATGACAAGAGGA	TCCGAGAAATATCCCCTGTG	222	53.8, 55.4
TabZIP53	GGAGATCGAGCATCTCAAGG	AGAAGGAGGCCGAGTTGAC	211	53.8, 55.9
TabZIP122	TCATGGACAGTGAGCACCTCT	AAGCTTGCAGCCTGATTCTC	159	50.0, 49.7
TabZIP111	TCAAATACACAGGCGAGCTG	GTGCAGCGGCTTAAGATAGC	217	49.7, 50.5
TabZIP113	GACAGAACTTGAGCGGAAGG	GGCAATTCCCTTCATCTTGA	170	53.8, 49.7
TabZIP236	CAAGATTCGCCGAAGAGAAG	CCATGTTAGCTGGCAAGTCA	210	51.8, 49.1
TabZIP121	GCTGCGTGGAAGGAATAGAG	GAGGATATTTAAAGCGGGGG	159	53.8, 55.0
TabZIP91.2	GGAGGAGGAGACCATGACAA	CACAGGGGATATTTCTCGGA	222	55.1, 49.8
TabZIP69.1	GAGAGCGACGAGGAGATACG	GGAAGAAGGCCTACATGACG	231	54.9, 53.8
TabZIP13.3	CGACAACCTGAGACAGCAAA	AGAATTTCATGATGCCGGAG	160	53.8, 51.8
TabZIP38.2	GCTAGAGTCGAGCCGGATAA	TGTTCTGCGCAAGTCTTCTC	221	51.8, 53.8
TabZIP175.1	CCTCAACTTGTCCTCGCTGT	AAACAGGCGGAGATGCTAGAG	247	51.8, 53.8
TabZIP136.1	TACGCAAACAGCAAGAATGC	CGTTGTGACTACCCTGAGCA	207	53.8, 51.8
TabZIP188.2	TCGCAGTCGTCAATCTATGC	GACACCATCCGTCTCATCCT	163	53.8, 51.8
TabZIP137	CAGCAAGATCGAAGGAAAGG	ATGACACCCTGAAAACGGAG	221	51.8, 51.1
TabZIP120.2	CTGCCACGGTGACTAATCCT	TTTTCTTGCAGCCTCTCGAT	200	55.9, 53.8
TabZIP143	GGGCCCAAATTCAGACACTA	ACTGTACCCCTGCCATTGAG	241	53.8, 51.8
TabZIP228.3	TTCTACCGCTGCTACCTCGT	TCAACATCTTCCCTTCTCGG	166	51.1, 49.7
TabZIP38.1	AGGCCTACATCCAGCAGCTA	AGTTCGAGATCGAGTACGGG	221	47.7, 53.8
TabZIP148	GTCGTCACAAAACGAGAGCA	GCTTCCTTTACGCAACAGCT	244	51.8, 53.8
TabZIP167.1	TAGAGAGGCAGCCAGGAAAA	TGGAAGATCAGCTTGCTGTG	216	53.8, 49.7
TabZIP147.1	CCTGCAGACAGAAGCAACAA	AATGACACCCTGAAAACGGAG	160	51.8, 51.8
TabZIP29.1	AAGAGGATCATGGCAAATCG	TGGTGATTTGAAGCTGCAAG	187	53.8, 53.8
TabZIP152.2	AATACAGACCCTGCCAGTGC	ATCTTTGCTAAAGCGCCTGA	157	53.8, 51.8
TabZIP151	AATCCCATAGGAGGCAAGGT	GGAATGGGGTCCCTACTGAT	248	51.8, 51.8
TabZIP50.2	GACGAGGATCCACAGATGGA	AAAAAGGCCTACATCCAGCA	149	51.1, 53.3
TabZIP36	GCAGCTCCAAATCCACTCTC	CATGGCAATTCCCTTCATCT	160	51.1, 51.8
TabZIP51	TCGTACGGAGTCAATCCACA	TCAAGATGAAGGGAATTGCC	246	51.8, 51.8
TabZIP54.1	TGTCTCCTGAGAAGGGCAGTT	TATGCTGCTGTCGAGTCACC	211	51.8, 51.8
TabZIP97	CTGGAGAGGAAGGTGCAGAC	AAATGCTCTGAATGATGCCC	169	53.8, 51.8
TabZIP98	AGGAAAGTCGCGGATTTAGC	GTCGTCTACACCAGCGATCA	157	53.8, 51.8
TabZIP123.1	GGGTCAGCAACAACAAGGAT	GCCTATATCCAGCAGCTCGA	163	51.8, 53.8
TabZIP157.1	GTCTGCAGAACATGGAGCAG	AGTCTCTCCTAGCGACGCAC	218	53.8, 55.9
TabZIP160	GCCAACAAATGAACATGGTG	AGCAGTGCCTACGACGTTTT	208	49.7, 51.8
TabZIP158	TTGCAGCTAGTCCGTCATTG	GGAATGGTCCCCCTACTGAT	248	51.8, 53.8
TabZIP219.1	TCTACCCAACCGAAGTCTGG	CAACGACCCCACTACTCCAT	209	53.8, 51.8
TabZIP167.2	GATTTCGATCAAACGGCAAT	CTCCAAAGAGGCCTGAACAG	216	47.7, 53.8
TabZIP156	CACGTGTCCGTGCTACCTAC	TGCTCCCATGTCTGATCGTA	185	53.8, 49.7
TabZIP159.1	GAATTCAGGCAGTCACAGCA	CAGATGTCGTTTGGGGAGAT	186	51.8, 51.8
TabZIP56.3	GCCTTTCCGCTGAAAATACA	TCAAGTGTTCATCAGCTGCC	247	49.7, 53.8
TabZIP52	CAAGAAGGTTGCGAGTGTCA	GATGGTGTCTCGGAAGGTGT	192	53.2, 55.9
TabZIP193.1	TCGCAGTCGTCATTCTATGC	GAACCCAACGGACGTTAAGA	166	53.8, 55.9
TabZIP120.3	CAGGCCAGTTCCATCAGATT	AATCGAGAGGCTGCAAGAAA	152	55.9, 53.8
TabZIP198.1	CATGAAGTCCAGGGAGAGGA	GCTTGATCCTCTCCCTTGTG	204	51.8-53.8
TabZIP203	GCATGCAGAAGCAACAACAT	TTCAGTGCGTCATTCAGAGC	197	49.7, 49.7
TabZIP209.1	AGGCCTATTGGTGCTCACAC	AAGGAGAGCCGAAAGAGGTC	160	53.8, 53.8
TabZIP208	CTGTCTCGTTTGGTGGGTTC	TGGATGACAACCTCAAGCTG	206	53.8, 50.3
TabZIP212	CAACTATGCCACAGGTGACG	GGTCAAGAGGATTTTGGCAA	230	51.8, 49.7
TabZIP216	CCAACAAAGGAACAGCCAAT	CAGCTCCAACTCCAAGGAAG	182	49.7, 51.8
TabZIP219.2	TTTGGCTAGTGAGCATCGTG	GCTAGATACTCCGCCATTGC	204	51.8, 53.8
TabZIP8.2	GGGGAGATGACACTTGAGGA	CAGGTCCTTTCATGGCAAGT	210	51.8, 51.8
TabZIP224	ATGCTCTGAATGACGCACTG	AGATGCAGCAGATTCACCCT	197	51.8, 51.8

Supplementary Table S7: **Differential gene expression of 52 wheat bZIPs (TabZIPs) in wheat mutant lines**. The real-time quantitative gene expression level was measured at three seed developmental stages (21, 28, and 35 days after anthesis, DAA) in the high amylose mutant line 'TAC 75' with ~ 65% amylose content, the low amylose mutant line 'TAC 6' with ~7 % amylose content, and parent variety 'C 306' with ~26% amylose content. The data were calculated as mean and SD of three technical replicates of log_2 of ratio of their ΔC_T values. ΔC_T was calculated following Schmittgen and Livak (2008). Wheat ADP ribosylation factor (ARF) was used as an internal control gene for normalization.

TabZIPs	'TAC 75' vs 'TAC 6'			'TAC 75' vs 'C 306'			'TAC 6' vs 'C 306'		
	21 DAA	28 DAA	35 DAA	21 DAA	28 DAA	35 DAA	21 DAA	28 DAA	35 DAA
TabZIP59.4	-07.8 ± 0.06	02.0 ± 0.06	-00.7 ± 0.11	-12.3 ± 0.03	-08.0 ± 0.02	07.6 ± 0.05	-7.15±0.04	-2.02±0.04	8.35±0.08
TabZIP53	-07.6 ± 0.04	06.8 ± 0.06	05.6 ± 0.19	-10.7 ± 0.00	04.5 ± 0.01	11.0 ± 0.00	-12.63±0.02	6.89±0.04	5.39±0.10
TabZIP122	-05.9 ± 0.06	-05.7 ± 0.10	10.9 ± 0.17	-05.8 ± 0.04	05.8 ± 0.02	11.6 ± 0.02	-12.35±0.03	5.70±0.06	0.65±0.10
TabZIP111	-06.8 ± 0.03	-04.3 ± 0.00	-07.2 ± 0.11	-17.7 ± 0.03	-09.6 ± 0.01	-06.7 ± 0.04	-6.20±0.02	4.38±0.01	0.56±0.08
TabZIP113	-06.0 ± 0.08	-03.6 ± 0.01	03.4 ± 0.15	-11.9 ± 0.01	-01.9 ± 0.01	04.1 ± 0.09	-11.16±0.04	3.70±0.01	0.71±0.11
TabZIP236	-05.7 ± 0.05	-04.0 ± 0.04	-03.5 ± 0.47	-13.8 ± 0.02	-07.3 ± 0.05	-02.8 ± 0.02	-10.98±0.02	4.04±0.01	0.70±0.23
TabZIP121	-06.1 ± 0.05	-03.5 ± 0.09	-34.0 ± 0.43	-15.2 ± 0.03	-30.1 ± 0.01	-25.0 ± 0.03	-7.26±0.03	3.57±0.04	9.02±0.20
TabZIP91.2	03.0 ± 0.35	10.7 ± 1.25	03.4 ± 0.34	00.7 ± 0.35	01.8 ± 0.46	0.06 ± 0.76	-8.74±0.08	4.55±0.02	9.00±0.05
TabZIP69.1	-04.4 ± 0.05	-05.8 ± 0.02	-15.7 ± 0.52	-08.9 ± 0.02	-10.8 ± 0.03	-07.3 ± 0.01	-0.23±0.03	5.80±0.03	8.37±0.27
TabZIP13.3	-05.6 ± 0.04	-04.7 ± 0.00	-05.2 ± 0.12	-12.7 ± 0.02	00.9 ± 0.03	06.3 ± 0.24	-11.94±0.02	4.78±0.02	11.59±0.16
TabZIP38.2	-15.5 ± 0.02	00.5 ± 0.06	04.9 ± 0.01	-05.0 ± 0.06	-25.0 ± 0.02	10.1 ± 0.00	-4.19±0.03	-0.51±0.03	5.20±0.01
TabZIP175.1	-08.7 ± 0.04	-04.1 ± 0.04	-02.5 ± 0.08	-06.8 ± 0.12	-01.9 ± 0.01	06.3 ± 0.02	-5.30±0.06	4.16±0.02	8.86±0.06
TabZIP136.1	-07.8 ± 0.06	07.0 ± 0.03	-09.4 ± 0.01	-13.8 ± 0.06	35.0 ± 0.02	-01.5 ± 0.02	-5.70±0.05	7.08±0.01	7.87±0.02
TabZIP188.2	-07.7 ±0.00	-06.0 ± 0.02	02.3 ± 0.12	-00.2 ± 0.02	03.7 ± 0.05	11.2 ± 0.03	-4.24±0.01	6.02±0.03	8.95±0.05
TabZIP137	-08.0 ± 0.05	-04.5 ± 0.02	-03.0 ± 0.04	-06.6 ± 0.03	-01.2 ± 0.16	07.7 ± 0.02	-5.43±0.04	4.53±0.08	10.84±0.03
TabZIP120.2	20.9 ± 1.00	19.3 ± 1.17	26.0 ± 1.30	12.3 ± 0.18	16.9 ± 8.85	21.2 ± 6.40	-0.37±0.08	5.08±0.03	8.78±0.06
TabZIP143	-04.9 ± 0.03	-07.0 ± 0.29	-03.6 ± 0.04	-05.4 ± 0.06	-01.4 ± 0.03	03.5 ± 0.06	-4.74±0.03	7.06±0.16	7.12±0.05
TabZIP228.3	-07.0 ± 0.08	-03.0 ± 0.79	-00.2 ± 0.21	-02.9 ± 0.01	-02.6 ± 0.12	10.7 ± 0.03	-5.74±0.04	3.01±0.45	11.05±0.10
TabZIP38.1	-06.4 ± 0.01	-04.6 ± 0.05	-04.1 ± 0.03	-06.4 ± 0.04	-02.1 ± 0.01	04.9 ± 0.06	-3.26±0.02	4.66±0.02	9.10±0.03
TabZIP148	-07.0 ± 0.12	-05.2 ± 0.45	-05.4 ± 0.09	-05.8 ± 0.04	-03.0 ± 0.01	3.2 ± 0.025	-4.86±0.09	5.25±0.24	8.76±0.06
TabZIP167.1	-02.6 ± 0.07	-04.6 ± 0.02	-08.1 ± 0.23	-04.6 ± 0.04	-04.6 ± 0.01	00.8 ± 0.10	-5.13±0.03	4.65±0.02	8.98±0.16
TabZIP147.1	-02.3 ± 0.01	-04.8 ± 0.11	-09.1 ± 0.20	-06.0 ± 0.00	-01.1 ± 0.0	-00.7 ± 0.02	-5.09±0.01	4.86±0.05	8.43±0.10
TabZIP29.1	-03.3 ± 0.05	-03.3 ± 0.83	-04.8 ± 0.11	-03.5 ± 0.07	-02.4 ± 0.02	02.8 ± 0.03	-3.68±0.05	3.38±0.42	7.68±0.06
TabZIP152.2	-07.0 ± 0.01	-03.6 ± 0.15	-07.0 ± 0.08	-09.9 ± 0.05	-04.0 ± 0.09	03.2 ± 0.03	-4.56±0.03	3.65±0.09	10.24±0.06
TabZIP151	-02.1 ± 0.03	-15.1 ± 0.65	-12.3 ± 0.05	-08.9 ± 0.02	-05.2 ± 0.04	-04.2 ± 0.05	-4.50±0.01	15.15±0.35	8.07±0.02
TabZIP50.2	-08.1 ± 0.04	-10.3 ± 0.09	19.8 ± 0.00	21.0 ± 0.01	27.8 ± 0.04	32.7 ± 0.02	-0.95±0.04	10.36±0.04	12.95±0.01
TabZIP36	-08.1 ± 0.02	-04.3 ± 0.12	-07.4 ± 0.04	-06.8 ± 0.03	-03.4 ± 1.04	04.6 ± 0.04	-2.34±0.00	4.38±0.07	12.05±0.02
TabZIP51	00.8 ± 0.02	-05.1 ± 0.02	-03.4 ± 0.02	08.0 ± 0.03	06.9 ± 0.00	06.6 ± 0.03	0.78±0.02	5.13±0.01	10.15±0.03

TabZIP54.1	-03.3 ± 0.03	-04.1 ± 0.16	-04.3 ± 0.06	-01.0 ± 0.06	-05.2 ± 0.02	02.4 ± 0.05	1.97±0.01	4.18±0.08	6.79±0.01
TabZIP97	-07.8 ± 0.08	-07.0 ± 0.18	02.2 ± 0.04	02.0 ± 0.01	04.4 ± 0.01	11.4 ± 0.03	-3.96±0.04	7.08±0.10	9.18±0.02
TabZIP98	-06.7 ± 0.24	-03.5 ± 0.04	-11.2 ± 0.07	-10.5 ± 0.15	-08.3 ± 0.03	-00.3 ± 0.03	-3.81±0.05	3.59±0.04	10.87±0.04
TabZIP123	-03.5 ± 0.04	-21.1 ± 0.01	04.6 ± 0.02	-03.7 ± 0.01	13.9 ± 0.02	05.1 ± 0.06	-4.91±0.04	21.10±0.01	0.50±0.03
TabZIP157.1	-17.8 ± 0.03	-20.1 ± 0.26	10.7 ± 0.14	-11.8 ± 0.02	19.9 ± 0.03	11.4 ± 0.01	-4.83±0.03	20.10±0.16	0.68±0.08
TabZIP160	-08.2 ± 0.04	-19.0 ± 0.09	07.6 ± 0.02	-03.5 ± 0.03	17.7 ± 0.02	09.5 ± 0.04	-1.11±0.01	19.07±0.06	1.83±0.03
TabZIP158	-10.7± 0.04	-20.4 ± 0.51	11.5 ± 0.04	-03.4 ± 0.01	21.0 ± 0.03	12.0 ± 0.03	-5.77±0.03	20.44±0.25	0.55±0.03
TabZIP219.1	-15.8 ± 0.09	-20.3 ± 0.18	06.3 ± 0.03	-12.1 ± 0.02	16.1 ± 0.07	09.2 ± 0.00	-4.11±0.06	20.32±0.09	2.89±0.02
TabZIP167.2	20.4 ± 0.09	13.8 ± 4.51	19.6 ± 0.58	04.0 ± 0.72	03.0 ± 1.92	02.1 ± 2.05	-5.53±0.03	21.61±0.08	4.59±0.02
TabZIP156	-08.4 ± 0.08	-18.2 ± 0.41	07.3 ± 0.04	-02.9 ± 0.02	16.5 ± 0.00	10.2 ± 0.01	-1.93±0.06	18.23±0.24	2.85±0.03
TabZIP159.1	-14.4 ± 0.10	-19.1 ± 0.06	09.2 ± 0.02	-09.2 ± 0.04	18.3 ± 0.04	10.1 ± 0.10	-5.21±0.03	19.10±0.09	0.86±0.07
TabZIP56.3	07.0 ± 0.55	-04.7 ± 0.26	04.8 ± 0.55	14.6 ± 0.59	09.0 ± 2.59	12.1 ±11.73	-4.01±0.02	21.35±0.08	2.47±0.06
TabZIP52	-02.6 ± 0.07	-19.0 ± 0.04	-06.2 ± 0.03	-03.0 ± 0.01	12.7 ± 0.02	03.8 ± 0.02	-3.92±0.03	19.01±0.03	10.12±0.02
TabZIP193.1	-39.7 ± 0.10	-09.6 ± 0.08	13.3 ± 0.08	-29.0 ± 0.09	24.0 ± 0.05	15.3 ± 0.00	-3.03±0.13	9.17±0.04	1.98±0.04
TabZIP120.3	-34.8 ± 0.04	-08.6 ± 0.04	11.2 ± 0.02	-27.3 ± 0.04	20.6 ± 0.11	12.4 ± 0.01	-5.53±0.03	8.68±0.07	1.19±0.02
TabZIP198.1	01.6 ± 0.05	-05.6 ± 0.10	05.9 ± 0.05	04.3 ± 0.05	15.3 ± 0.06	09.7 ± 0.04	-7.27±0.05	5.64±0.07	3.81±0.02
TabZIP203	-05.9 ± 0.04	-08.2 ± 0.23	08.6 ± 0.00	-02.7 ± 0.07	16.3 ± 0.02	08.0 ± 0.02	-6.43±0.03	8.21±0.12	-0.68±0.02
TabZIP209.1	05.9 ± 0.06	-07.4 ± 0.47	10.0 ± 0.04	12.6 ± 0.07	17.6 ± 0.05	12.6 ± 0.02	-5.24±0.03	7.50±0.27	2.61±0.02
TabZIP208	00.8 ± 0.15	-08.9 ± 0.04	04.1 ± 0.06	03.1 ± 0.01	17.2 ± 0.02	06.4 ± 0.07	-5.00±0.07	8.93±0.02	2.28±0.02
TabZIP212	07.9 ± 0.02	-10.2 ± 0.26	09.1 ± 0.05	10.2 ± 0.11	13.4 ± 0.04	11.6 ± 0.05	-5.47±0.07	10.23±0.14	2.48±0.02
TabZIP216	-02.5 ± 0.03	-12.9 ± 0.12	13.4 ± 0.03	08.1 ± 0.00	23.5 ± 0.02	15.6 ± 0.06	-0.82±0.02	12.93±0.07	2.20±0.04
TabZIP219.2	-36.6 ± 0.05	-08.0 ± 0.17	13.1 ± 0.14	-26.8 ± 0.02	23.0 ± 0.03	17.4 ± 0.08	-7.15±0.04	8.03±0.08	3.71±0.05
TabZIP8.2	-22. 5± 0.07	-06.4 ± 0.38	03.7 ± 0.03	-22.4 ± 0.02	08.7 ± 0.04	06.2 ± 0.04	-3.48±0.03	6.43±0.21	2.44±0.03
TabZIP224	-40.1 ± 0.16	-09.9 ± 0.09	17.1 ± 0.03	-28.5 ± 0.03	24.9 ± 0.02	018.1 ± 0.0	-6.79±0.06	9.95±0.09	0.99±0.03

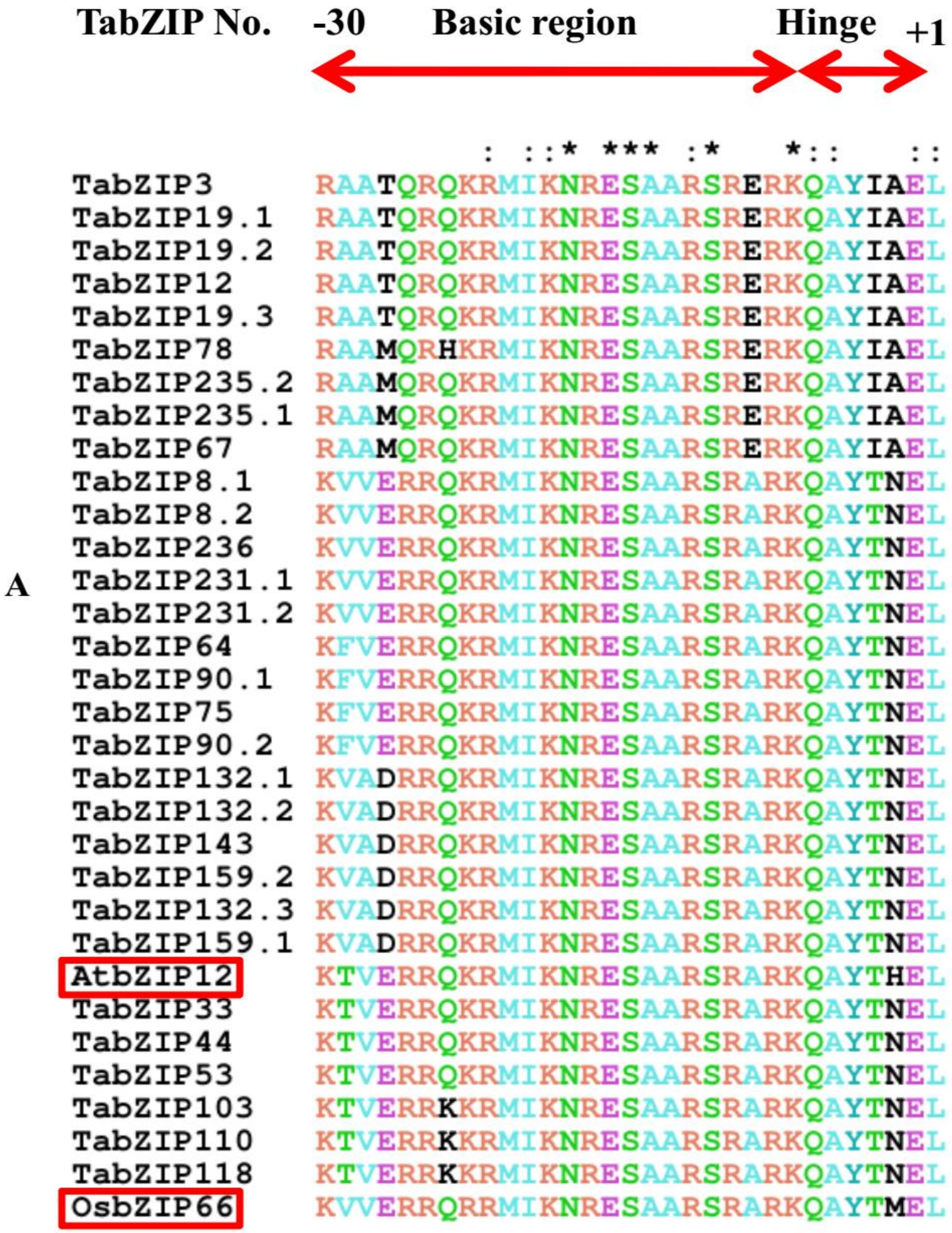
TabZIP No.
-30
Basic region
Hinge
+1
A
TabZIP3 RAATQRQKRMIKNRESAARSRERKQAYIAEL
TabZIP19.1 RAATQRQKRMIKNRESAARSRERKQAYIAEL
TabZIP19.2 RAATQRQKRMIKNRESAARSRERKQAYIAEL
TabZIP12 RAATQRQKRMIKNRESAARSRERKQAYIAEL
TabZIP19.3 RAATQRQKRMIKNRESAARSRERKQAYIAEL
TabZIP78 RAAMQRHKRMIKNRESAARSRERKQAYIAEL
TabZIP235.2 RAAMQRQKRMIKNRESAARSRERKQAYIAEL
TabZIP235.1 RAAMQRQKRMIKNRESAARSRERKQAYIAEL
TabZIP67 RAAMQRQKRMIKNRESAARSRERKQAYIAEL
TabZIP8.1 KVVERRQKRMIKNRESAARSRARKQAYTNEL
TabZIP8.2 KVVERRQKRMIKNRESAARSRARKQAYTNEL
TabZIP236 KVVERRQKRMIKNRESAARSRARKQAYTNEL
TabZIP231.1 KVVERRQKRMIKNRESAARSRARKQAYTNEL
TabZIP231.2 KVVERRQKRMIKNRESAARSRARKQAYTNEL
TabZIP64 KFVERRQKRMIKNRESAARSRARKQAYTNEL
TabZIP90.1 KFVERRQKRMIKNRESAARSRARKQAYTNEL
TabZIP75 KFVERRQKRMIKNRESAARSRARKQAYTNEL
TabZIP90.2 KFVERRQKRMIKNRESAARSRARKQAYTNEL
TabZIP132.1 KVADRRQKRMIKNRESAARSRARKQAYTNEL
TabZIP132.2 KVADRRQKRMIKNRESAARSRARKQAYTNEL
TabZIP143 KVADRRQKRMIKNRESAARSRARKQAYTNEL
TabZIP159.2 KVADRRQKRMIKNRESAARSRARKQAYTNEL
TabZIP132.3 KVADRRQKRMIKNRESAARSRARKQAYTNEL
TabZIP159.1 KVADRRQKRMIKNRESAARSRARKQAYTNEL
AtbZIP12 KTVERRQKRMIKNRESAARSRARKQAYTHEL
TabZIP33 KTVERRQKRMIKNRESAARSRARKQAYTNEL
TabZIP44 KTVERRQKRMIKNRESAARSRARKQAYTNEL
TabZIP53 KTVERRQKRMIKNRESAARSRARKQAYTNEL
TabZIP103 KTVERRKKRMIKNRESAARSRARKQAYTNEL
TabZIP110 KTVERRKKRMIKNRESAARSRARKQAYTNEL
TabZIP118 KTVERRKKRMIKNRESAARSRARKQAYTNEL
OsbZIP66 KVVERRQRRMIKNRESAARSRARKQAYTMEL

A

```
                     : ::* *** :*   *::   ::
ZmbZIP14       KVVERRQRRMIKNRESAARSRARKQAYTMEL
TabZIP129.1    KVVERRQRRMIKNRESAARSRARKQAYTMEL
TabZIP226.3    KVVERRQRRMIKNRESAARSRARKQAYTMEL
TabZIP129.2    KVVERRQRRMIKNRESAARSRARKQAYTMEL
TabZIP129.3    KVVERRQRRMIKNRESAARSRARKQAYTMEL
TabZIP170.1    KVVERRQRRMIKNRESAARSRARKQAYTMEL
TabZIP170.2    KVVERRQRRMIKNRESAARSRARKQAYTMEL
TabZIP226.1    KVVERRQRRMIKNRESAARSRARKQAYTMEL
TabZIP226.2    KVVERRQRRMIKNRESAARSRARKQAYTMEL
TabZIP175.1    KVVERRQRRMIKNRESAARSRQRKQSYMMEL
TabZIP175.2    KVVERRQRRMIKNRESAARSRQRKQSYMMEL
TabZIP175.3    KVVERRQRRMIKNRESAARSRQRKQSYMMEL
TabZIP183.1    KVVERRQRRMIKNRESAARSRQRKQSYMMEL
TabZIP183.2    KVVERRQRRMIKNRESAARSRQRKQSYMMEL
TabZIP190.1    KVVERRQRRMIKNRESAARSRQRKQSYMMEL
TabZIP190.2    KVVERRQRRMIKNRESAARSRQRKQSYMMEL
TabZIP190.3    KVVERRQRRMIKNRESAARSRQRKQSYMMEL
TabZIP204.1    KVVERRQRRMIKNRESAARSRQRKQAYIMEL
TabZIP204.2    KVVERRQRRMIKNRESAARSRQRKQAYIMEL
TabZIP225      KVVERRQRRMIKNRESAARSRQRKQAYIMEL
TabZIP211      KVVERRQRRMIKNRESAARSRQRKQAYIMEL
TabZIP74       RSIERRHRRMIKNRESAARSRARKQAYTVEL
TabZIP86       RSIERRHRRMIKNRESAARSRARKQAYTVEL
TabZIP57       RSIERRHRRMIKNRESAARSRARKQAYTVEL
TabZIP73       RSIERRHRRMIKNRESAARSRARKQAYTVEL
TabZIP84.1     RSIECRHRRMIKNGESAARSRARKQAFTVEL
TabZIP84.2     RSIECRHRRMIKNGESAARSRARKQAFTVEL
TabZIP71       RSIERRHRRMIKNGESAARSRARKQAFTVEL
TabZIP72       RSIERRHRRMIKNRESAARSRARKQAYTVEL
HvbZIP7        RSIERRHRRMIKNRESAARSRARKQAYTVEL
TabZIP63       RSIERRHHRMIKNRESATQSRGWKQAYTKEL
TabZIP66       GSIERRHHHMIKNHESAAQSCGRKQAYTKEL
```

A

```
                    :  ::* *** :*   *::   ::
TabZIP62     RSIECRHHRMIKNHESAAQSRGRKQAYTKEL
TabZIP87     RSIERCHHRMIKNRESAGQSRARKQAYTVEL
TabZIP25     VGGDRRQRRMIKNRESAARSRARKQAYTNEL
TabZIP49     VGGDRRQRRMIKNRESAARSRARKQAYTNEL
TabZIP41     VGGDRRQRRMIKNRESAARSRARKQAYTNEL
TabZIP27     AGGDRRQRRMIKNRESAARSRARKQACTNEM
TabZIP46     AGDDRRQRRMIKNRESAARSRARKQACTNEM
TabZIP47     VGGDRRQRRMVKNRESAARSRARKQAHTNEM
TabZIP26     VGGDRRQRRMVKNRESAARSRARKQAHTNEM
TabZIP40     VGGDRRQRRMVKNRESAARSRARKQAHTNEM
TabZIP115    AAVDR-QRRMIKNRESAARSRARKQAYTNEL
TabZIP134    AAVDR-QRRMIKNRESAARSRARKQAYTNEL
TabZIP105    AAADR-QRRMIKNRESAARSRARKQAYTNEL
TabZIP48.1   D-DGHKSVRAMKNRESALRSRARKRAYTQEL
TabZIP48.2   D-DGHKSVRAMKNRESALRSRARKRAYTQEL
TabZIP28     D-DGHKSVRAMKNRESALRSRARKRAYTQEL
TabZIP37     D-DGHKSIRAMKNRESALRSRARKRAYTQEL
TabZIP197    G-EDPRTIRMMRNRESALRSRARKRAYVEEL
TabZIP234    G-EDPRTIRMMRNRESALRSRARKRAYVEEL
TabZIP208    GGDDRRTVRMMRNRESALRSRARKRAYVEEL
TabZIP128    P-VDRRKKRMIKNRESASRSRARKQAHVTQI
TabZIP165    P-VDRRKKRMIKNRESASRSRARKQAHVTQI
TabZIP149    P-VDRRKKRMIKNRESASRSRARKQAHVTQI
```

B

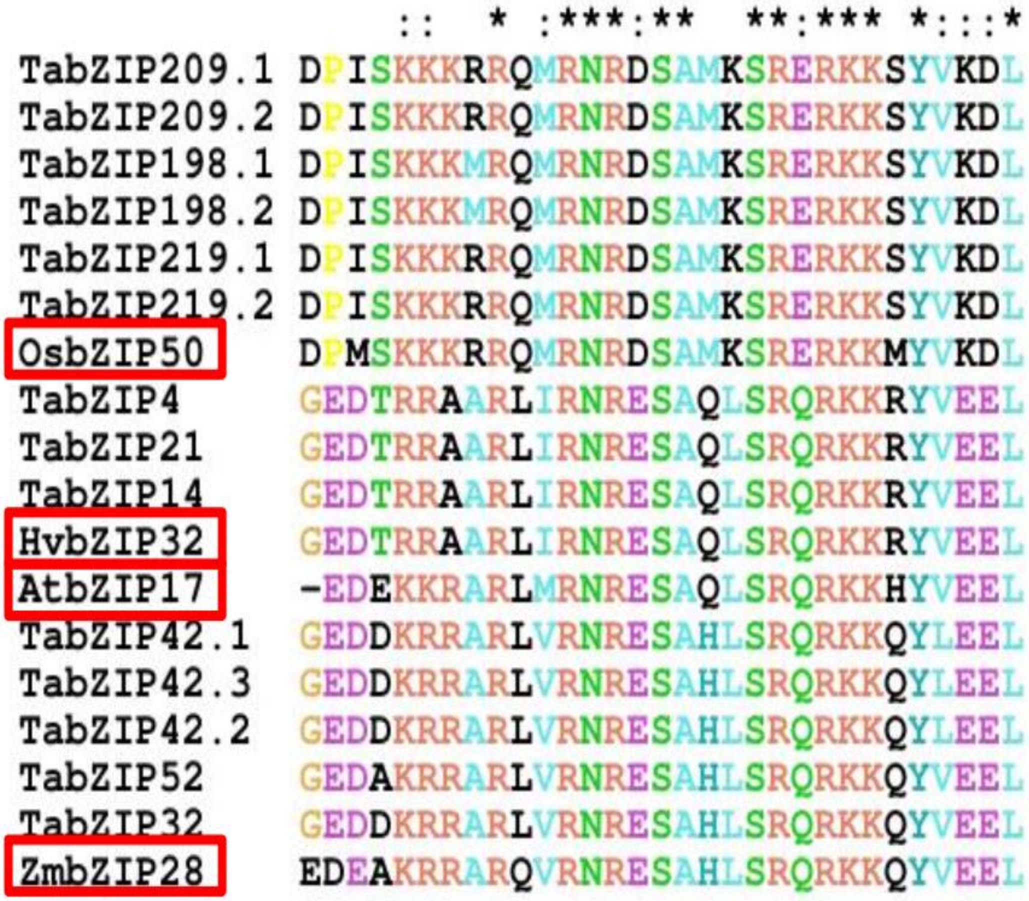
:: * :***:** **:*** *:::*
TabZIP209.1 DPISKKKRRQMRNRDSAMKSRERKKSYVKDL
TabZIP209.2 DPISKKKRRQMRNRDSAMKSRERKKSYVKDL
TabZIP198.1 DPISKKKMRQMRNRDSAMKSRERKKSYVKDL
TabZIP198.2 DPISKKKMRQMRNRDSAMKSRERKKSYVKDL
TabZIP219.1 DPISKKKRRQMRNRDSAMKSRERKKSYVKDL
TabZIP219.2 DPISKKKRRQMRNRDSAMKSRERKKSYVKDL
OsbZIP50 DPMSKKKRRQMRNRDSAMKSRERKKMYVKDL
TabZIP4 GEDTRRAARLIRNRESAQLSRQRKKRYVEEL
TabZIP21 GEDTRRAARLIRNRESAQLSRQRKKRYVEEL
TabZIP14 GEDTRRAARLIRNRESAQLSRQRKKRYVEEL
HvbZIP32 GEDTRRAARLIRNRESAQLSRQRKKRYVEEL
AtbZIP17 -EDEKKRARLMRNRESAQLSRQRKKHYVEEL
TabZIP42.1 GEDDKRRARLVRNRESAHLSRQRKKQYLEEL
TabZIP42.3 GEDDKRRARLVRNRESAHLSRQRKKQYLEEL
TabZIP42.2 GEDDKRRARLVRNRESAHLSRQRKKQYLEEL
TabZIP52 GEDAKRRARLVRNRESAHLSRQRKKQYVEEL
TabZIP32 GEDDKRRARLVRNRESAHLSRQRKKQYVEEL
ZmbZIP28 EDEAKRRARQVRNRESAHLSRQRKKQYVEEL

C

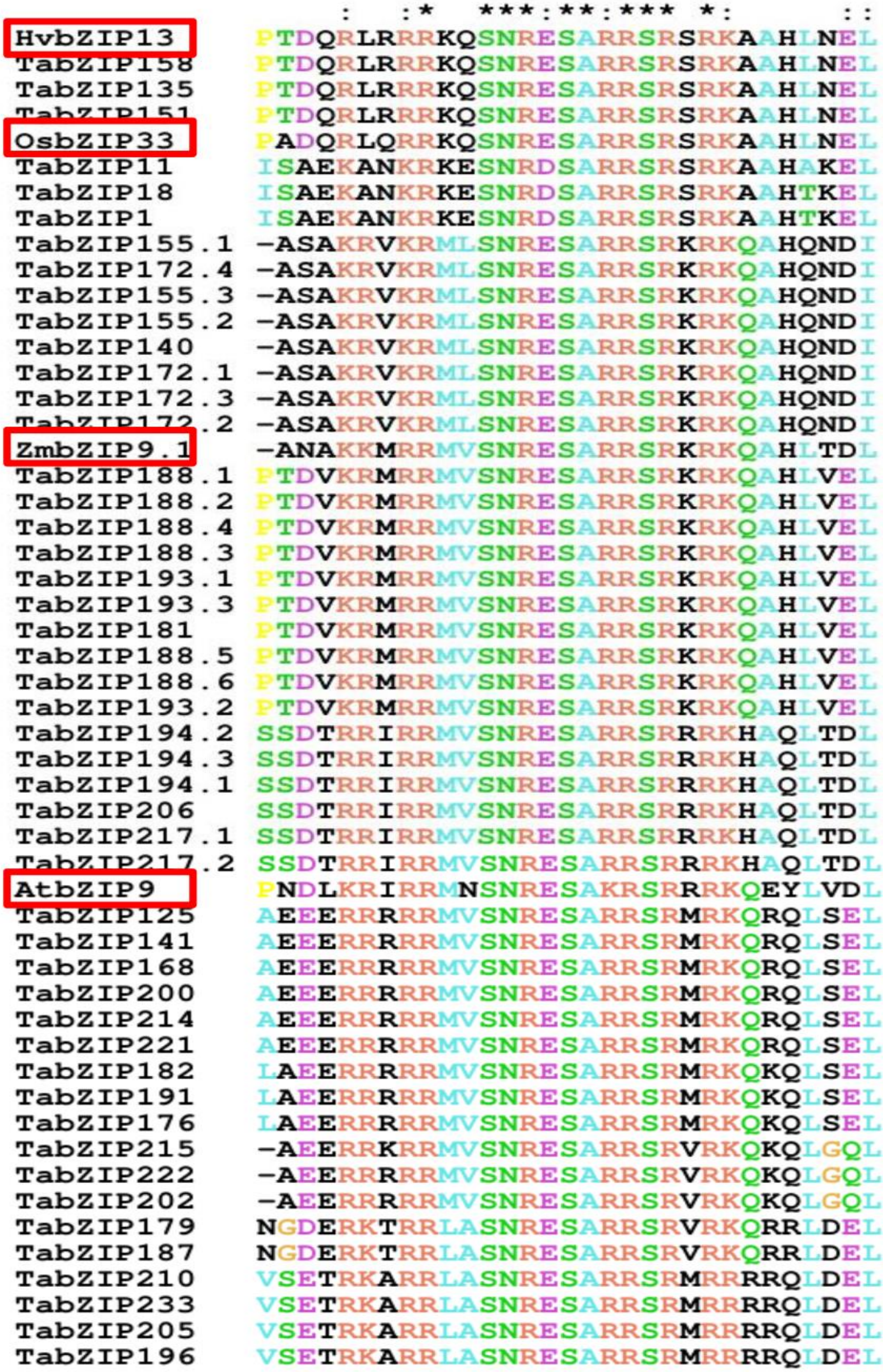

: :* ***:**:*** *: ::
HvbZIP13 PTDQRLRRRKQSNRESARRSRSRKAAHLNEL
TabZIP158 PTDQRLRRRKQSNRESARRSRSRKAAHLNEL
TabZIP135 PTDQRLRRRKQSNRESARRSRSRKAAHLNEL
TabZIP151 PTDQRLRRRKQSNRESARRSRSRKAAHLNEL
OsbZIP33 PADQRLQRRKQSNRESARRSRSRKAAHLNEL
TabZIP11 ISAEKANKRKESNRDSARRSRSRKAAHAKEL
TabZIP18 ISAEKANKRKESNRDSARRSRSRKAAHTKEL
TabZIP1 ISAEKANKRKESNRDSARRSRSRKAAHTKEL
TabZIP155.1 -ASAKRVKRMLSNRESARRSRKRKQAHQNDI
TabZIP172.4 -ASAKRVKRMLSNRESARRSRKRKQAHQNDI
TabZIP155.3 -ASAKRVKRMLSNRESARRSRKRKQAHQNDI
TabZIP155.2 -ASAKRVKRMLSNRESARRSRKRKQAHQNDI
TabZIP140 -ASAKRVKRMLSNRESARRSRKRKQAHQNDI
TabZIP172.1 -ASAKRVKRMLSNRESARRSRKRKQAHQNDI
TabZIP172.3 -ASAKRVKRMLSNRESARRSRKRKQAHQNDI
TabZIP172.2 -ASAKRVKRMLSNRESARRSRKRKQAHQNDI
ZmbZIP9.1 -ANAKKMRRMVSNRESARRSRKRKQAHLTDL
TabZIP188.1 PTDVKRMRRMVSNRESARRSRKRKQAHLVEL
TabZIP188.2 PTDVKRMRRMVSNRESARRSRKRKQAHLVEL
TabZIP188.4 PTDVKRMRRMVSNRESARRSRKRKQAHLVEL
TabZIP188.3 PTDVKRMRRMVSNRESARRSRKRKQAHLVEL
TabZIP193.1 PTDVKRMRRMVSNRESARRSRKRKQAHLVEL
TabZIP193.3 PTDVKRMRRMVSNRESARRSRKRKQAHLVEL
TabZIP181 PTDVKRMRRMVSNRESARRSRKRKQAHLVEL
TabZIP188.5 PTDVKRMRRMVSNRESARRSRKRKQAHLVEL
TabZIP188.6 PTDVKRMRRMVSNRESARRSRKRKQAHLVEL
TabZIP193.2 PTDVKRMRRMVSNRESARRSRKRKQAHLVEL
TabZIP194.2 SSDTRRIRRMVSNRESARRSRRRKHAQLTDL
TabZIP194.3 SSDTRRIRRMVSNRESARRSRRRKHAQLTDL
TabZIP194.1 SSDTRRIRRMVSNRESARRSRRRKHAQLTDL
TabZIP206 SSDTRRIRRMVSNRESARRSRRRKHAQLTDL
TabZIP217.1 SSDTRRIRRMVSNRESARRSRRRKHAQLTDL
TabZIP217.2 SSDTRRIRRMVSNRESARRSRRRKHAQLTDL
AtbZIP9 PNDLKRIRRMNSNRESAKRSRRRKQEYLVDL
TabZIP125 AEEERRRRRMVSNRESARRSRMRKQRQLSEL
TabZIP141 AEEERRRRRMVSNRESARRSRMRKQRQLSEL
TabZIP168 AEEERRRRRMVSNRESARRSRMRKQRQLSEL
TabZIP200 AEEERRRRRMVSNRESARRSRMRKQRQLSEL
TabZIP214 AEEERRRRRMVSNRESARRSRMRKQRQLSEL
TabZIP221 AEEERRRRRMVSNRESARRSRMRKQRQLSEL
TabZIP182 LAEERRRRRMVSNRESARRSRMRKQKQLSEL
TabZIP191 LAEERRRRRMVSNRESARRSRMRKQKQLSEL
TabZIP176 LAEERRRRRMVSNRESARRSRMRKQKQLSEL
TabZIP215 -AEERRKRRMVSNRESARRSRVRKQKQLGQL
TabZIP222 -AEERRRRRMVSNRESARRSRVRKQKQLGQL
TabZIP202 -AEERRRRRMVSNRESARRSRVRKQKQLGQL
TabZIP179 NGDERKTRRLASNRESARRSRVRKQRRLDEL
TabZIP187 NGDERKTRRLASNRESARRSRVRKQRRLDEL
TabZIP210 VSETRKARRLASNRESARRSRMRRRRQLDEL
TabZIP233 VSETRKARRLASNRESARRSRMRRRRQLDEL
TabZIP205 VSETRKARRLASNRESARRSRMRRRRQLDEL
TabZIP196 VSETRKARRLASNRESARRSRMRRRRQLDEL

```
                              **********::**:*****: :*
   TabZIP77.1     VDSK-TERRLAQNREAAKKSRLRKKAYVQNL
   TabZIP77.7     VDSK-TERRLAQNREAAKKSRLRKKAYVQNL
   TabZIP59.6     VDSK-TERRLAQNREAAKKSRLRKKAYVQNL
   TabZIP91.1     VDSK-TERRLAQNREAAKKSRLRKKAYVQNL
   TabZIP77.3     VDSK-TERRLAQNREAAKKSRLRKKAYVQNL
   TabZIP77.5     VDSK-TERRLAQNREAAKKSRLRKKAYVQNL
   TabZIP77.4     VDSK-TERRLAQNREAAKKSRLRKKAYVQNL
   TabZIP77.6     -----TERRLAQNREAAKKSRLRKKAYVQNL
   TabZIP77.2     VDSK-TERRLAQNREAAKKSRLRKKAYVQNL
   TabZIP91.3     VDSK-TERRLAQNREAAKKSRLRKKAYVQNL
   TabZIP91.2     VDSK-TERRLAQNREAAKKSRLRKKAYVQNL
   TabZIP59.4     VDSK-TERRLAQNREAAKKSRLRKKAYVQNL
   TabZIP59.5     VDSK-TERRLAQNREAAKKSRLRKKAYVQNL
   TabZIP77.8     VDSK-TERRLAQNREAAKKSRLRKKAYVQNL
   TabZIP59.1     VDSK-TERRLAQNREAAKKSRLRKKAYVQNL
D  TabZIP59.3     VDSK-TERRLAQNREAAKKSRLRKKAYVQNL
   TabZIP91.4     VDSK-TERRLAQNREAAKKSRLRKKAYVQNL
   TabZIP91.5     VDSK-TERRLAQNREAAKKSRLRKKAYVQNL
   TabZIP59.2     VDSK-TERRLAQNREAAKKSRLRKKAYVQNL
   OsbZIP11       VDAK-TERRLAQNREAARKSRLRKKAYVQNL
   HvbZIP43.1     VDPK-TERRLAQNREAARKSRLRKKAYVQQL
   TabZIP20.1     VDPK-TERRLAQNREAARKSRLRKKAYVQQL
   TabZIP13.4     VDPK-TERRLAQNREAARKSRLRKKAYVQQL
   TabZIP13.5     VDPK-TERRLAQNREAARKSRLRKKAYVQQL
   TabZIP13.3     VDPK-TERRLAQNREAARKSRLRKKAYVQQL
   TabZIP13.2     VDPK-TERRLAQNREAARKSRLRKKAYVQQL
   TabZIP13.1     VDPK-TERRLAQNREAARKSRLRKKAYVQQL
   TabZIP6.1      VDPK-TERRLAQNREAARKSRLRKKAYVQQL
   TabZIP6.2      VDPK-TERRLAQNREAARKSRLRKKAYVQQL
   TabZIP6.3      VDPK-TERRLAQNREAARKSRLRKKAYVQQL
   TabZIP20.2     VDPK-TERRLAQNREAARKSRLRKKAYVQQL
   TabZIP96.1     LDPK-TTRRLAQNREAARKSRLRKKAYIQQL
```

D

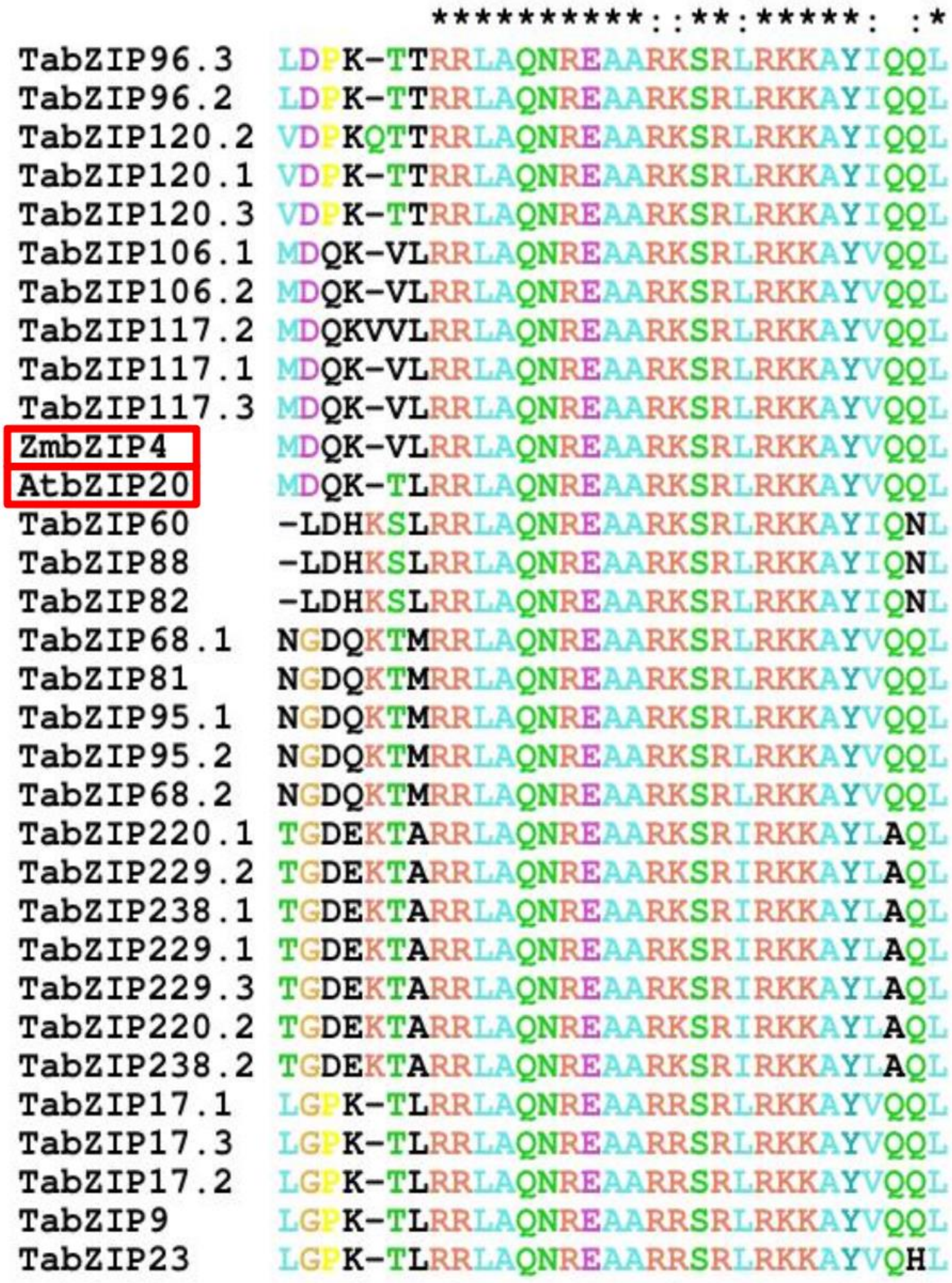
**********::**:*****: :*
TabZIP96.3 LDPK-TTRRLAQNREAARKSRLRKKAYIQQL
TabZIP96.2 LDPK-TTRRLAQNREAARKSRLRKKAYIQQL
TabZIP120.2 VDPKQTTRRLAQNREAARKSRLRKKAYIQQL
TabZIP120.1 VDPK-TTRRLAQNREAARKSRLRKKAYIQQL
TabZIP120.3 VDPK-TTRRLAQNREAARKSRLRKKAYIQQL
TabZIP106.1 MDQK-VLRRLAQNREAARKSRLRKKAYVQQL
TabZIP106.2 MDQK-VLRRLAQNREAARKSRLRKKAYVQQL
TabZIP117.2 MDQKVVLRRLAQNREAARKSRLRKKAYVQQL
TabZIP117.1 MDQK-VLRRLAQNREAARKSRLRKKAYVQQL
TabZIP117.3 MDQK-VLRRLAQNREAARKSRLRKKAYVQQL
ZmbZIP4 MDQK-VLRRLAQNREAARKSRLRKKAYVQQL
AtbZIP20 MDQK-TLRRLAQNREAARKSRLRKKAYVQQL
TabZIP60 -LDHKSLRRLAQNREAARKSRLRKKAYIQNL
TabZIP88 -LDHKSLRRLAQNREAARKSRLRKKAYIQNL
TabZIP82 -LDHKSLRRLAQNREAARKSRLRKKAYIQNL
TabZIP68.1 NGDQKTMRRLAQNREAARKSRLRKKAYVQQL
TabZIP81 NGDQKTMRRLAQNREAARKSRLRKKAYVQQL
TabZIP95.1 NGDQKTMRRLAQNREAARKSRLRKKAYVQQL
TabZIP95.2 NGDQKTMRRLAQNREAARKSRLRKKAYVQQL
TabZIP68.2 NGDQKTMRRLAQNREAARKSRLRKKAYVQQL
TabZIP220.1 TGDEKTARRLAQNREAARKSRIRKKAYLAQL
TabZIP229.2 TGDEKTARRLAQNREAARKSRIRKKAYLAQL
TabZIP238.1 TGDEKTARRLAQNREAARKSRIRKKAYLAQL
TabZIP229.1 TGDEKTARRLAQNREAARKSRIRKKAYLAQL
TabZIP229.3 TGDEKTARRLAQNREAARKSRIRKKAYLAQL
TabZIP220.2 TGDEKTARRLAQNREAARKSRIRKKAYLAQL
TabZIP238.2 TGDEKTARRLAQNREAARKSRIRKKAYLAQL
TabZIP17.1 LGPK-TLRRLAQNREAARRSRLRKKAYVQQL
TabZIP17.3 LGPK-TLRRLAQNREAARRSRLRKKAYVQQL
TabZIP17.2 LGPK-TLRRLAQNREAARRSRLRKKAYVQQL
TabZIP9 LGPK-TLRRLAQNREAARRSRLRKKAYVQQL
TabZIP23 LGPK-TLRRLAQNREAARRSRLRKKAYVQHL

```
                           **********::**:*****: :*
TabZIP30.1     -PKEKVMRRLAQNREAARKSRLRKKAYIQQL
TabZIP30.2     -PKEKVMRRLAQNREAARKSRLRKKAYIQQL
TabZIP38.1     -PKEKVMRRLAQNREAARKSRLRKKAYIQQL
TabZIP38.4     -PKEKVMRRLAQNREAARKSRLRKKAYIQQL
TabZIP38.3     -PKEKVMRRLAQNREAARKSRLRKKAYIQQL
TabZIP50.1     -PKEKVMRRLAQNREAARKSRLRKKAYIQQL
TabZIP50.3     -PKEKVMRRLAQNREAARKSRLRKKAYIQQL
TabZIP38.2     -PKEKVMRRLAQNREAARKSRLRKKAYIQQL
TabZIP50.2     -PKEKVMRRLAQNREAARKSRLRKKAYIQQL
TabZIP123      ERDPKTLRRLAQNREAARKSRLRKKAYIQQL
TabZIP164      ERDPKTLRRLAQNREAARKSRLRKKAYIQQL
TabZIP152      ERDPKTLRRLAQNREAARKSRLRKKAYIQQL
TabZIP167.1    TQDPKTLRRLAQNREAARKSRLRKKAYIQQL
TabZIP167.2    TQDPKTLRRLAQNREAARKSRLRKKAYIQQL
TabZIP126      TQDPKTLRRLAQNREAARKSRLRKKAYIQQL
TabZIP142.1    TQDPKTLRRLAQNREAARKSRLRKKAYIQQL
TabZIP142.2    TQDPKTLRRLAQNREAARKSRLRKKAYIQQL
TabZIP184.1    TPDAKTLRRLAQNREAARKSRLRKKAYIQNL
TabZIP184.2    TPDAKTLRRLAQNREAARKSRLRKKAYIQNL
TabZIP228.1    TPDAKTLRRLAQNREAARKSRLRKKAYIQNL
TabZIP228.3    TPDAKTLRRLAQNREAARKSRLRKKAYIQNL
TabZIP228.2    TPDAKTLRRLAQNREAARKSRLRKKAYIQNL
TabZIP177      TPDAKTLRRLAQNREAARKSRLRKKAYIQNL
```

D

E

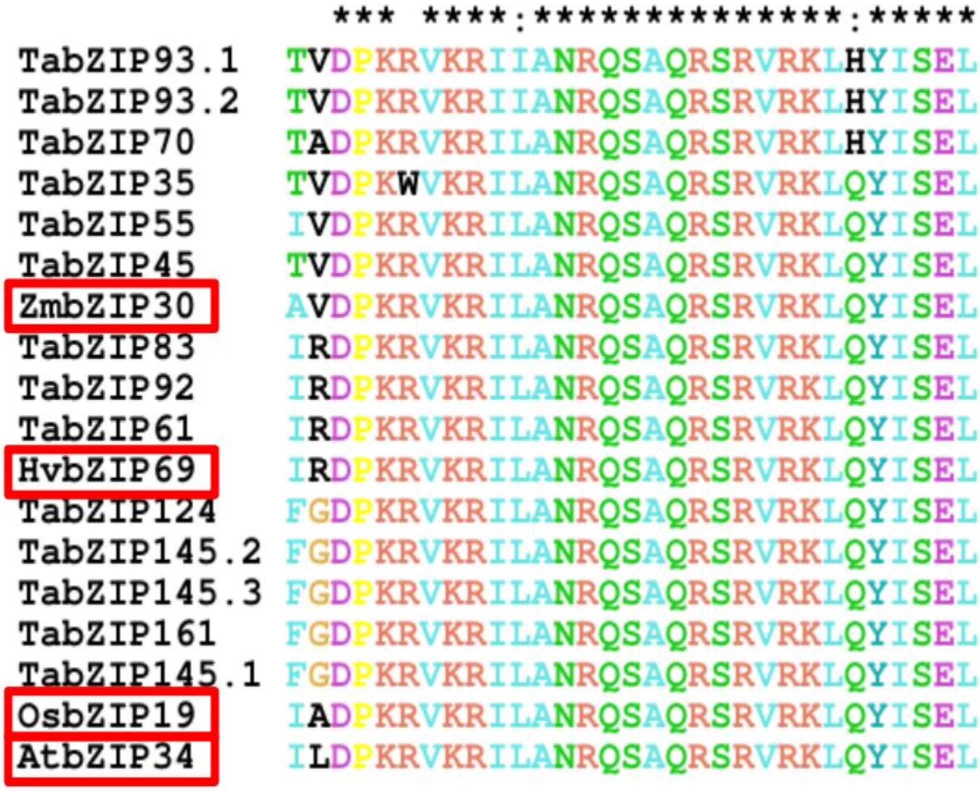
*** ****:**************:*****
TabZIP93.1 TVDPKRVKRIIANRQSAQRSRVRKLHYISEL
TabZIP93.2 TVDPKRVKRIIANRQSAQRSRVRKLHYISEL
TabZIP70 TADPKRVKRILANRQSAQRSRVRKLHYISEL
TabZIP35 TVDPKWVKRILANRQSAQRSRVRKLQYISEL
TabZIP55 IVDPKRVKRILANRQSAQRSRVRKLQYISEL
TabZIP45 TVDPKRVKRILANRQSAQRSRVRKLQYISEL
ZmbZIP30 AVDPKRVKRILANRQSAQRSRVRKLQYISEL
TabZIP83 IRDPKRVKRILANRQSAQRSRVRKLQYISEL
TabZIP92 IRDPKRVKRILANRQSAQRSRVRKLQYISEL
TabZIP61 IRDPKRVKRILANRQSAQRSRVRKLQYISEL
HvbZIP69 IRDPKRVKRILANRQSAQRSRVRKLQYISEL
TabZIP124 FGDPKRVKRILANRQSAQRSRVRKLQYISEL
TabZIP145.2 FGDPKRVKRILANRQSAQRSRVRKLQYISEL
TabZIP145.3 FGDPKRVKRILANRQSAQRSRVRKLQYISEL
TabZIP161 FGDPKRVKRILANRQSAQRSRVRKLQYISEL
TabZIP145.1 FGDPKRVKRILANRQSAQRSRVRKLQYISEL
OsbZIP19 IADPKRVKRILANRQSAQRSRVRKLQYISEL
AtbZIP34 ILDPKRVKRILANRQSAQRSRVRKLQYISEL

F

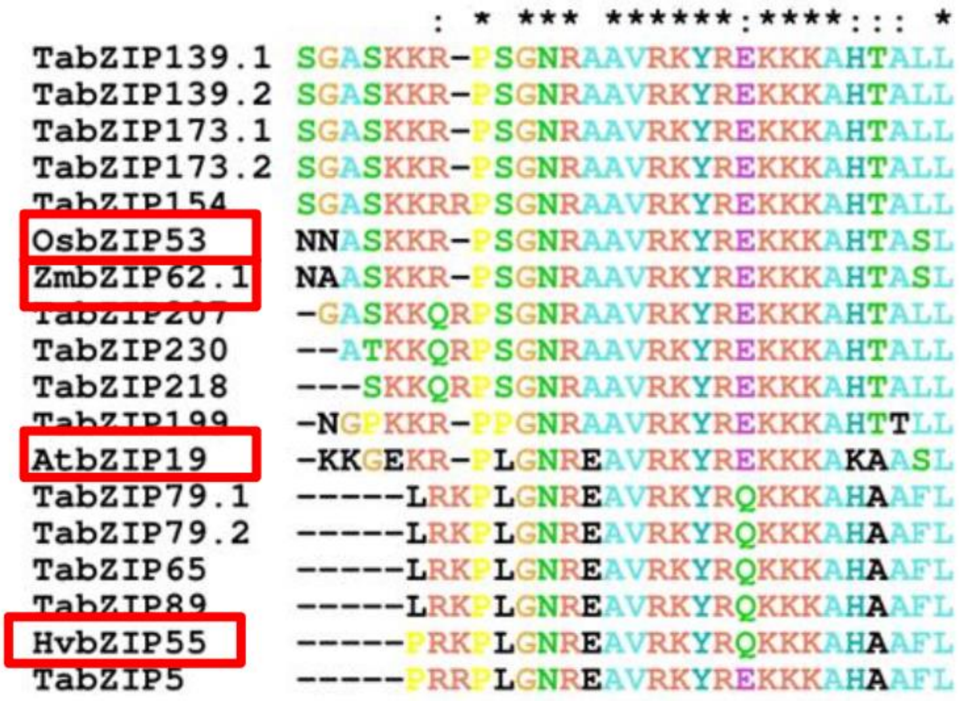
: * *** ******:****::: *
TabZIP139.1 SGASKKR-PSGNRAAVRKYREKKKAHTALL
TabZIP139.2 SGASKKR-PSGNRAAVRKYREKKKAHTALL
TabZIP173.1 SGASKKR-PSGNRAAVRKYREKKKAHTALL
TabZIP173.2 SGASKKR-PSGNRAAVRKYREKKKAHTALL
TabZIP154 SGASKKRRPSGNRAAVRKYREKKKAHTALL
OsbZIP53 NNASKKR-PSGNRAAVRKYREKKKAHTASL
ZmbZIP62.1 NAASKKR-PSGNRAAVRKYREKKKAHTASL
TabZIP207 -GASKKQRPSGNRAAVRKYREKKKAHTALL
TabZIP230 --ATKKQRPSGNRAAVRKYREKKKAHTALL
TabZIP218 ---SKKQRPSGNRAAVRKYREKKKAHTALL
TabZIP199 -NGPKKR-PPGNRAAVRKYREKKKAHTTLL
AtbZIP19 -KKGEKR-PLGNREAVRKYREKKKAKAASL
TabZIP79.1 -----LRKPLGNREAVRKYRQKKKAHAAFL
TabZIP79.2 -----LRKPLGNREAVRKYRQKKKAHAAFL
TabZIP65 -----LRKPLGNREAVRKYRQKKKAHAAFL
TabZIP89 -----LRKPLGNREAVRKYRQKKKAHAAFL
HvbZIP55 -----PRKPLGNREAVRKYRQKKKAHAAFL
TabZIP5 -----PRRPLGNREAVRKYREKKKAHAAFL

```
               :* *:::** ***:*********      :
TabZIP7.2     EREVKREKRKQSNRESARRSRLRKQAETEEL
TabZIP232.1   EREVKREKRKQSNRESARRSRLRKQAETEEL
TabZIP16.1    EREVKREKRKQSNRESARRSRLRKQAETEEL
TabZIP16.2    EREVKREKRKQSNRESARRSRLRKQAETEEL
TabZIP232.2   EREVKREKRKQSNRESARRSRLRKQVYCVGL
OsbZIP5       DKESKRERRKQSNRESARRSRLRKQAETEEL
TabZIP178.1   ERELKKQKRKLSNRESARRSRLRKQAECEEL
TabZIP178.3   ERELKKQKRKLSNRESARRSRLRKQAECEEL
TabZIP178.2   ERELKKQKRKLSNRESARRSRLRKQAECEEL
TabZIP237.2   ERELKKQKRKLSNRESARRSRLRKQAECEEL
TabZIP237.3   ERELKKQKRKLSNRESARRSRLRKQAECEEL
TabZIP237.1   ERELKKQKRKLSNRESARRSRLRKQAECEEL
TabZIP189.2   ERELKKQKRKLSNRESARRSRLRKQAECEEL
TabZIP189.3   ERELKKQKRKLSNRESARRSRLRKQAECEEL
TabZIP189.1   ERELKKQKRKLSNRESARRSRLRKQAECEEL
TabZIP162     ERELKRQKRKQSNRDSARRSRLRKQAECEEL
TabZIP227     ERELKRQKRKQSNRDSARRSRLRKQAECEEL
HvbZIP21.1    EREVKKQKRKQSNRESARRSRLRKQAEWEEV
TabZIP114     EREVKKQKRKQSNRESARRSRLRKQAEWEEV
TabZIP101.1   EREVKKQKRKQSNRESARRSRLRKQAEWEEV
TabZIP108     EREVKKQKRKQSNRESARRSRLRKQAEWEEV
TabZIP101.2   EREVKKQKRKQSNRESARRSRLRKQAEWEEV
ZmbZIP2       GRELKRQKRKQSNRESARRSRLRKQAEWEEV
AtbZIP41      ERELKRQKRKQSNRESARRSRLRKQAECEQL
TabZIP31.1    EREIKRERRKQSNRESARRSRLRKQQECEEL
TabZIP31.2    EREIKRERRKQSNRESARRSRLRKQQECEEL
TabZIP54.3    EREIKRERRKQSNRESARRSRLRKQQECEEL
TabZIP54.4    EREIKRERRKQSNRESARRSRLRKQQECEEL
TabZIP54.2    EREIKRERRKQSNRESARRSRLRKQQECEEL
TabZIP54.5    EREIKRERRKQSNRESARRSRLRKQQECEEL
TabZIP31.4    EREIKRERRKQSNRESARRSRLRK-QVVMEL
TabZIP54.6    EREIKRERRKQSNRESARRSRLRK-QVVMEI
TabZIP136.1   ERELKRERRKQSNRESARRSRLRKQQECEEL
TabZIP136.2   ERELKRERRKQSNRESARRSRLRKQQECEEL
TabZIP171.1   ERELKRERRKQSNRESARRSRLRKQQECEEL
TabZIP171.2   ERELKRERRKQSNRESARRSRLRKQQECEEL
```

G

H

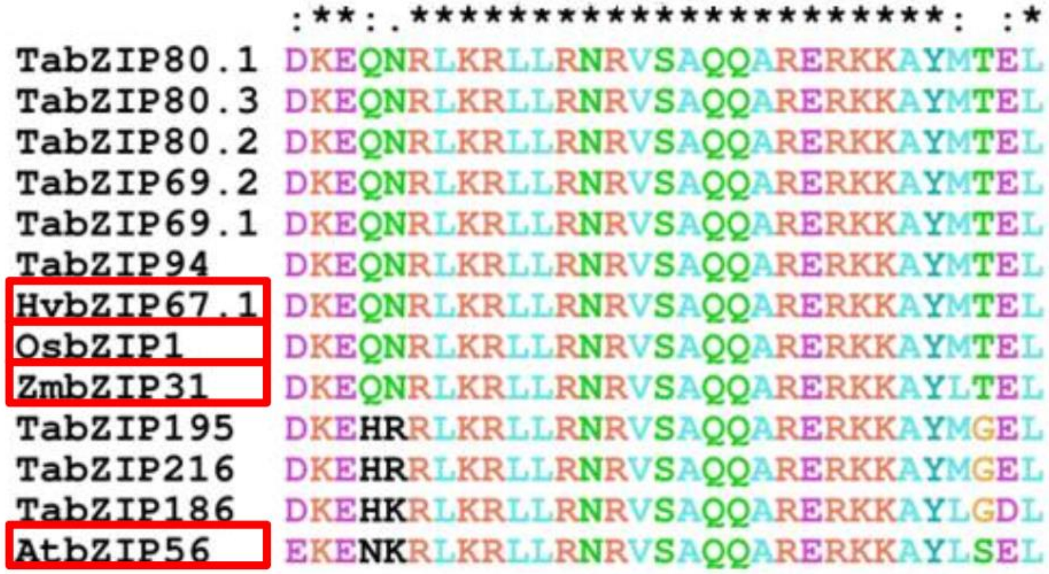
:**:.**********************: :*
TabZIP80.1 DKEQNRLKRLLRNRVSAQQARERKKAYMTEL
TabZIP80.3 DKEQNRLKRLLRNRVSAQQARERKKAYMTEL
TabZIP80.2 DKEQNRLKRLLRNRVSAQQARERKKAYMTEL
TabZIP69.2 DKEQNRLKRLLRNRVSAQQARERKKAYMTEL
TabZIP69.1 DKEQNRLKRLLRNRVSAQQARERKKAYMTEL
TabZIP94 DKEQNRLKRLLRNRVSAQQARERKKAYMTEL
HvbZIP67.1 DKEQNRLKRLLRNRVSAQQARERKKAYMTEL
OsbZIP1 DKEQNRLKRLLRNRVSAQQARERKKAYMTEL
ZmbZIP31 DKEQNRLKRLLRNRVSAQQARERKKAYLTEL
TabZIP195 DKEHRRLKRLLRNRVSAQQARERKKAYMGEL
TabZIP216 DKEHRRLKRLLRNRVSAQQARERKKAYMGEL
TabZIP186 DKEHKRLKRLLRNRVSAQQARERKKAYLGDL
AtbZIP56 EKENKRLKRLLRNRVSAQQARERKKAYLSEL

```
                 ***:.:*:  ** ***:****: :*  **
    TabZIP147.1  LVDPKRAKRIWANRQSAARSKERKMRYIGEL
    TabZIP147.2  LVDPKRAKRIWANRQSAARSKERKMRYIGEL
    TabZIP137    LVDPKRAKRIWANRQSAARSKERKMRYIGEL
    TabZIP157.1  LVDPKRAKRIWANRQSAARSKERKMRYIGEL
    TabZIP157.3  LVDPKRAKRIWANRQSAARSKERKMRYIGEL
    TabZIP157.2  LVDPKRAKRIWANRQSAARSKERKMRYIGEL
    TabZIP157.4  LVDPKRAKRIWANRQSAARSKERKMRYIGEL
    TabZIP203    LVDPKKAKRIWANRQSAARSKERKMRYISEL
    TabZIP224    LVDPKKAKRIWANRQSAARSKERKMRYISEL
    TabZIP213    LVDPKKAKRIWANRQSAARSKERKMRYISEL
    AtbZIP59     LIDPKRAKRIWANRQSAARSKERKTRYIFEL
    TabZIP29.1   LVDPKRAKRIMANRQSAARSKERKMRYIAEL
    TabZIP29.2   LVDPKRAKRIMANRQSAARSKERKMRYIAEL
I   TabZIP36     LVDPKRAKRIMANRQSAARSKERKMRYIAEL
    TabZIP51     LVDPKRAKRIMANRQSAARSKERKMRYIAEL
    HvbZIP3.1    LVDPKRAKRIMANRQSAARSKERKMRYIAEL
    TabZIP56.1   SVDPKRAKRILANRQSAARSKERKARYITEL
    TabZIP56.3   SVDPKRAKRILANRQSAARSKERKARYITEL
    TabZIP56.2   SVDPKRAKRILANRQSAARSKERKARYITEL
    TabZIP34.1   SVDPKRAKRILANRQSAARSKERKARYITEL
    TabZIP34.3   SVDPKRAKRILANRQSAARSKERKARYITEL
    TabZIP43.1   SVDPKRAKRILANRQSAARSKERKARYITEL
    TabZIP43.2   SVDPKRAKRILANRQSAARSKERKARYITEL
    TabZIP34.2   SVDPKRAKRILANRQSAARSKERKARYITEL
    TabZIP104    AIDPKRAKRILANRQSAARSKERKARYMTEL
    TabZIP113    AIDPKRAKRILANRQSAARSKERKARYMTEL
    TabZIP100    AIDPKRAKRILANRQSAARSKERKARYMTEL
    OsbZIP30     AIDPKRAKRILANRQSAARSKERKARYITEL
    TabZIP97     LLDPKRAKRILANRQSAARSKERKIKYTGEL
    TabZIP119    LLDPKRAKRILANRQSAARSKERKIKYTGEL
    TabZIP111    LLDPKRAKRILANRQSAARSKERKIKYTGEL
    TabZIP131    LLDPKRAKRILANRQSAARSKERKIKYTGEL
```

I

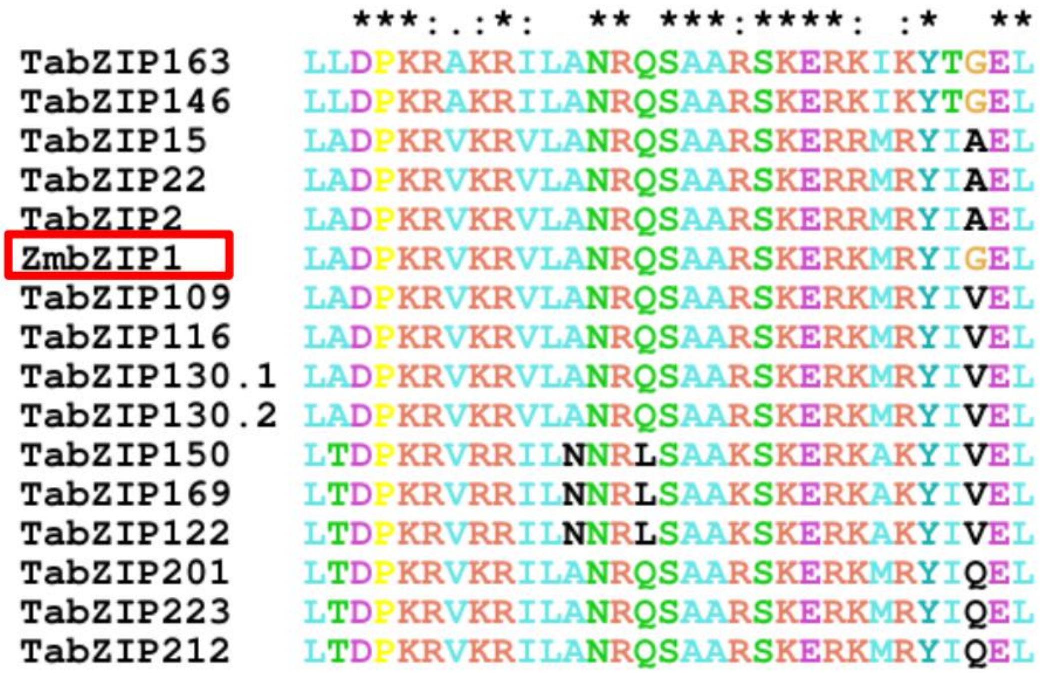
:.:*: ** ***:*: :* **
TabZIP163 LLDPKRAKRILANRQSAARSKERKIKYTGEL
TabZIP146 LLDPKRAKRILANRQSAARSKERKIKYTGEL
TabZIP15 LADPKRVKRVLANRQSAARSKERRMRYIAEL
TabZIP22 LADPKRVKRVLANRQSAARSKERRMRYIAEL
TabZIP2 LADPKRVKRVLANRQSAARSKERRMRYIAEL
ZmbZIP1 LADPKRVKRVLANRQSAARSKERKMRYIGEL
TabZIP109 LADPKRVKRVLANRQSAARSKERKMRYIVEL
TabZIP116 LADPKRVKRVLANRQSAARSKERKMRYIVEL
TabZIP130.1 LADPKRVKRVLANRQSAARSKERKMRYIVEL
TabZIP130.2 LADPKRVKRVLANRQSAARSKERKMRYIVEL
TabZIP150 LTDPKRVRRILNNRLSAAKSKERKAKYIVEL
TabZIP169 LTDPKRVRRILNNRLSAAKSKERKAKYIVEL
TabZIP122 LTDPKRVRRILNNRLSAAKSKERKAKYIVEL
TabZIP201 LTDPKRVKRILANRQSAARSKERKMRYIQEL
TabZIP223 LTDPKRVKRILANRQSAARSKERKMRYIQEL
TabZIP212 LTDPKRVKRILANRQSAARSKERKMRYIQEL

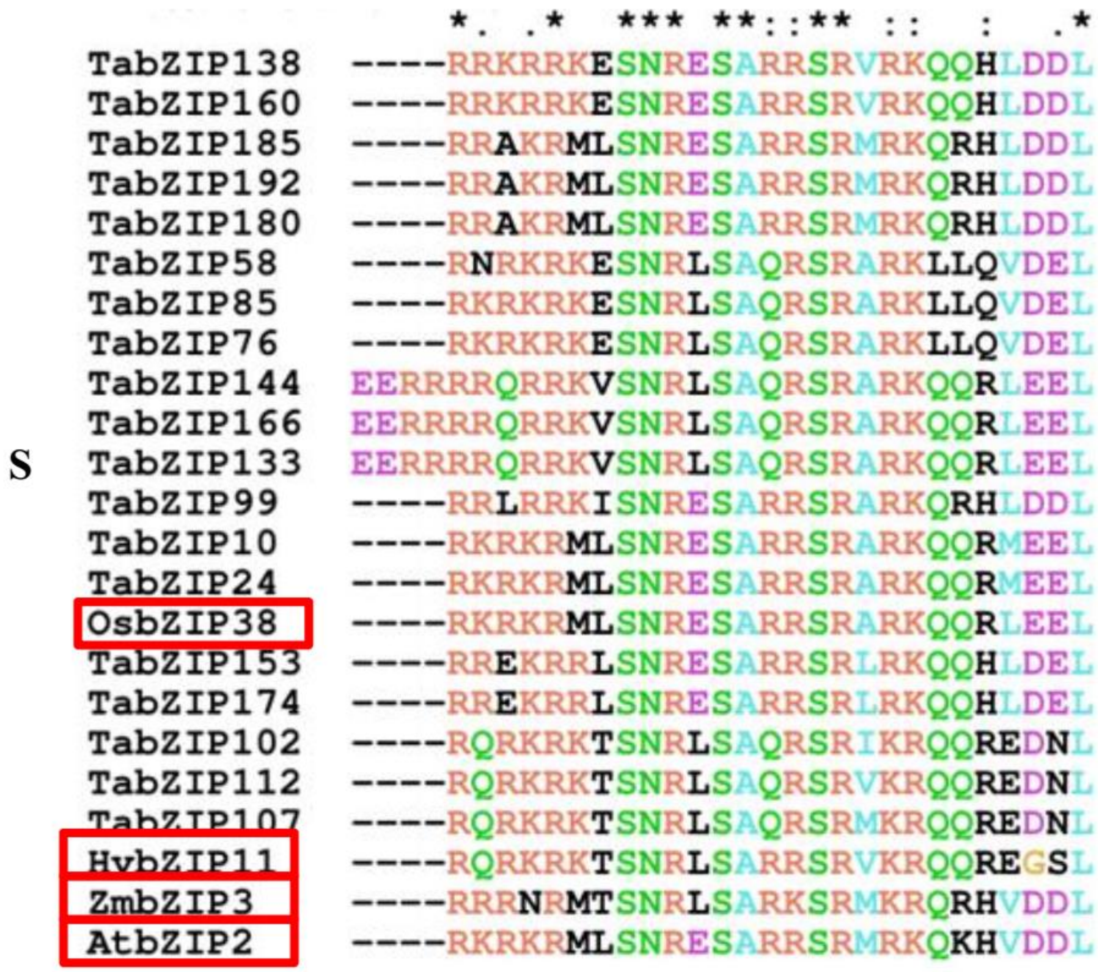
. . *** **::** ::   :   .*
TabZIP138 ----RRKRRKESNRESARRSRVRKQQHLDDL
TabZIP160 ----RRKRRKESNRESARRSRVRKQQHLDDL
TabZIP185 ----RRAKRMLSNRESARRSRMRKQRHLDDL
TabZIP192 ----RRAKRMLSNRESARRSRMRKQRHLDDL
TabZIP180 ----RRAKRMLSNRESARRSRMRKQRHLDDL
TabZIP58 ----RNRKRKESNRLSAQRSRARKLLQVDEL
TabZIP85 ----RKRKRKESNRLSAQRSRARKLLQVDEL
TabZIP76 ----RKRKRKESNRLSAQRSRARKLLQVDEL
TabZIP144 EERRRRQRRKVSNRLSAQRSRARKQQRLEEL
TabZIP166 EERRRRQRRKVSNRLSAQRSRARKQQRLEEL
S
TabZIP133 EERRRRQRRKVSNRLSAQRSRARKQQRLEEL
TabZIP99 ----RRLRRKISNRESARRSRARKQRHLDDL
TabZIP10 ----RKRKRMLSNRESARRSRARKQQRMEEL
TabZIP24 ----RKRKRMLSNRESARRSRARKQQRMEEL
OsbZIP38 ----RKRKRMLSNRESARRSRARKQQRLEEL
TabZIP153 ----RREKRRLSNRESARRSRLRKQQHLDEL
TabZIP174 ----RREKRRLSNRESARRSRLRKQQHLDEL
TabZIP102 ----RQRKRKTSNRLSAQRSRIKRQQREDNL
TabZIP112 ----RQRKRKTSNRLSAQRSRVKRQQREDNL
TabZIP107 ----RQRKRKTSNRLSAQRSRMKRQQREDNL
HvbZIP11 ----RQRKRKTSNRLSARRSRVKRQQREGSL
ZmbZIP3 ----RRRNRMTSNRLSARKSRMKRQRHVDDL
AtbZIP2 ----RKRKRMLSNRESARRSRMRKQKHVDDL

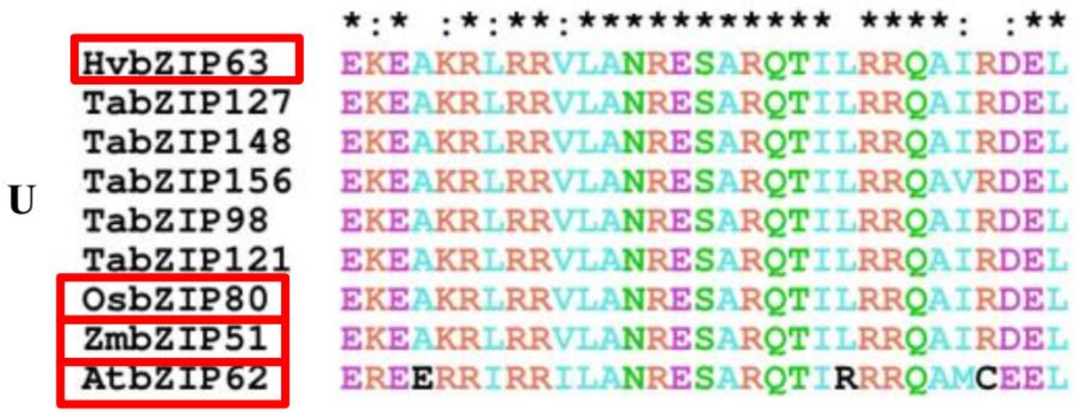

Supplementary Figure S1: Multiple sequence alignment (MSA) of basic and hinge regions of 370 wheat bZIP (TabZIP) proteins. They are classified into 11 groups (A to I, S and U).Alignment of amino acid code was generated using multiple sequence alignment with two homologs each of *Arabidopsis*(AtbZIP), maize (ZmbZIP), barley (HvbZIP), and rice (OsbZIP) along with TabZIP. The reference homologs were represented in red boxes and the asterisk (*) and colon (:) above the alignment represent the conserved and variable region, respectively.

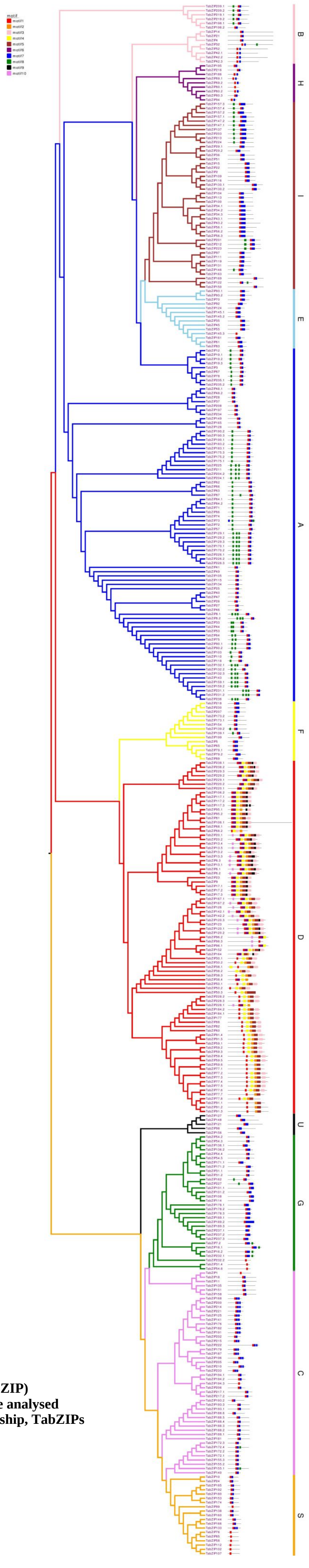

Supplementary Figure S2: The detail of identification and clustering of the conserved motifs of 370 wheat bZIP (TabZIP) proteins. Motif and phylogenetic analysis of TabZIPs were analysed using MEME database. Based on the phylogenetic relationship, TabZIPs were classified into 11 groups (A to I and S and U).

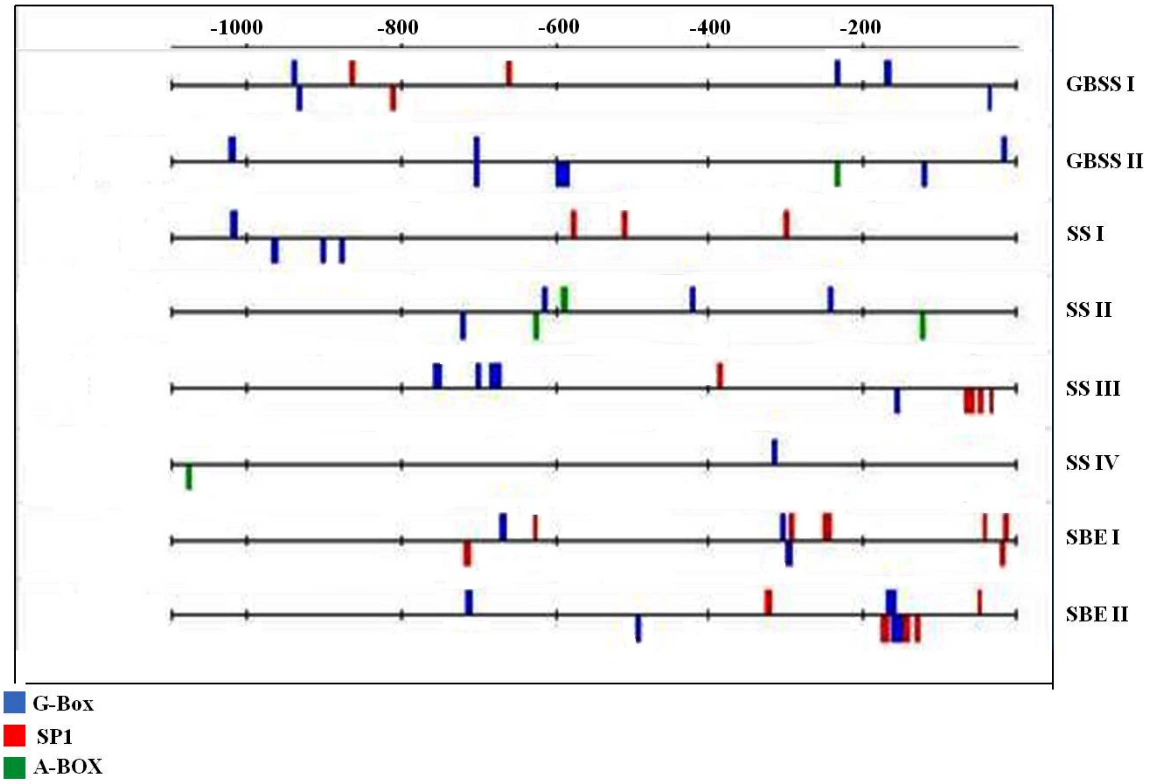

Supplementary Figure S3: Distribution of c*is*-regulatory elements in promoter regions of starch biosynthesis genes (GBSS I, GBSS II, SS I, SS II, SS III, SS IV, SBE I and SBE IIa). The mapping of the boxes was done on the sequences retrieved from International Genome Sequencing Consortium (https://www.wheatgenome.org/) and processed through Regulatory Sequence Analysis Tool (RSAT: http://rsat.ulb.ac.be/rsat/) considering upto 1kb upstream region of the genes.The A, G and SP1 boxes were represented by blue, green red color respectively.

Supplementary Figure S4. Multalin of amino acid sequences of GBSSI, SBE IIa and SBE IIb in ‘TAC 75’ high amylose, (amylose content - 64 %), ‘TAC 6’ low amylose (amylose content– 6.8 %), and parent variety, ‘C 306’ (amylose content - 26 %).

GBSSI 7AS

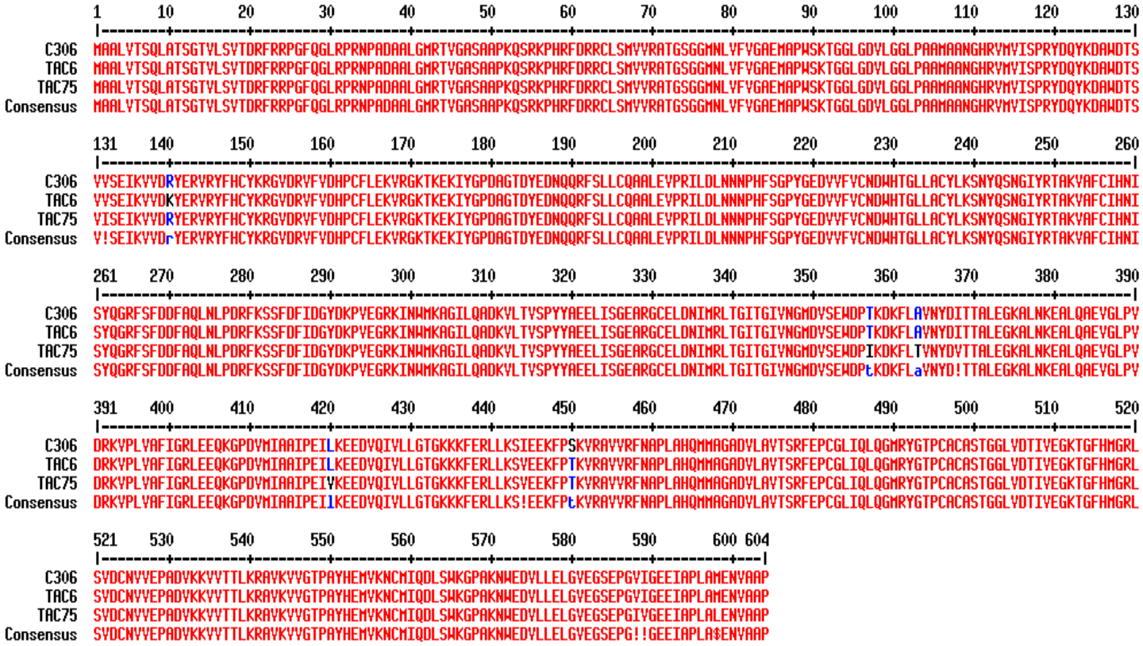

GBSSI 7DS

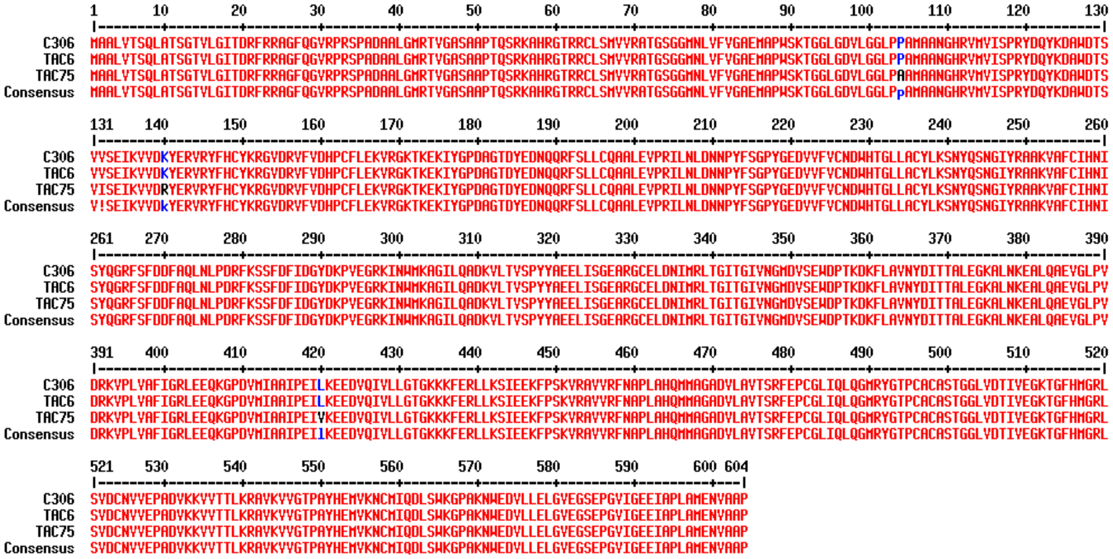

GBSSI 4AL

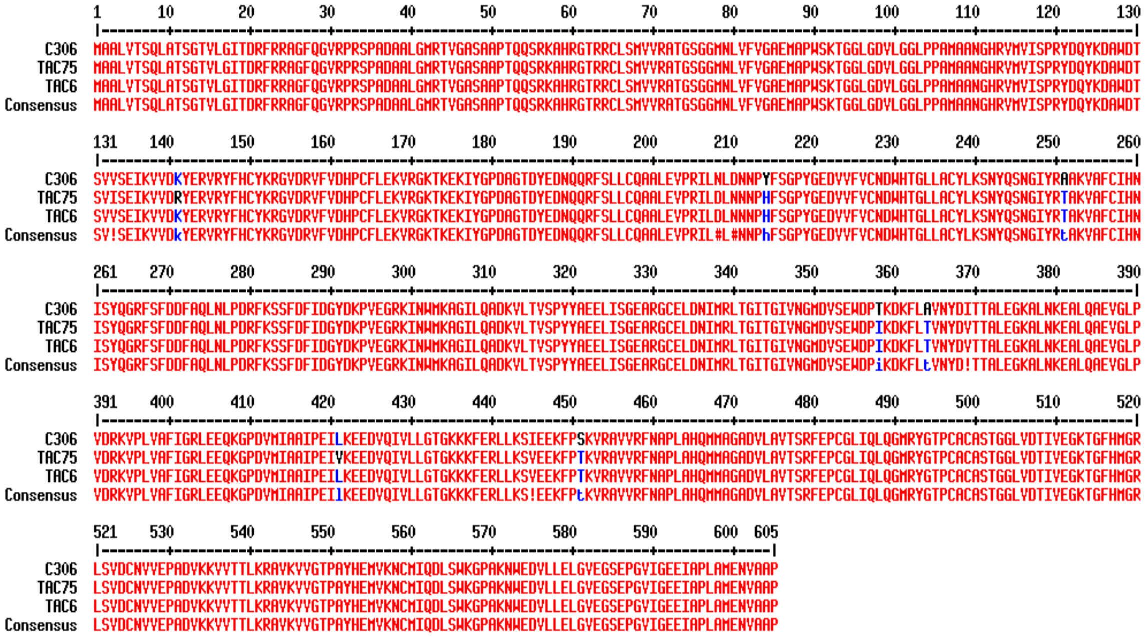

SBE IIa 2AL

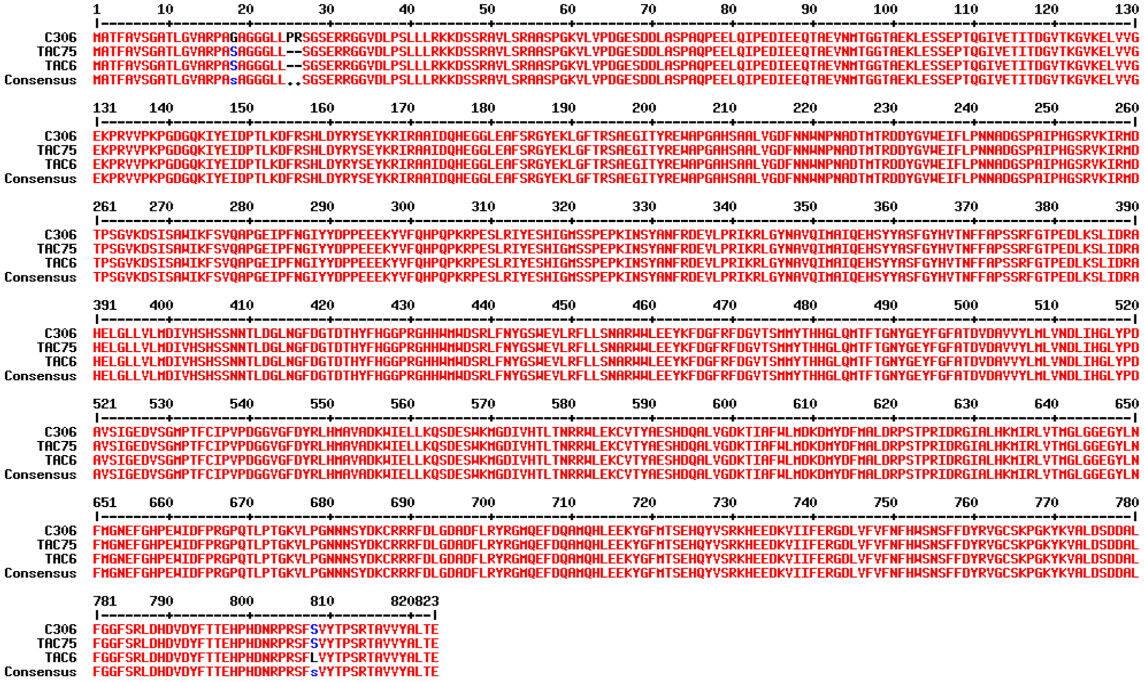

SBE IIa 2DL

```
          1       10        20        30        40        50        60        70        80        90        100       110       120       130
          |--------+---------+---------+---------+---------+---------+---------+---------+---------+---------+---------+---------+---------|
     C306 MATFAVSGATLGVARPASAGGGLLRSGSERRGGVDLPSLLLRKKDSSRAVLSRAASPGKVLVPDGESDDLASPAQPEELQIPEDIEEQTAEVNMTGGTAEKLESSEPTQGIVETITDGVTKGVKELVVGE
     TAC6 MATFAVSGATLGVARPASAGGGLLRSGSERRGGVDLPSLLLRKKDSSRAVLSRAASPGKVLVPDGESDDLASPAQPEELQIPEDIEEQTAEVNMTGGTAEKLESSEPTQGIVETITDGVTKGVKELVVGE
    TAC75 MATFAVSGATLGVAR---AGVGVLRSGSERRGGVDLPSLLLRKKDSSRAVLSRAASPGKVLVPDGESDDLASPAQPEELQIPEDIEEQTAEVNMTGGTAEKLESSEPTQGIVETITDGVTKGVKELVVGE
Consensus MATFAVSGATLGVARpasAGgGlLRSGSERRGGVDLPSLLLRKKDSSRAVLSRAASPGKVLVPDGESDDLASPAQPEELQIPEDIEEQTAEVNMTGGTAEKLESSEPTQGIVETITDGVTKGVKELVVGE

          131     140       150       160       170       180       190       200       210       220       230       240       250       260
          |--------+---------+---------+---------+---------+---------+---------+---------+---------+---------+---------+---------+---------|
     C306 KPRVVPKPGDGQKIYEIDPTLKDFRSHLDYRYSEYKRIRAAIDQHEGGLEAFSRGYEKLGFTRSAEGITYREWAPGAHSAALVGDFNNWNPNADTMTRDDYGVWEIFLPNNADGSPAIPHGSRVKIRMDT
     TAC6 KPRVVPKPGDGQKIYEIDPTLKDFRSHLDYRYSEYKRIRAAIDQHEGGLEAFSRGYEKLGFTRSAEGITYREWAPGAHSAALVGDFNNWNPNADTMTRDDYGVWEIFLPNNADGSPAIPHGSRVKIRMDT
    TAC75 KPRVVPKPGDGQKIYEIDPTLKDFRSHLDYRYSEYKRIRAAIDQHEGGLEAFSRGYEKLGFTRSAEGITYREWAPGAHSAALVGDFNNWNPNADTMTRDDYGVWEIFLPNNADGSPAIPHGSRVKIRMDT
Consensus KPRVVPKPGDGQKIYEIDPTLKDFRSHLDYRYSEYKRIRAAIDQHEGGLEAFSRGYEKLGFTRSAEGITYREWAPGAHSAALVGDFNNWNPNADTMTRDDYGVWEIFLPNNADGSPAIPHGSRVKIRMDT

          261     270       280       290       300       310       320       330       340       350       360       370       380       390
          |--------+---------+---------+---------+---------+---------+---------+---------+---------+---------+---------+---------+---------|
     C306 PSGVKDSISAWIKFSVQAPGEIPFNGIYYDPPEEEKYVFQHPQPKRPESLRIYESHIGMSSPEPKINSYANFRDEVLPRIKRLGYNAVQIMAIQEHSYYASFGYHVTNFFAPSSRFGTPEDLKSLIDRAH
     TAC6 PSGVKDSISAWIKFSVQAPGEIPFNGIYYDPPEEEKYVFQHPQPKRPESLRIYESHIGMSSPEPKINSYANFRDEVLPRIKRLGYNAVQIMAIQEHSYYASFGYHVTNFFAPSSRFGTPEDLKSLIDRAH
    TAC75 PSGVKDSISAWIKFSVQAPGEIPFNGIYYDPPEEEKYVFQHPQPKRPESLRIYESHIGMSSPEPKINSYANFRDEVLPRIKRLGYNAVQIMAIQEHSYYASFGYHVTNFFAPSSRFGTPEDLKSLIDRAH
Consensus PSGVKDSISAWIKFSVQAPGEIPFNGIYYDPPEEEKYVFQHPQPKRPESLRIYESHIGMSSPEPKINSYANFRDEVLPRIKRLGYNAVQIMAIQEHSYYASFGYHVTNFFAPSSRFGTPEDLKSLIDRAH

          391     400       410       420       430       440       450       460       470       480       490       500       510       520
          |--------+---------+---------+---------+---------+---------+---------+---------+---------+---------+---------+---------+---------|
     C306 ELGLLVLMDIVHSHSSNNTLDGLNGFDGTDTHYFHGGPRGHHWMWDSRLFNYGSWEVLRFLLSNARWWLEEYKFDGFRFDGVTSMMYTHHGLQMTFTGNYGEYFGFATDVDAVVYLMLVNDLIHGLYPDA
     TAC6 ELGLLVLMDIVHSHSSNNTLDGLNGFDGTDTHYFHGGPRGHHWMWDSRLFNYGSWEVLRFLLSNARWWLEEYKFDGFRFDGVTSMMYTHHGLQMTFTGNYGEYFGFATDVDAVVYLMLVNDLIHGLYPDA
    TAC75 ELGLLVLMDIVHSHSSNNTLDGLNGFDGTDTHYFHGGPRGHHWMWDSRLFNYGSWEVLRFLLSNARWWLEEYKFDGFRFDGVTSMMYTHHGLQMTFTGNYGEYFGFATDVDAVVYLMLVNDLIHGLYPDA
Consensus ELGLLVLMDIVHSHSSNNTLDGLNGFDGTDTHYFHGGPRGHHWMWDSRLFNYGSWEVLRFLLSNARWWLEEYKFDGFRFDGVTSMMYTHHGLQMTFTGNYGEYFGFATDVDAVVYLMLVNDLIHGLYPDA

          521     530       540       550       560       570       580       590       600       610       620       630       640       650
          |--------+---------+---------+---------+---------+---------+---------+---------+---------+---------+---------+---------+---------|
     C306 VSIGEDVSGMPTFCIPVPDGGVGFDYRLHMAVADKWIELLKQSDESWKMGDIVHTLTNRRWLEKCVTYAESHDQALVGDKTIAFWLMDKDMYDFMALDRPSTPRIDRGIALHKMIRLVTMGLGGEGYLNF
     TAC6 VSIGEDVSGMPTFCIPVPDGGVGFDYRLHMAVADKWIELLKQSDESWKMGDIVHTLTNRRWLEKCVTYAESHDQALVGDKTIAFWLMDKDMYDFMALDRPSTPRIDRGIALHKMIRLVTMGLGGEGYLNF
    TAC75 VSIGEDVSGMPTFCIPVPDGGVGFDYRLHMAVADKWIELLKQSDESWKMGDIVHTLTNRRWLEKCVTYAESHDQALVGDKTIAFWLMDKDMYDFMALDRPSTPRIDRGIALHKMIRLVTMGLGGEGYLNF
Consensus VSIGEDVSGMPTFCIPVPDGGVGFDYRLHMAVADKWIELLKQSDESWKMGDIVHTLTNRRWLEKCVTYAESHDQALVGDKTIAFWLMDKDMYDFMALDRPSTPRIDRGIALHKMIRLVTMGLGGEGYLNF

          651     660       670       680       690       700       710       720       730       740       750       760       770       780
          |--------+---------+---------+---------+---------+---------+---------+---------+---------+---------+---------+---------+---------|
     C306 MGNEFGHPEWIDFPRGPQTLPTGKVLPGNNNSYDKCRRRFDLGDADFLRYRGMQEFDQAMQHLEEKYGFMTSEHQYVSRKHEEDKVIIFERGDLVFVFNFHWSNSFFDYRVGCSKPGKYKVALDSDDALF
     TAC6 MGNEFGHPEWIDFPRGPQTLPTGKVLPGNNNSYDKCRRRFDLGDADFLRYRGMQEFDQAMQHLEEKYGFMTSEHQYVSRKHEEDKVIIFERGDLVFVFNFHWSNSFFDYRVGCSKPGKYKVALDSDDALF
    TAC75 MGNEFGHPEWIDFPRGPQTLPTGKVLPGNNNSYDKCRRRFDLGDADFLRYRGMQEFDQAMQHLEEKYGFMTSDHQYVSRKHEEDKVIVFEKGDLVFVFNFHWSNSYFDYRVGCLKPGKYKVVLDSDAGLF
Consensus MGNEFGHPEWIDFPRGPQTLPTGKVLPGNNNSYDKCRRRFDLGDADFLRYRGMQEFDQAMQHLEEKYGFMTS#HQYVSRKHEEDKVI!FErGDLVFVFNFHWSNS%FDYRVGCsKPGKYKVaLDSDdaLF

          781     790       800       810       82022
          |--------+---------+---------+---------+-|
     C306 GGFSRLDHDVDYFTTEHPHDNRPRSFSVYTPSRTAVVYALTE
     TAC6 GGFSRLDHDVDYFTTEHPHDNRPRSFLVYTPSRTAVVYALTE
    TAC75 GGFGRIHHTAEHFTSDCQHDNRPHSFSVYTPSRTCVVYAPMN
Consensus GGFsRldHdv#yFTt#hpHDNRPrSFsVYTPSRTaVVYAlt#
```

SBE IIa 2BL

```
          1       10        20        30        40        50        60        70        80        90        100       110       120       130
          |--------+---------+---------+---------+---------+---------+---------+---------+---------+---------+---------+---------+---------|
     C306 MATFAVSGATLGVARPAGAGGGLLRSGSERRGGVDLPSLLLRKKDSSRAVLSRAASPGKVLVPDGESDDLAASPAQPEELQIPEDIEEQTAEVNMTGGTAEKLESSEPTQGIVETITDGVTKGVKELVVG
    TAC75 MATFAVSGATLGVARPASAGGGLLRSGSERRGGVDLPSLLLRKKDSSRAVLSRAASPGKVLVPDGESDDLAASPAQPEELQIPEDIEEQTAEVNMTGGTAEKLESSEPTQGIVETITDGVTKGVKELVVG
     TAC6 MATFAVSGATLGVARPASAGGGLLRSGSERRGGVDLPSLLLRKKDSSRAVLSRAASPGKVLVPDGESDDLAATPAQPEELQIPEDIEEQTAEVNMTGGTAEKLQYSEPTQGIVETITDGVTKGVKELVVG
Consensus MATFAVSGATLGVARPAsAGGGLLRSGSERRGGVDLPSLLLRKKDSSRAVLSRAASPGKVLVPDGESDDLAAsPAQPEELQIPEDIEEQTAEVNMTGGTAEKL#sSEPTQGIVETITDGVTKGVKELVVG

          131     140       150       160       170       180       190       200       210       220       230       240       250       260
          |--------+---------+---------+---------+---------+---------+---------+---------+---------+---------+---------+---------+---------|
     C306 EKPRVVPKPGDGQKIYEIDPTLKDFRSHLDYRYSEYKRIRAAIDQHEGGLEAFSRGYEKLGFTRSAEGITYREWAPGAHSAALVGDFNNWNPNADTMTRDDYGVWEIFLPNNADGSPAIPHGSRVKIRMD
    TAC75 EKPRVVPKPGDGQKIYEIDPTLKDFRSHLDYRYSEYKRIRAAIDQHEGGLEAFSRGYEKLGFTRSAEGITYREWAPGAHSAALVGDFNNWNPNADTMTRDDYGVWEIFLPNNADGSPAIPHGSRVKIRMD
     TAC6 EKPRVVPKPGDGQKIYEIDPTLKDFRSHLDYRYSEYKRIRAAIDQHEGGLEAFSRGYEKLGFTRSAEGITYREWAPGAHSAALVGDFNNWNPNADTMTRDDYGVWEIFLPNNADGSPAIPHGSRVKIRMD
Consensus EKPRVVPKPGDGQKIYEIDPTLKDFRSHLDYRYSEYKRIRAAIDQHEGGLEAFSRGYEKLGFTRSAEGITYREWAPGAHSAALVGDFNNWNPNADTMTRDDYGVWEIFLPNNADGSPAIPHGSRVKIRMD

          261     270       280       290       300       310       320       330       340       350       360       370       380       390
          |--------+---------+---------+---------+---------+---------+---------+---------+---------+---------+---------+---------+---------|
     C306 TPSGVKDSISAWIKFSVQAPGEIPFNGIYYDPPEEEKYVFQHPQPKRPESLRIYESHIGMSSPEPKINSYANFRDEVLPRIKRLGYNAVQIMAIQEHSYYASFGYHVTNFFAPSSRFGTPEDLKSLIDRA
    TAC75 TPSGVKDSISAWIKFSVQAPGEIPFNGIYYDPPEEEKYVFQHPQPKRPESLRIYESHIGMSSPEPKINSYANFRDEVLPRIKRLGYNAVQIMAIQEHSYYASFGYHVTNFFAPSSRFGTPEDLKSLIDRA
     TAC6 TPSGVKDSISAWIKFSVQAPGEIPFNGIYYDPPEEEKYVFQHPQPKRPESLRIYESHIGMSSPEPKINSYANFRDEVLPRIKRLGYNAVQIMAIQEHSYYASFGYHVTNFFAPSSRFGTPEDLKSLIDRA
Consensus TPSGVKDSISAWIKFSVQAPGEIPFNGIYYDPPEEEKYVFQHPQPKRPESLRIYESHIGMSSPEPKINSYANFRDEVLPRIKRLGYNAVQIMAIQEHSYYASFGYHVTNFFAPSSRFGTPEDLKSLIDRA

          391     400       410       420       430       440       450       460       470       480       490       500       510       520
          |--------+---------+---------+---------+---------+---------+---------+---------+---------+---------+---------+---------+---------|
     C306 HELGLLVLMDIVHSHSSNNTLDGLNGFDGTDTHYFHGGPRGHHWMWDSRLFNYGSWEVLRFLLSNARWWLEEYKFDGFRFDGVTSMMYTHHGLQMTFTGNYGEYFGFATDVDAVVYLMLVNDLIHGLYPD
    TAC75 HELGLLVLMDIVHSHSSNNTLDGLNGFDGTDTHYFHGGPRGHHWMWDSRLFNYGSWEVLRFLLSNARWWLEEYKFDGFRFDGVTSMMYTHHGLQMTFTGNYGEYFGFATDVDAVVYLMLVNDLIHGLYPD
     TAC6 HELGLLVLMDIVHSHSSNNTLDGLNGFDGTDTHYFHGGPRGHHWMWDSRLFNYGSWEVLRFLLSNARWWLEEYKFDGFRFDGVTSMMYTHHGLQMTFTGNYGEYFGFATDVDAVVYLMLVNDLIHGLYPD
Consensus HELGLLVLMDIVHSHSSNNTLDGLNGFDGTDTHYFHGGPRGHHWMWDSRLFNYGSWEVLRFLLSNARWWLEEYKFDGFRFDGVTSMMYTHHGLQMTFTGNYGEYFGFATDVDAVVYLMLVNDLIHGLYPD

          521     530       540       550       560       570       580       590       600       610       620       630       640       650
          |--------+---------+---------+---------+---------+---------+---------+---------+---------+---------+---------+---------+---------|
     C306 AVSIGEDVSGMPTFCIPVPDGGVGFDYRLHMAVADKWIELLKQSDESWKMGDIVHTLTNRRWLEKCVTYAESHDQALVGDKTIAFWLMDKDMYDFMALDRPSTPRIDRGIALHKMIRLVTMGLGGEGYLN
    TAC75 AVSIGEDVSGMPTFCIPVPDGGVGFDYRLHMAVADKWIELLKQSDESWKMGDIVHTLTNRRWLEKCVTYAESHDQALVGDKTIAFWLMDKDMYDFMALDRPSTPRIDRGIALHKMIRLVTMGLGGEGYLN
     TAC6 AVSIGEDVSGMPTFALPVQVGGVGFDYRLHMAVADKWIELLKGNDEAWEMGNIVHTLTNRRWLEKCVTYAESHDQALVGDKTIAFWLMDKDMYDFMALDRPSTPRIDRGIALHKMIRLVTMGLGGEGYLN
Consensus AVSIGEDVSGMPTFciPVpdGGVGFDYRLHMAVADKWIELLKqsDEsWkMG#IVHTLTNRRWLEKCVTYAESHDQALVGDKTIAFWLMDKDMYDFMALDRPSTPRIDRGIALHKMIRLVTMGLGGEGYLN

          651     660       670       680       690       700       710       720       730       740       750       760       770       780
          |--------+---------+---------+---------+---------+---------+---------+---------+---------+---------+---------+---------+---------|
     C306 FMGNEFGHPEWIDFPRGPQTLPTGKVLPGNNNSYDKCRRRFDLGDADFLRYRGMQEFDQAMQHLEEKYGFMTSEHQYVSRKHEEDKVIIFERGDLVFVFNFHWSNSFFDYRVGCSKPGKYKVALDSDDAL
    TAC75 FMGNEFGHPEWIDFPRGPQTLPTGKVLPGNNNSYDKCRRRFDLGDADFLRYRGMQEFDQAMQHLEEKYGFMTSEHQYVSRKHEEDKVIIFERGDLVFVFNFHWSNSFFDYRVGCSKPGKYKVALDSDDAL
     TAC6 FMGNEFGHPEWIDFPRGPQTLPTGKVLPGNNNSYDKCRRRFDLGDADFLRYRGMQEFDQAMQHLEEKYGFMTSEHQYVSRKHEEDKVIIFERGDLVFVFNFHWSNSFFDYRVGCSKPGKYKVALDSDDAL
Consensus FMGNEFGHPEWIDFPRGPQTLPTGKVLPGNNNSYDKCRRRFDLGDADFLRYRGMQEFDQAMQHLEEKYGFMTSEHQYVSRKHEEDKVIIFERGDLVFVFNFHWSNSFFDYRVGCSKPGKYKVALDSDDAL

          781     790       800       810       820823
          |--------+---------+---------+---------+--|
     C306 FGGFSRLDHDVDYFTTEHPHDNRPRSFSVYTPSRTAVVYALTE
    TAC75 FGGFSRLDHDVDYFTTEHPHDNRPRSFSVYTPSRTAVVYALTE
     TAC6 FGGFSRLDHDVDYFTTEHPHDNRPRSFLVYTPSRTAVVYALTE
Consensus FGGFSRLDHDVDYFTTEHPHDNRPRSFsVYTPSRTAVVYALTE
```

SBE IIb 2AL

```
          1       10        20        30        40        50        60        70        80        90        100       110       120       130
          |--------+---------+---------+---------+---------+---------+---------+---------+---------+---------+---------+---------+---------|
     C306 MASPAFAVSAAGLARPSAPRSGGPERRGRGVELQSPSLLFGRNKGTRSPRAVGVGGSGWRVVMRAGGPSGEVMIPDGGSGGTPPSIDGPVQFDSDDLKVPFIDDETSLQDGGEDTIWSSETNQVTEEIDA
     TAC6 MASPAFAVSAAGLARPSAPRSGGPERRGRGVELQSPSLLFGRNKGTRSPRAVGVGGSGWRVVMRAGGPSGEVMIPDGGSGGTPPSIDGPVQFDSDDLKVPFIDDETSLQDGGEDSIWSSETNQVSEEIDA
    TAC75 MASPAFAVSAAGLARPSAPRSGGPERRGRGVELQSPSLLFGRNKGTRSPRAVGVGGSGWRVVMRAGGPSGEVMIPDGGSGGTPPSIDGPVQFDSDDLKVPFIDDETSLQDGGEDTIWSSETNQVTEEIDA
Consensus MASPAFAVSAAGLARPSAPRSGGPERRGRGVELQSPSLLFGRNKGTRSPRAVGVGGSGWRVVMRAGGPSGEVMIPDGGSGGTPPSIDGPVQFDSDDLKVPFIDDETSLQDGGEDtIWSSETNQVtEEIDA

          131     140       150       160       170       180       190       200       210       220       230       240       250       260
          |--------+---------+---------+---------+---------+---------+---------+---------+---------+---------+---------+---------+---------|
     C306 EGTSRMDKESSTGEKLRILPPPGNGQQIYEIDPTLRDFKYHLEYRYSLYRRIRSDIDEHEGGMDVFSRGYEKFGFVRSAEGITYREWAPGADSAALVGDFNNWDPNADHMSKNDLGIWEIFLPNNADGSP
     TAC6 EDTSRMDKESSTREKLRILPPPGNGQQIYEIDPTLRDFKYHLEYRYSLYRRIRSDIDEHEGGMDVFSRGYEKFGFVRSAEGITYREWAPGADSAALVGDFNNWDPNADHMSKNDLGIWEIFLPNNADGSP
    TAC75 EGTSRMDKESSTGEKLRILPPPGNGQQIYEIDPTLRDFKYHLEYRYSLYRRIRSDIDEHEGGMDVFSRGYEKFGFVRSAEGITYREWAPGADSAALVGDFNNWDPNADHMSKNDLGVWEIFLPNNADGSP
Consensus EgTSRMDKESSTgEKLRILPPPGNGQQIYEIDPTLRDFKYHLEYRYSLYRRIRSDIDEHEGGMDVFSRGYEKFGFVRSAEGITYREWAPGADSAALVGDFNNWDPNADHMSKNDLG!WEIFLPNNADGSP

          261     270       280       290       300       310       320       330       340       350       360       370       380       390
          |--------+---------+---------+---------+---------+---------+---------+---------+---------+---------+---------+---------+---------|
     C306 PIPHGSRVKVRMGTPSGTKDSIPAWIKYSVQTPGDIPYNGIYYDPPEEEKYVFKHPQPKRPKSLRIYETHVGMSSPEPKINTYANFRDEVLPRIKRLGYNAVQIMAIQEHSYYGSFGYHVTNFFAPSSRF
     TAC6 PIPHGSRVKVRMGTPSGTKDSIPAWIKYSVQTPGDIPYNGIYYDPPEEEKYVFKHPQPKRPKSLRIYETHVGMSSPEPKINTYANFRDEVLPRIKRLGYNAVQIMAIQEHSYYGSFGYHVTNFFAPSSRF
    TAC75 PIPHGSRVKVRMDTPSGIKDSIPAWIKYSVQTPGDIPYNGIYYDPPEEEKYVFKHPQPKRPKSLRIYETHVGMSSPEPKINTYANFRDEVLPRIKRLGYNAVQIMAIQEHSYYGSFGYHVTNFFAPSSRF
Consensus PIPHGSRVKVRMgTPSGtKDSIPAWIKYSVQTPGDIPYNGIYYDPPEEEKYVFKHPQPKRPKSLRIYETHVGMSSPEPKINTYANFRDEVLPRIKRLGYNAVQIMAIQEHSYYGSFGYHVTNFFAPSSRF

          391     400       410       420       430       440       450       460       470       480       490       500       510       520
          |--------+---------+---------+---------+---------+---------+---------+---------+---------+---------+---------+---------+---------|
     C306 GSPEDLKSLIDRAHELGLVVLMDVVHSHASNNTLDGLNGFDGTDTHYFHGGSRGHHWMWDSRVFNYGNKEVIRFLLSNARWWLEEYKFDGFRFDGATSMMYTHHGLQVTFTGSYHEYFGFATDVDAVVYL
     TAC6 GSPEDLKSLIDRAHELGLVVLMDVVHSHASNNTLDGLNGFDGTDTHYFHGGSRGHHWMWDSRVFNYGNKEVIRFLLSNARWWLEEYKFDGFRFDGATSMMYTHHGLQVTFTGSYHEYFGFATDVDAVVYL
    TAC75 GSPEDLKSLIDRAHELGLVVLMDVVHSHASNNTLDGLNGFDGTDTHYFHGGSRGHHWMWDSRVFNYGNKEVIRFLLSNARWWLEEYKFDGFRFDGATSMMYTHHGLQVTFTGSYHEYFGFATDVDAVVYL
Consensus GSPEDLKSLIDRAHELGLVVLMDVVHSHASNNTLDGLNGFDGTDTHYFHGGSRGHHWMWDSRVFNYGNKEVIRFLLSNARWWLEEYKFDGFRFDGATSMMYTHHGLQVTFTGSYHEYFGFATDVDAVVYL

          521     530       540       550       560       570       580       590       600       610       620       630       640       650
          |--------+---------+---------+---------+---------+---------+---------+---------+---------+---------+---------+---------+---------|
     C306 MLVNDLIHGFYPEAVTIGEDVSGMPTFALPVQVGGVGFDYRLHMAVADKWIELLKGNDEAWEMGNIVHTLTNRRWLEKCVTYAESHDQALVGDKTLAFWLMDKDMYDFMALNGPSTPNIDRGIALHKMIR
     TAC6 MLVNDLIHGFYPEAVTIGEDVSGMPTFALPVQVGGVGFDYRLHMAVADKWIELLKGNDEAWEMGNIVHTLTNRRWLEKCVTYAESHDQALVGDKTIAFWLMDKDMYDFMALNGPSTPNIDRGIALHKMIR
    TAC75 MLVNDLIHGLYPEAVTIGEDVSGMPTFALPVQVGGVGFDYRLHMAVADKWIELLKGNDEAWEMGNIVHTLTNRRWLEKCVTYAESHDQALVGDKTLAFWLMDKVMYDFMALDRPSTPRIDRGIALHKMIR
Consensus MLVNDLIHGfYPEAVTIGEDVSGMPTFALPVQVGGVGFDYRLHMAVADKWIELLKGNDEAWEMGNIVHTLTNRRWLEKCVTYAESHDQALVGDKTlAFWLMDKdMYDFMAL#gPSTPnIDRGIALHKMIR

          651     660       670       680       690       700       710       720       730       740       750       760       770       780
          |--------+---------+---------+---------+---------+---------+---------+---------+---------+---------+---------+---------+---------|
     C306 LITMGLGGEGYLNFMGNEFGHPEWIDFPRGPQVLPSGKFIPGNNNSYDKCRRRFDLGDAEFLRYHGMQQFDQAMQHLEEKYGFMTSDHQYVSRKHEEDKVIVFEKGDLVFVFNFHWSNSYFDYRVGCLKP
     TAC6 LITMGLGGEGYLNFMGNEFGHPEWIDFPRGPQVLPTGKFIPGNNNSYDKCRRRFDLGDAEFLRYHGMQQFDQAMQHLEEKYGFMTSDHQYVSRKHEEDKVIVFEKGDLVFVFNFHWSNSYFDYRVGCLKP
    TAC75 LVTMGLGGEGYLNFMGNEFGHPEWIDFPRGPQTLPTGKVLPGNNNSYDKCRRRFDLGDADFLRYRGMQEFDQAMQHLEEKYGFMTSDHQYVSRKHEEDKVIVFEKGDLVFVFNFHWSNSYFDYRVGCLKP
Consensus L!TMGLGGEGYLNFMGNEFGHPEWIDFPRGPQvLPtGKfiPGNNNSYDKCRRRFDLGDA#FLRYhGMQ#FDQAMQHLEEKYGFMTSDHQYVSRKHEEDKVIVFEKGDLVFVFNFHWSNSYFDYRVGCLKP

          781     790       800       810       820       830   836
          |--------+---------+---------+---------+---------+-----|
     C306 GKYKVVLDSDAGLFGGFGRIHHTAEHFTSDCQHDNRPHSFSVYTPSRTCVVYAPMN
     TAC6 GKYKVVLDSDAGLFGGFGRIHHTAEHFTSDCQHDNRPHSFSVYTPSRTCVVYAPMN
    TAC75 GKYKVVLDSDAGLFGGFGRIHHTAEHFTSDCQHDNRPHSFSVYTPSRTCVVYAPMN
Consensus GKYKVVLDSDAGLFGGFGRIHHTAEHFTSDCQHDNRPHSFSVYTPSRTCVVYAPMN
```

SBE IIb 2BL

```
          1       10        20        30        40        50        60        70        80        90        100       110       120       130
          |--------+---------+---------+---------+---------+---------+---------+---------+---------+---------+---------+---------+---------|
     C306 MPTFALPVQVGGVGFDYRLHMAVADKWIELLKGNDEAWEMGNIVHTLTNRRWLEKCVTYAESHDQALVGDKTLAFWLMDKDMYDFMALNGPSTPNIDRGIALHKMIRLITMGLGGEGYLNFMGNEFGHPE
    TAC75 MPTFALPVQVGGVGFDYRLHMAVADKWIELLKGNDEAWEMGNIVHTLTNRRWLEKCVTYAESHDQALVGDKTLAFWLMDKDMYDFMALNGPSTPNIDRGIALHKMIRLITMGLGGEGYLNFMGNEFGHPE
     TAC6 MPTFALPVQVGGVGFDYRLHMAVADKWIELLKGNDEAWEMGNIVHTLTNRRWLEKCVTYAESHDQALVGDKTIAFWLMDKDMYDFMALNGPSTPNIDRGIALHKMIRLITMGLGGEGYLNFMGNEFGHPE
Consensus MPTFALPVQVGGVGFDYRLHMAVADKWIELLKGNDEAWEMGNIVHTLTNRRWLEKCVTYAESHDQALVGDKTlAFWLMDKDMYDFMALNGPSTPNIDRGIALHKMIRLITMGLGGEGYLNFMGNEFGHPE

          131     140       150       160       170       180       190       200       210       220       230       240       250       260
          |--------+---------+---------+---------+---------+---------+---------+---------+---------+---------+---------+---------+---------|
     C306 WIDFPRGPQVLPSGKFIPGNNNSYDKCRRRFDLGDAEFLRYHGMQQFDQAMQHLEEKYGFMTSDHQYVSRKHEEDKVIVFEKGDLVFVFNFHWSNSYFDYRVGCLKPGKYKVVLDSDAGLFGGFGRIHHT
    TAC75 WIDFPRGPQVLPTGKFIPGNNNSYDKCRRRFDLGDAEFLRYHGMQQFDQAMQHLEEKYGFMTSDHQYVSRKHEEDKVIVFEKGDLVFVFNFHWSNSYFDYRVGCLKPGKYKVVLDSDAGLFGGFGRIHHT
     TAC6 WIDFPRGPQVLPTGKFIPGNNNSYDKCRRRFDLGDAEFLRYHGMQQFDQAMQHLEEKYGFMTSDHQYVSRKHEEDKVIVFEKGDLVFVFNFHWSNSYFDYRVGCLKPGKYKVVLDSDAGLFGGFGRIHHT
Consensus WIDFPRGPQVLPtGKFIPGNNNSYDKCRRRFDLGDAEFLRYHGMQQFDQAMQHLEEKYGFMTSDHQYVSRKHEEDKVIVFEKGDLVFVFNFHWSNSYFDYRVGCLKPGKYKVVLDSDAGLFGGFGRIHHT

          261     270       280       290293
          |--------+---------+---------+--|
     C306 AEHFTSDCQHDNRPHSFSVYTPSRTCVVYAPMN
    TAC75 AEHFTSDCQHDNRPHSFSVYTPSRTCVVYAPMN
     TAC6 AEHFTSDCQHDNRPHSFSVYTPSRTCVVYAPMN
Consensus AEHFTSDCQHDNRPHSFSVYTPSRTCVVYAPMN
```

SBE IIb 2DL

```
           1       10        20        30        40        50        60        70        80        90        100       110       120       130
           |--------+---------+---------+---------+---------+---------+---------+---------+---------+---------+---------+---------+---------|
     C306  MASPAFAVSAAGLARPSAPRSGGPERRGRGVELQSPSLLFGRNKGTRSPRAVGVGGSGWRVVMRAGGPSGEVMIPDGGSGGTPPSIDGPVQFDSDDLKVPFIDDETSLQDGGEDTIWSSETNQVTEEIDA
     TAC6  MASPAFAVSAAGLARPSAPRSGGPERRGRGVELQSPSLLFGRNKGTRSPRAVGVGGSGWRVVMRAGGPSGEVMIPDGGSGGTPPSIDGPVQFDSDDLKVPFIDDETSLQDGGEDSIWSSETNQVSEEIDA
    TAC75  MASPAFAVSAAGLARPSAPRSGGPERRGRGVELQSPSLLFGRNKGTRSPRAVGVGGSGWRVVMRAGGPSGEVMIPDGGSGGTPPSIDGPVQFDSDDLKVPFIDDETSLQDGGEDTIWSSETNQVTEEIDA
Consensus  MASPAFAVSAAGLARPSAPRSGGPERRGRGVELQSPSLLFGRNKGTRSPRAVGVGGSGWRVVMRAGGPSGEVMIPDGGSGGTPPSIDGPVQFDSDDLKVPFIDDETSLQDGGEDtIWSSETNQVtEEIDA

           131     140       150       160       170       180       190       200       210       220       230       240       250       260
           |--------+---------+---------+---------+---------+---------+---------+---------+---------+---------+---------+---------+---------|
     C306  EGTSRMDKESSTGEKLRILPPPGNGQQIYEIDPTLRDFKYHLEYRYSLYRRIRSDIDEHEGGMDVFSRGYEKFGFVRSAEGITYLEWAPGADSAALVGDFNNWDPNADHMSKNDLGIWEIFLPNNADGSP
     TAC6  EDTSRMDKESSTREKLRILPPPGNGQQIYEIDPTLRDFKYHLEYRYSLYRRIRSDIDEHEGGMDVFSRGYEKFGFVRSAEGITYREWAPGADSAALVGDFNNWDPNADHMSKNDLGIWEIFLPNNADGSP
    TAC75  EGTSRMDKESSTGEKLRILPPPGNGQQIYEIDPTLRDFKYHLEYRYSLYRRIRSDIDEHEGGMDVFSRGYEKFGFVRSAEGITYREWAPGADSAALVGDFNNWDPNADHMSKNDLGVWEIFLPNNADGSP
Consensus  EgTSRMDKESSTgEKLRILPPPGNGQQIYEIDPTLRDFKYHLEYRYSLYRRIRSDIDEHEGGMDVFSRGYEKFGFVRSAEGITYrEWAPGADSAALVGDFNNWDPNADHMSKNDLG!WEIFLPNNADGSP

           261     270       280       290       300       310       320       330       340       350       360       370       380       390
           |--------+---------+---------+---------+---------+---------+---------+---------+---------+---------+---------+---------+---------|
     C306  PIPHGSRVKVRMDTPSGIKDSIPAWIKYSVQTPGDIPYNGIYYDPPEEEKYVFKHPQPKRPKSLRIYETHVGMSSPEPKINTYANFRDEVLPRIKRLGYNAVQIMAIQEHSYYGSFGYHVTNFFAPSSRF
     TAC6  PIPHGSRVKVRMGTPSGTKDSIPAWIKYSVQTPGDIPYNGIYYDPPEEEKYVFKHPQPKRPKSLRIYETHVGMSSPEPKINTYANFRDEVLPRIKRLGYNAVQIMAIQEHSYYGSFGYHVTNFFAPSSRF
    TAC75  PIPHGSRVKVRMDTPSGIKDSIPAWIKYSVQTPGDIPYNGIYYDPPEEEKYVFKHPQPKRPKSLRIYETHVGMSSPEPKINTYANFRDEVLPRIKRLGYNAVQIMAIQEHSYYGSFGYHVTNFFAPSSRF
Consensus  PIPHGSRVKVRMdTPSGiKDSIPAWIKYSVQTPGDIPYNGIYYDPPEEEKYVFKHPQPKRPKSLRIYETHVGMSSPEPKINTYANFRDEVLPRIKRLGYNAVQIMAIQEHSYYGSFGYHVTNFFAPSSRF

           391     400       410       420       430       440       450       460       470       480       490       500       510       520
           |--------+---------+---------+---------+---------+---------+---------+---------+---------+---------+---------+---------+---------|
     C306  GSPEDLKSLIDRAHELGLVVLMDVVHSHASNNTLDGLNGFDGTDTHYFHGGSRGHHWMWDSRVFNYGNKEVIRFLLSNARWWLEEYKFDGFRFDGATSMMYTHHGLQVTFTGSYHEYFGFATDVDAVVYL
     TAC6  GSPEDLKSLIDRAHELGLVVLMDVVHSHASNNTLDGLNGFDGTDTHYFHGGSRGHHWMWDSRVFNYGNKEVIRFLLSNARWWLEEYKFDGFRFDGATSMMYTHHGLQVTFTGSYHEYFGFATDVDAVVYL
    TAC75  GSPEDLKSLIDRAHELGLVVLMDVVHSHASNNTLDGLNGFDGTDTHYFHGGSRGHHWMWDSRVFNYGNKEVIRFLLSNARWWLEEYKFDGFRFDGATSMMYTHHGLQVTFTGSYHEYFGFATDVDAVVYL
Consensus  GSPEDLKSLIDRAHELGLVVLMDVVHSHASNNTLDGLNGFDGTDTHYFHGGSRGHHWMWDSRVFNYGNKEVIRFLLSNARWWLEEYKFDGFRFDGATSMMYTHHGLQVTFTGSYHEYFGFATDVDAVVYL

           521     530       540       550       560       570       580       590       600       610       620       630       640       650
           |--------+---------+---------+---------+---------+---------+---------+---------+---------+---------+---------+---------+---------|
     C306  MLVNDLIHGFYPEAVTIGEDVSGMPTFALPVQVGGVGFDYRLHMAVADKWIELLKGNDEAWEMGNIVHTLTNRRWLEKCVTYAESHDQALVGDKTLAFWLMDKDMYDFMALNGPSTPNIDRGIALHKMIR
     TAC6  MLVNDLIHGFYPEAVTIGEDVSGMPTFALPVQVGGVGFDYRLHMAVADKWIELLKGNDEAWEMGNIVHTLTNRRWLEKCVTYAESHDQALVGDKTIAFWLMDKDMYDFMALNGPSTPNIDRGIALHKMIR
    TAC75  MLVNDLIHGLYPEAVTIGEDVSGMPTFALPVQVGGVGFDYRLHMAVADKWIELLKGNDEAWEMGNIVHTLTNRRWLEKCVTYAESHDQALVGDKTLAFWLMDKVMYDFMALDRPSTPRIDRGIALHKMIR
Consensus  MLVNDLIHGfYPEAVTIGEDVSGMPTFALPVQVGGVGFDYRLHMAVADKWIELLKGNDEAWEMGNIVHTLTNRRWLEKCVTYAESHDQALVGDKTlAFWLMDKdMYDFMAL#gPSTPnIDRGIALHKMIR

           651     660       670       680       690       700       710       720       730       740       750       760       770       780
           |--------+---------+---------+---------+---------+---------+---------+---------+---------+---------+---------+---------+---------|
     C306  LITMGLGGEGYLNFMGNEFGHPEWIDFPRGPQVLPSGKFIPGNNNSYDKCRRRFDLGDAEFLRYHGMQQFDQAMQHLEEKYGFMTSDHQYVSRKHEEDKVIVFEKGDLVFVFNFHWSNSYFDYRVGCLKP
     TAC6  LITMGLGGEGYLNFMGNEFGHPEWIDFPRGPQVLPTGKFIPGNNNSYDKCRRRFDLGDAEFLRYHGMQQFDQAMQHLEEKYGFMTSDHQYVSRKHEEDKVIVFEKGDLVFVFNFHWSNSYFDYRVGCLKP
    TAC75  LVTMGLGGEGYLNFMGNEFGHPEWIDFPRGPQTLPTGKVLPGNNNSYDKCRRRFDLGDADFLRYRGMQEFDQAMQHLEEKYGFMTSDHQYVSRKHEEDKVIVFEKGDLVFVFNFHWSNSYFDYRVGCLKP
Consensus  L!TMGLGGEGYLNFMGNEFGHPEWIDFPRGPQvLPtGKfiPGNNNSYDKCRRRFDLGDA#FLRYhGMQ#FDQAMQHLEEKYGFMTSDHQYVSRKHEEDKVIVFEKGDLVFVFNFHWSNSYFDYRVGCLKP

           781     790       800       810       820       830    836
           |--------+---------+---------+---------+---------+-----|
     C306  GKYKVVLDSDAGLFGGFGRIHHTAEHFTSDCQHDNRPHSFSVYTPSRTCVVYAPMN
     TAC6  GKYKVVLDSDAGLFGGFGRIHHTAEHFTSDCQHDNRPHSFSVYTPSRTCVVYAPMN
    TAC75  GKYKVVLDSDAGLFGGFGRIHHTAEHFTSDCQHDNRPHSFSVYTPSRTCVVYAPMN
Consensus  GKYKVVLDSDAGLFGGFGRIHHTAEHFTSDCQHDNRPHSFSVYTPSRTCVVYAPMN
```

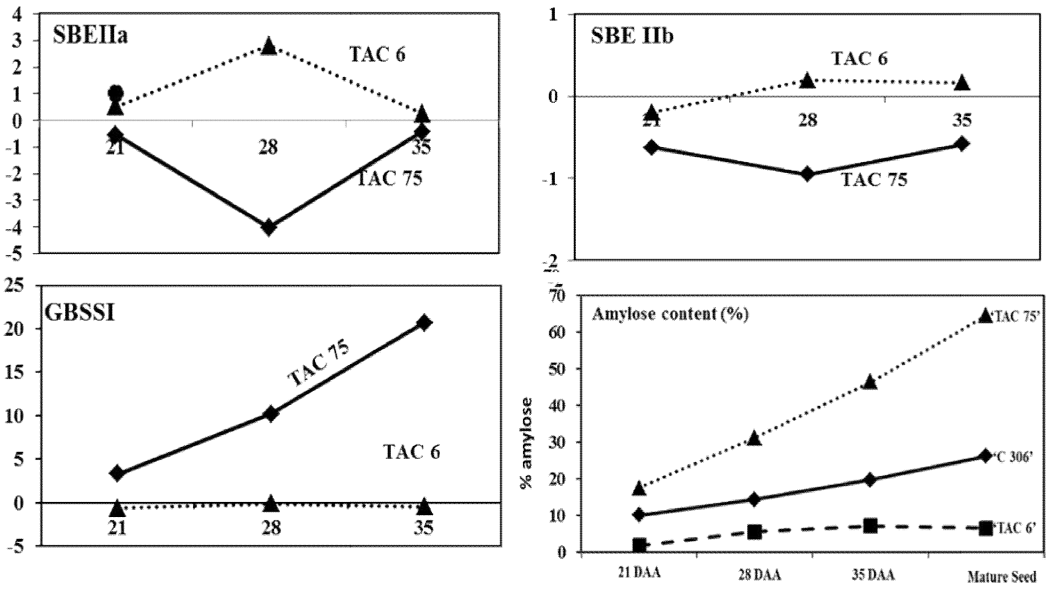

Supplementary Figure S5. Real-time quantitative expression data (Log$_2$ of fold change) of GBSSI, SBE IIa and SBE IIb during seed development of two mutant lines, 'TAC 75'(amylose content - 64 %) and 'TAC 6'(amylose content– 6.8 %), in comparison to the parent variety, 'C 306' (amylose content - 26 %) at seed development stages 21, 28, and 35 days after anthesis (DAA). The amylose content was measured in the above mutant variety in developing seeds (21, 28, 35 and mature).
